# Visible‐Light‐Driven Intermolecular Reductive Ene–Yne Coupling by Iridium/Cobalt Dual Catalysis for C(sp^3^)−C(sp^2^) Bond Formation

**DOI:** 10.1002/chem.201903708

**Published:** 2019-11-08

**Authors:** María J. González, Bernhard Breit

**Affiliations:** ^1^ Institut für Organische Chemie Albert-Ludwigs-Universität Freiburg Albertstr. 21 79104 Freiburg Germany

**Keywords:** alkynes, cobalt, dual catalysis, photoredox, reductive coupling

## Abstract

A new methodology to form C(sp^3^)−C(sp^2^) bonds by visible‐light‐driven intermolecular reductive ene–yne coupling has been successfully developed. The process relies on the ability of the Hantzsch ester to contribute in both SET and HAT processes through a unified cobalt and iridium catalytic system. This procedure avoids the use of stoichiometric amounts of reducing metallic reagents, which is translated into high functional‐group tolerance and atom economy.

## Graphical abstract

**A new methodology** to form C(sp^3^)−C(sp^2^) bonds via visible‐light‐driven intermolecular reductive ene–yne coupling has been developed. This procedure avoids the use of stoichiometric amounts of reducing metallic reagents which is translated into high functional group tolerance and atom‐economy (see scheme).

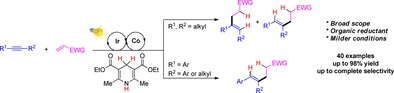

The development of new methodologies for selective C−C bond formation in a highly atom‐economical way is one of the most relevant and evolving research areas in organic synthesis.^1^ Particularly, the transition‐metal‐catalyzed reductive coupling of easily available, inexpensive, and bench‐stable π components provides a straightforward route to achieve this goal.1b, 2 However, the available methodologies require stoichiometric amounts of metallic reductants, such as Zn powder, silanes, boranes or Grignard reagents. In this context, Chen et al. reported in 2002 the cobalt‐catalyzed intermolecular reductive coupling of alkynes with conjugated alkenes in a highly chemo‐, regio‐, and stereoselective fashion (Scheme 1 a).^3^ This pioneering result is significant, because typical cobalt‐catalyzed C−C reactivities, such as cyclotrimerization4a–4c and carbonylation,4b–4c did not ensue. Notwithstanding the power of this approach, it struggles with low scope and the need of Zn powder in (super)stoichiometric amounts as reducing agent to generate active low‐valent Co^I^ species. Thus, the introduction of catalytic activation modes that are environmentally benign and capable of achieving a higher degree of structural diversity are highly desirable in this topic.

**Scheme 1:**
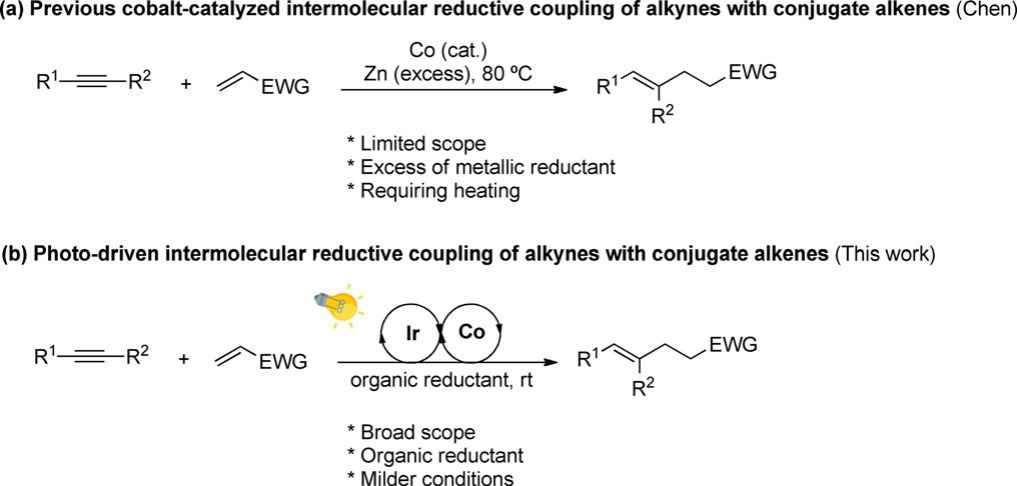
Cobalt‐catalyzed intermolecular reductive ene–yne coupling. EWG=electron‐withdrawing group.

Over the past decade, the renowned field of photochemistry is retrieving a central role in synthetic endeavors.^5^ Especially, the fast‐moving area of photoredox catalysis has witnessed dramatic developments, which have enabled previously inaccessible or inefficient transformations.^6^ Recently, Rovis et al. have explored the combination of photoredox catalysis with cobalt catalysis to access low‐valent cobalt(I) or cobalt(0) intermediates for the construction of arenes7a and in the hydroaminoalkylation of conjugated dienes.7b–7c These cobalt intermediates are traditionally generated in situ by using strong reducing conditions, such as heterogeneous metals or Grignard reagents, due to their synthetic challenge and poor stability.2f, 8 However, in their study, a tertiary amine is employed as a sacrificial organic reductant in a visible‐light‐driven photoredox cycle, overcoming the problem of limited functional group tolerance. Later, Zhao, Wu et al. made use of this protocol for the hydrocarboxylation and carboxylation of alkynes using CO_2_.^9^ Nevertheless, despite of its potential in C−C bond construction, this methodology has not been further developed.

Considering the lack of methodologies to achieve C(sp^3^)−C(sp^2^) bonds with both high atom economy and broad functional‐group tolerance,^2^ we envisioned that a photopromoted reaction pathway may provide opportunities to achieve new structural diversity in an environmentally compatible fashion. Herein, we report our findings on an unprecedented visible‐light‐driven intermolecular reductive ene–yne coupling via cobalt/iridium dual catalysis (Scheme 1 b).^10^, ^11^

To evaluate the feasibility of this hypothesis, easily accessible 2‐octyne (**1 a**) and ethyl acrylate (**2 a**) were selected as model substrates (Table 1). At the outset of our investigations, we used the following reaction conditions: CoBr_2_ (10 mol %), dppp (10 mol %),^12^ Ir(dF(CF_3_)ppy)_2_(dtb‐bpy)PF_6_ (0.5 mol %) as photocatalyst (PC), *N*‐ethyldiisopropylamine (DIPEA; 3.0 equiv) as sacrificial organic reductant and CsOPiv (0.5 equiv) as a base in MeCN under irradiation with a 4.8 W Blue LED strip (entry 1). Under these conditions, we observed the formation of regioisomers **3 a/4 a** in low yields and moderate (70:30) selectivity with **3 a** as the major product. Regrettably, after testing different bases and solvents, the previous result could not be improved (see Table S1 in the Supporting Information).

**Table 1:** Initial findings and reaction conditions optimization.^[a,b]^

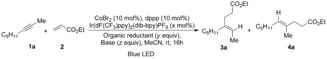						
Entry	*x*	Reductant	*y*	Base	*z*	Yield [%]^[c,d]^
1	0.5	DIPEA	3.0	CsOpiv	0.5	13
2	0.5	HE	1.5	CsOpiv	0.5	9
3	0.5	HE	1.5	Et_3_N	3.0	n.r.
4	0.5	HE	1.5	Pyridine	6.0	46
5	0.5	HE	1.5	DMAP	3.0	73
6	2.0	HE	1.5	DMAP	3.0	80

[a] Optimizations were performed on a 0.2 mmol scale using **1 a** (1.0 equiv), **2 a** (4.0 equiv), CoBr_2_ (10 mol %), dppp (10 mol %) in MeCN over a period of 16 h under irradiation with a 4.8 W Blue LED strip. [b] For further details on the optimization conditions, see the Supporting Information. [c] Isolated yield. [d] Compounds **3 a/4 a** were always obtained in a (70:30) selectivity determined by ^1^H NMR analysis of the crude mixture. n.r.=no reaction.

Then, we turned our attention to easily accessible and bench‐stable Hantzsch esters (HEs),^13^ which over the last decade have emerged as key electron^14^ and proton^15^ donors in a variety of challenging photoredox reactions. HEs can be readily converted in to the corresponding pyridines by means of a stepwise pathway by either single electron transfer (SET) followed by hydrogen atom transfer (HAT) or vice versa.^16^ Besides, their oxidative potential is typically 0.8–0.9 V versus SCE,^17^ suggesting that they have redox properties comparable to those of amines^18^ (*E*_1/2_^red^=0.8–1.0 vs. SCE). Gratifyingly, when using Hantzsch ester (HE) as organic reductant together with 4‐(dimethylamino)pyridine (DMAP) as base, **3 a/4 a** were obtained in a 73 % yield (entry 5). Other commercially available pyridines tested proved to be less effective than DMAP. Increasing the PC loading to 2.0 mol % the yield was improved to 80 % without affecting the selectivity (entry 6). Tests reactions in the absence of CoBr_2_, PC, HE, DMAP, and Blue LED were performed proving that all the additives are required for the transformation (see Table S3 in the Supporting Information).

By using these optimized conditions, the scope of this metallophotoredox reductive ene–yne coupling was first evaluated for alkyl–alkyl substituted alkynes (Table 2). 2‐Octyne in the presence of ethyl acrylate gave regioisomers **3 a/4 a** in good yield and moderate selectivity. However, when using *tert*‐butyl acrylate, both yield and selectivity decrease (**3 b/4 b**). It should be noticed that with a bulky substituent, such as isopropyl at R^1^, only product **3 c** was obtained albeit in lower yield (the bulkier *tert*‐butyl substituent was unreactive under these conditions). Symmetric alkyne 4‐octyne delivered **3 d** and **3 e** in 70 % and 43 % yields, respectively. Silyl ether protected alcohols were tolerated giving **3 g/4 g** in good yield and moderate selectivity. Remarkably, compound **4 f** was obtained as a single product in a 65 % yield probably due to steric hinderance. The reaction also took place in the presence of an ester group giving **4 h/3 h** in moderate yield and selectivity. It should be noted that in this case, the regioselectivity shifts being **4 h** the major product, which can be associated to steric impediments as in **4 f**.

**Table 2:** Scope of visible‐light‐driven intermolecular reductive ene–yne coupling with alkyl–alkyl substituted alkynes.

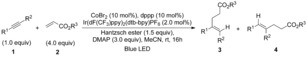
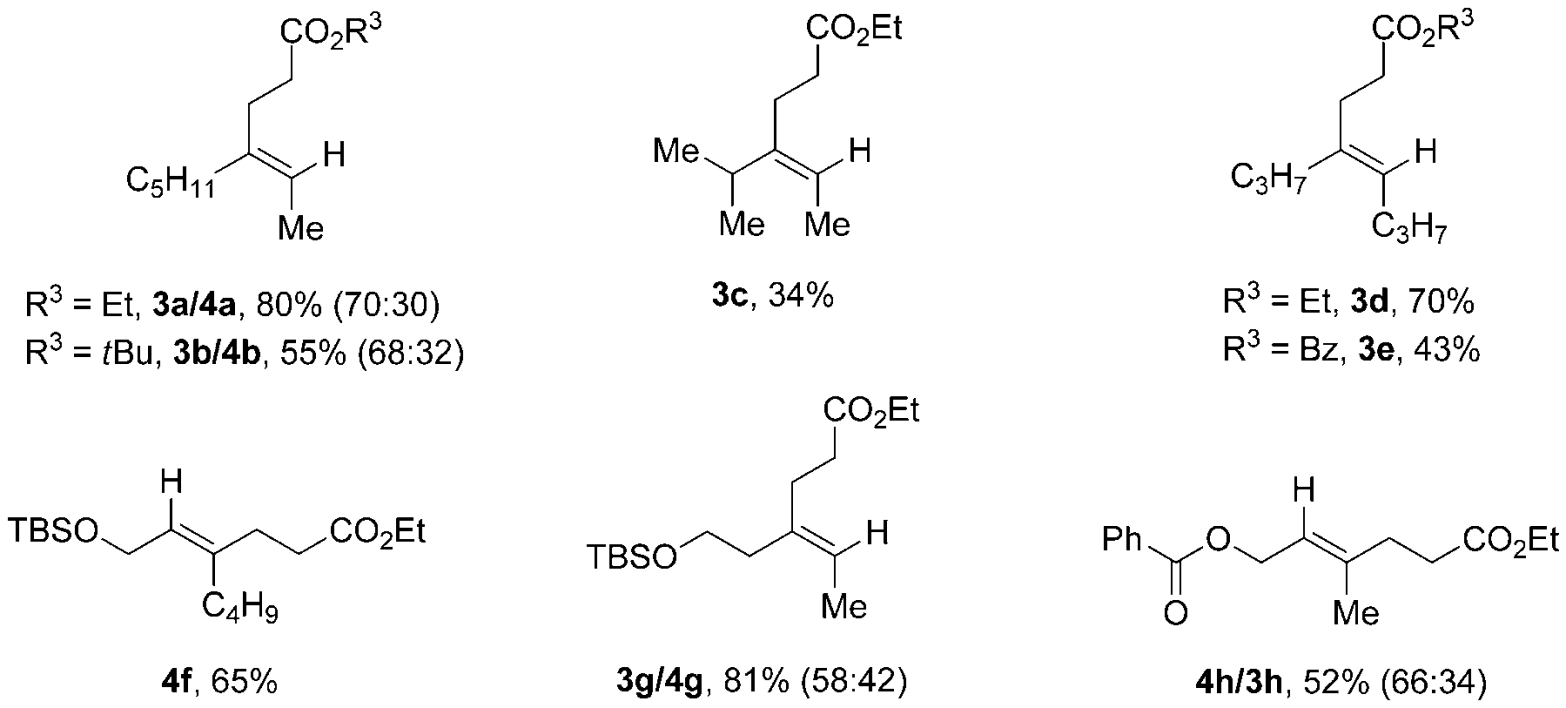

Combined isolated yield. Selectivity was determined by ^1^H NMR analysis of the crude mixture. Structures of major isomer are represented.

A subsequent screening of ligands to improve the selectivity revealed that xantphos (4,5‐bis(diphenylphosphino)‐9,9‐dimethylxanthene) is a better ligand when using aryl substituted alkynes affording isomers ***E*** **/*Z*** (see Tables S4 and S5 in the Supporting Information). Thus, 1‐aryl‐1‐akyl substituted alkynes were studied employing xantphos as ligand (Table 3). Under these conditions, 1‐phenyl‐1‐propyne afforded **6 a/7 a** in very good combined yield and selectivity. Other tested acrylates gave the corresponding derivatives in similar good yields and selectivity (**6 b**–**f/7 b**–**f**). Interestingly, single isomer **6 g** was obtained when treated 1‐phenyl‐1‐propyne with acrylonitrile yet in a 35 % yield. The reaction tolerated aryl groups bearing methyl (**6 h/7 h**), trifluoromethyl (**6 i/7 i**), bromine (**6 j/7 j**), and ester (**6 k/7 k**) substituents in good yields and moderate selectivity. Other aryl groups, such as naphthyl (**6 l/7 l**), thiophen (**6 m/7 m**), and 1,3‐benzodioxole (**6 n/7 n**) were also reactive in low to moderate yields and selectivities. Different substituents at position two like propyl (**6 o/7 o**), benzyl (**6 p/7 p**), methoxymethyl (**6 q**), allyl (**6 r/7 r**) and silyl ether protected alcohol (**6 s/7 s**) proved also suitable. Remarkably, when using (3‐methoxyprop‐1‐yn‐1‐yl)benzene compound **6 q** was obtained as a single isomer.

**Table 3:** Scope of visible‐light‐driven intermolecular reductive ene–yne coupling with 1‐aryl‐1‐alkyl substituted alkynes.

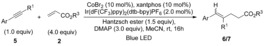
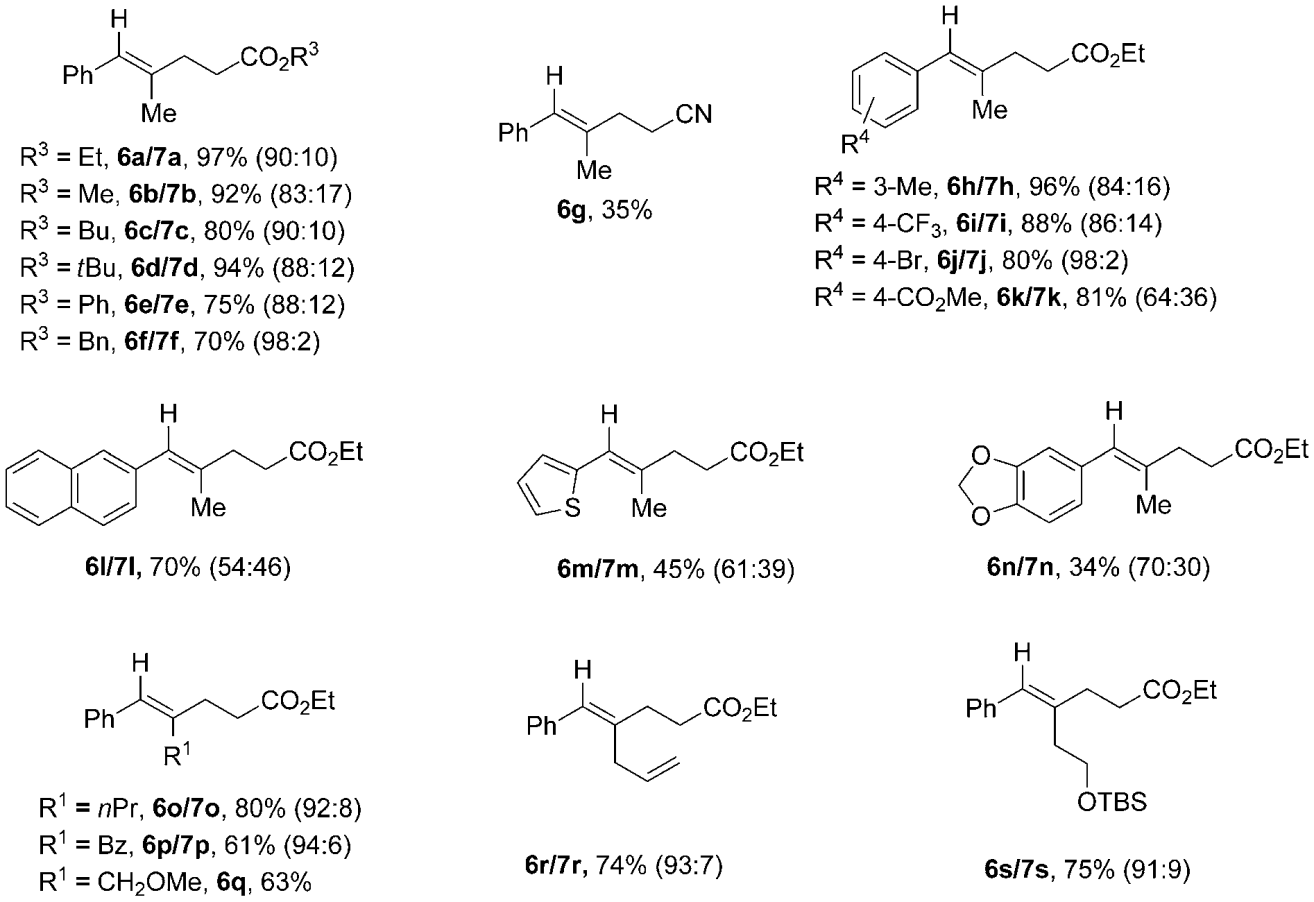

Combined isolated yield. Selectivity was determined by ^1^H NMR analysis of the crude mixture. Structures of major isomer are represented.

The reaction was also tested with internal diaryl substituted alkynes (Table 4). Under these conditions, tolan afforded isomers **9 a/10 a** in very good combined yield and moderate selectivity. Methyl acrylate gave similar results (**9 b/10 b**), whereas with bulkier R^3^ substituents, such as butyl, *tert*‐butyl, and phenyl, the yields decreased (**9 c**–**e/10 c**–**e**). As was expected, in the case of unsymmetrical 1‐(phenylethynyl)‐4‐(trifluoromethyl)benzene), the acrylate was added regioselectively to the most electron rich carbon (**9 f/10 f**). Remarkably, when using electron‐poor diaryl alkynes isomers **9 g**–**i** and **10 g**–**i** could be separated.

**Table 4:** Scope of visible‐light‐driven intermolecular reductive ene‐yne coupling with internal diaryl substituted alkynes.

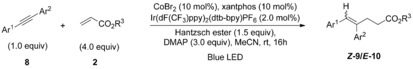
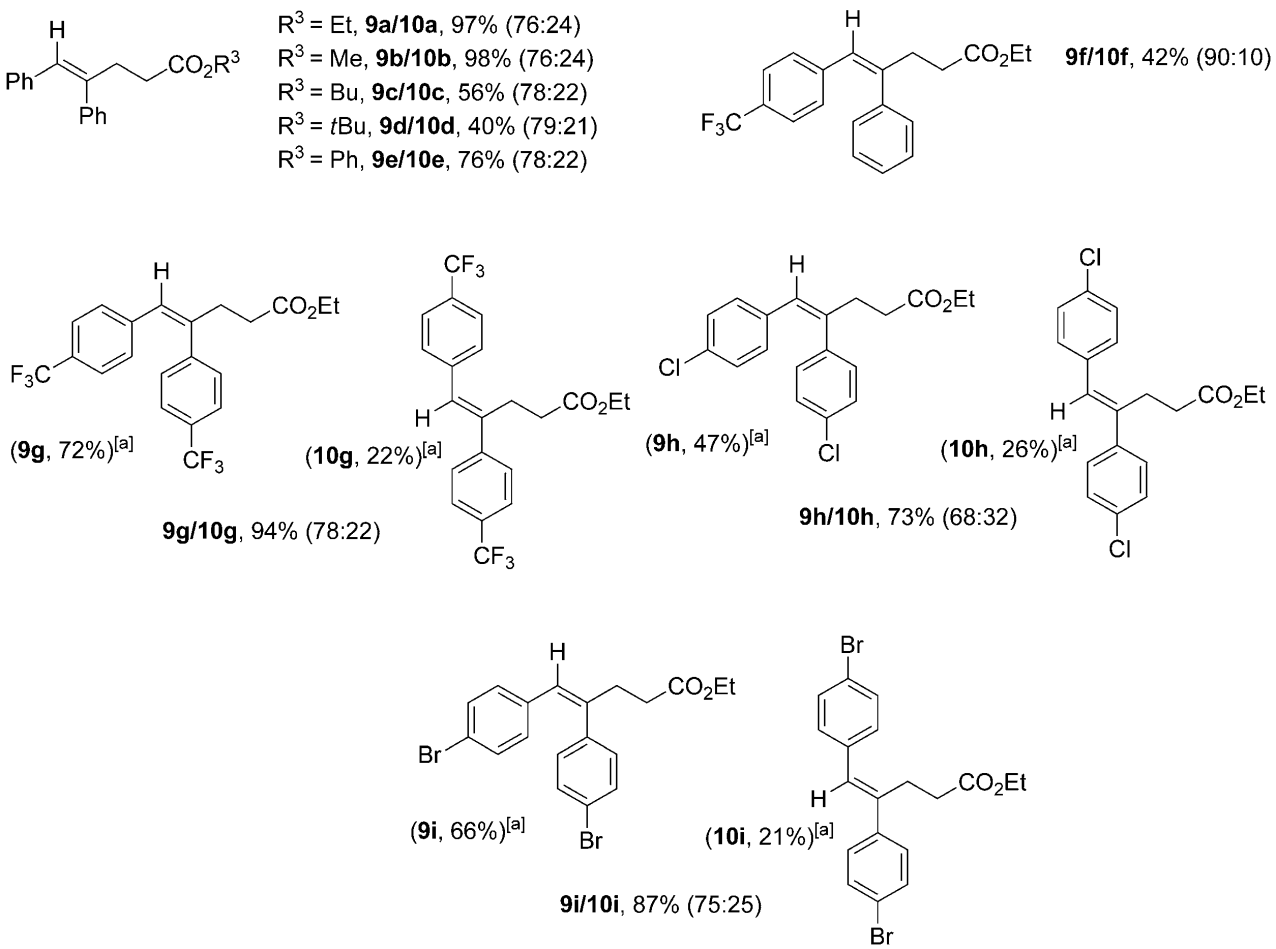

Combined isolated yield. Selectivity was determined by ^1^H NMR analysis of the crude mixture. Structures of major isomer are represented. [a] Isolated yields of **9** and **10**.

To gain insights into this transformation and propose a rational reaction pathway, some control experiments were carried out (Scheme 2). Firstly, to determine the proton source. As it is shown in Scheme 2 a, when the reaction was performed in CD_3_CN, no deuterated products were observed. Nevertheless, when the deuterated HE (**11**) was employed, the corresponding deuterated products were obtained together with the deuterated form of the oxidized HE (Scheme 2 b). Besides, Stern–Volmer quenching experiments reveal significant quenching interactions between the excited state of the photocatalyst and the HE (see Figure S5 in the Supporting Information). This indicates that the HE could act as both proton source and terminal reductant. Additionally, the quantum yield (Φ) was found to be 5 %, which supports that a radical‐chain propagation mechanism is unlikely. Moreover, this is also in agreement with on/off experiments where the catalytic system is activated under irradiation and deactivated in darkness (see Figure S6 in the Supporting Information). Then, to prove that this process involves radicals, the reaction was conducted in the presence of the radical scavenger 2,2,6,6‐tetramethylpiperidine‐1‐oxyl (TEMPO; Scheme 2 c). In this case, the process was completely inhibited, which provides evidence of a possible initial electron transfer.^19^

**Scheme 2:**
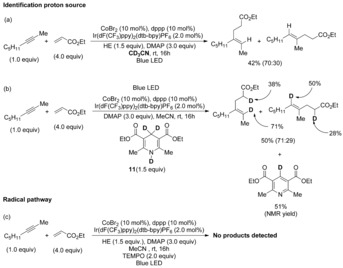
Mechanistic studies.

In light of the experimental data, as well as previous studies in metallophotoredox catalysis and intermolecular reductive ene–yne coupling, a plausible mechanism was proposed in Scheme 3. First, a SET process from the HE to the excited‐state photocatalyst Ir^III^* might occur generating the radical cation **A**. This reduction could be possible given their reported reduction potentials (*E*_1/2_^red^ [Ir^III^/Ir^II^]=−1.37 V vs. SCE and *E*_1/2_^red^ [HE]=−2.3 V vs. SCE).7b, 16 Then, the Co^II^ salt may be reduced in situ to Co^I^ species by the photocatalyst.7b Next, considering that the HE can contribute in both SET and HAT processes,15c a hydrogen atom transfer from the radical cation **A** to Co^I^ could afford the Co^II^−H complex.^20^ Here, it is assumed that DMAP would facilitate the deprotonation of **A** to give **B**.15c The acrylate could undergo migratory insertion in to Co−H giving the five‐membered metallacycle **C**.^21^ At this stage, depending on the nature of the alkyne, two different insertion paths into the Co−C bond could occur. Thus, when using alkyl–alkyl substituted alkynes, intermediate **D** could be generated (Path I), whereas for alkynes bearing one or two aromatic groups intermediate **E** might be formed (Path II). From these intermediates, a subsequent reduction of Co^III^ to Co^II^ by the photocatalyst7b followed by a final protonolysis step would deliver the corresponding products and Co^II^ back into the catalytic cycle.^3^

**Scheme 3:**
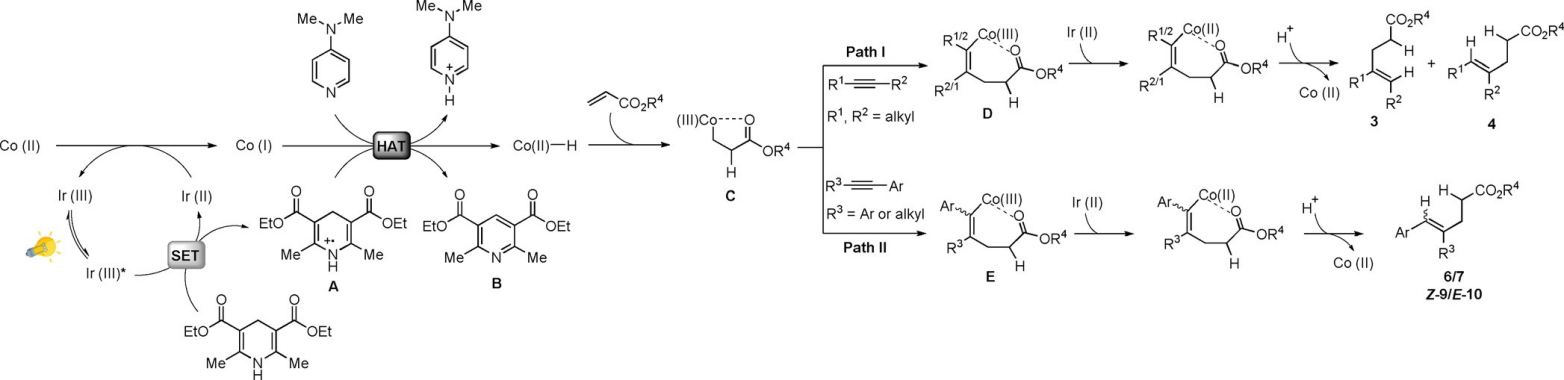
Proposed mechanism for the visible‐light‐driven intermolecular reductive ene–yne coupling by cobalt/iridium dual catalysis.

In summary, we have reported a new method to form C(sp^3^)−C(sp^2^) bonds by visible‐light‐driven intermolecular reductive ene–yne coupling from commercially available and bench‐stable alkynes and alkenes. This approach relies on the ability of the HE to contribute in both SET and HAT processes through the synergistic combination of photoredox and cobalt catalysis. The employment of very mild reaction conditions avoiding the use of metallic reagents is translated into a broad functional‐group tolerance. Besides, the proposed approach is in line with main goals in synthesis, such as atom‐economy and sustainability principles highlighting the potential of this transformation. Further studies to expand the scope, as well as new synthetic applications, are currently underway.

## Conflict of interest

The authors declare no conflict of interest.

## Supporting information

As a service to our authors and readers, this journal provides supporting information supplied by the authors. Such materials are peer reviewed and may be re‐organized for online delivery, but are not copy‐edited or typeset. Technical support issues arising from supporting information (other than missing files) should be addressed to the authors.

Supplementary
