# Supplementary material for: Visible‐Light‐Driven Intermolecular Reductive Ene–Yne Coupling by Iridium/Cobalt Dual Catalysis for C(sp3)−C(sp2) Bond Formation
Source: Chemistry. 2019 Nov 8;25(69):15746–50. doi: 10.1002/chem.201903708 (PMC6916364; doi:10.1002/chem.201903708)

CHEMISTRY
A European Journal

Supporting Information

Visible-Light-Driven Intermolecular Reductive Ene–Yne Coupling by Iridium/Cobalt Dual Catalysis for C(sp^3)–C(sp^2) Bond Formation

María J. González and Bernhard Breit*[a]

chem_201903708_sm_miscellaneous_information.pdf

Table of Contents

Experimental procedures	S3
Optimization of the reaction conditions	S4
Control experiments	S5
Screening of ligands	S6
Scope attempts	S7
General procedure and characterization for alkyl-alkyl disubstituted alkynes	S8
General procedure and characterization for 1-aryl-1-alkyl disubstituted alkynes	S11
General procedure and characterization for diaryl substituted alkynes	S20
Deuteriation labelling experiments	S25
Quantum yield measurement	S27
Stern-Volmer fluorescence quenching studies	S28
On/Off studies	S30
References	S31
Copies of NMR spectra of the products	S32

Experimental Procedures

Working techniques: All reactions were carried out using standard Schlenk techniques in flame dry glassware under Argon (Argon 5.0, Sauerstoffwerk Friedrichschafen) with magnetic stirring. All fotochemical reactions were performed in 10 ml screw-capped tubes and irradiated with a 4.8 W Blue LED strip. High temperature reactions were performed using a hot plate/oil bath apparatus with internal temperature control or a heating mantle.

Reagents: Alkynes **1a-c**, **5a** and **6k** were used as received. The rest of alkynes were prepared according to literature.[1,2] Ethyl acrylate was acquired from Acros Organics and acrylonitrile from Aldrich and used as reveived. DIMAP CsOPiv, pyridine, dimethyl-2,6-pyridinedicarboxylate, 4-phenylpyridine and DMAP (4-dimethylamino pyiridene) were purchased from commercial sources. Et_3N was destilled from KOH. DIPEA was distilled from ninhydrin and then re-distilled from KOH. Hantzsch ester (HE)[3] and the deuterated Hantzsch ester[4] were synthesized according to the reported literature procedures.

Ligands and catalysts: Ligands, cobalt catalysts and ruthenium catalysts were acquired from commercial suppliers and used directly. Dppe and xantphos from Sigma-Aldrich, $CoBr_2$ from FluoroChem, CoI_2 from Sigma-Aldrich, $[Ru(bpz)_2][PF_6]$ from Sigma-Aldrich and $Ru(BPY)_3Cl_2$ from Sigma-Aldrich. $[Ir(dF(CF_3)ppy)_2(dtb\text{-}bpy)PF_6]$ and $[Ir(ppy)_2(dtb\text{-}bpy)PF_6]$ were synthesized according to a reported literature procedure.[5]

Solvents: 1,2-Dichloroethane (DCE) was distilled from CaH_2 under argon atmosphere. Tetrahydrofuran (THF) and Toluene were distilled from potassium under argon atmosphere. Acetonitrile (MeCN) Extra Dry over Molecular Sieve AcroSeal® was purchased from Acros Organics and used as received. N,N-dimethylformamide (DMF) over Molecular Sieve AcroSeal® was purchased from Acros Organics and dimethyl sulfoxide (DMSO) sealed was acquired from Sigma-Aldrich. Deuterium oxide (D_2O) 99,9% D was purchased from Deutero. Distilled water was employed as co-solvent without been degassed. Solvents employed for work-up and column chromatography were purchased in technical grade quality and used without further purification.

Flash Column Chromatography was accomplished using silica gel 60, (0.04-0.063 mn, Machery-Nagel).

Thin Layer Chromatography (TLC) was performed on aluminium-backed plates coated with silica gel (MERCK, 60 F254), which were visualized by UV fluorescence (λmax = 254 nm) and by staining with 1% w/v $KMnO_4$ in 0.5 M aqueous K_2CO_3.

Nuclear Magnetic Resonance (NMR): routine 1H-NMR analyses were acquired on a *BRUKER Avance 300 spectrometer* (1H-NMR: 300.13 MHz). For high field experiments a *BRUKER Avance 400 spectrometer* (400.41 MHz and 100.69 MHz for ^1H and ^{13}C respectively) or a *BRUKER Avance 500 spectrometer* (500.22 MHz and 125.79 MHz for ^1H and ^{13}C) were used by the analytical department at the Institut für Organische Chemie und Biochemie, Universität Freiburg. Chemical shifts (δ) are given in parts per million (ppm) and measured relative to residual solvent $CHCl_3$ (^1H: 7.27 ppm; ^{13}C: 77.16 ppm) and coupling constants (J) in hertz (Hz). Unequivocal ^1H and ^{13}C assignments were made with the aid of two-dimensional HH-COSY, HSQC, HMBC, TOCSY and NOESY experiments. Data for ^1H NMR are described as following: ^1H-NMR: chemical shift (multiplicity, coupling constant (*J*), number of protons); ^{13}C-NMR: chemical shift; ^{19}F-NMR: Chemical shift. The multiplicity is abbreviated as: s = singlet, d = doublet, t = triplet, q = quartet, qt = quartet of triplet, sp = septuplet and m = multiplet.

High resolution mass spectra (HR-MS): Mass spectra were recorded by the analytic department at the Institut für Organische Chemie und Biochemie, Universität Freiburg on a THERMO SCIENTIFIC Advantage and a THERMO SCIENTIFIC Exactive instrument equipped with an APCI or ESI source in the positive-ion mode

Melting point (mp): Melting points were measured with a Bibby Scientific Limited Stuart smp10.

Results and Discussion

Optimization of the reaction conditions

Commercially available 2-octyne and ethyl acrylate were selected as model substrates the optimization. The results are summarized in Table S1. Firstly, DIPEA was tested as organic reductant with CsOPiv and pyridine as bases without good results (Table S1, entries 1-3). However, when the HE was employed together with DIMAP **3a/4a** were obtained in a 73% yield (Table S1, entry 16). Other solvents than MeCN did not improve the previous result.

Table S1. Optimization of organic reductant, base and solvent.[a]

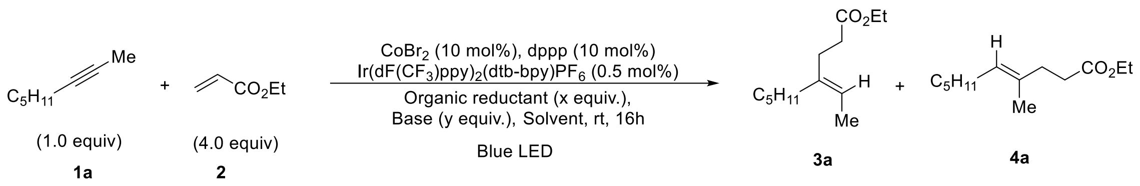

Entry	Reductant	X	Base	Y	solvent	Yield (%)[b,c]
**1**	DIPEA	3.0	CsOpiv	0.5	MeCN	13
**2**	DIPEA	2.0	CsOpiv	1.0	MeCN:H_2O (40:1)	9
**3**	DIPEA	3.0	Pyridine	0.5	MeCN	Complex mixture
**4**	HE	1.5	Pyridine	3.0	MeCN:H_2O (40:1)	24
**5**	HE	1.5	Pyridine	2.0	MeCN:H_2O (40:1)	20
**6**	HE	1.5	Pyridine	3.0	MeCN	5
**7**	HE	1.5	Pyridine	6.0	MeCN	46
**8**	HE	1.5	Et_3N	3.0	MeCN	No reaction
**9**	HE	1.5	CsOpiv	0.5	MeCN:H_2O (40:1)	9
**10**	HE	1.5	Dimethyl-2,6-pyridinedicarboxylate	2.0	MeCN:H_2O (40:1)	10
**11**	HE	1.5	4-phenylpyridine	2.0	MeCN	27
**12**	HE	1.5	DMAP	2.0	MeCN:H_2O (40:1)	32
**13**	HE	1.5	DMAP	3.0	MeCN:H_2O (40:1)	66
**14**	HE	1.5	DMAP	6.0	MeCN:H_2O (40:1)	51
**15**	HE	1.5	DMAP	3.0	MeCN:EtOH (40:1)	26
**16**	HE	1.5	DMAP	3.0	MeCN	73
**17**	HE	1.5	DMAP	3.0	DMF	73
**18**	HE	1.5	DMAP	3.0	Acetone	45

[a] Optimizations were performed on a 0.2 mmol scale using **1a** (1.0 equiv.), **2** (4.0 equiv.), $CoBr_2$ (10 mol%) and dppp (10 mol%) over a period of 16h under irradiation with a 4.8 W Blue LED strip. [b]Isolated yield. [c]**3a/4a** were always obtained in a (70:30) selectivity determined by ^{1}H-NMR analysis of the crude mixture.

A screening on the photocatalyst (PC) was then conducted (Table S2) showing that an increment of the PC load to 2.0 mol% affords **3a/4a** in an 80% (entry 5). $Ir(ppy)_2(dtb\text{-}bpy)PF_6$ was also tested giving a lower yield (entry 6). Other commonly employed ruthenium photocatalyst such as $[Ru(bpz)_2][PF_6]$ and $[Ru(BPY)_3]$ did not show results (entries 7-8).

Table S2. Optimization of the photocatalyst.[a]

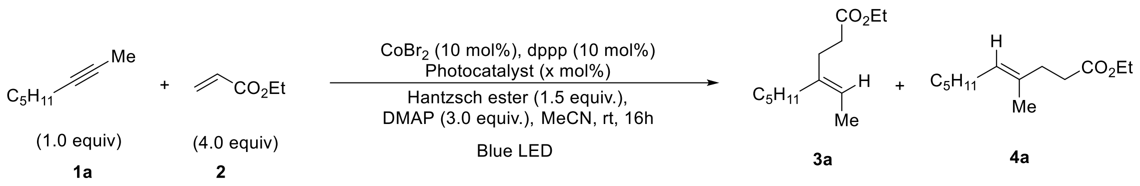

Entry	photocatalyst	x	Yield (%)[b,c]
**1**	$Ir(dF(CF_3)ppy)_2(dtb\text{-}bpy)PF_6$	0.5	73
**2**	$Ir(dF(CF_3)ppy)_2(dtb\text{-}bpy)PF_6$	1.0	75
**3**	$Ir(dF(CF_3)ppy)_2(dtb\text{-}bpy)PF_6$	1.5	77
**4**	$Ir(dF(CF_3)ppy)_2(dtb\text{-}bpy)PF_6$	2.0	80
**5**	$Ir(dF(CF_3)ppy)_2(dtb\text{-}bpy)PF_6$	3.0	78
**6**	$Ir(ppy)_2(dtb\text{-}bpy)PF_6$	2.0	70
**7**[d,]	$[Ru(bpz)_2][PF_6]$	0.5	nr
**8**[d,]	$Ru(BPY)_3Cl_2$	0.5	nr

[a] Optimizations were performed on a 0.2 mmol scale using **1a** (1.0 equiv.), **2** (4.0 equiv.), $CoBr_2$ (10 mol%), dppp (10 mol%), HE (1.5 equiv) and DMAP (3.0 equiv.) in MeCN over a period of 16h under irradiation with a 4.8 W Blue LED strip. [b]Isolated yield. [c]**3a/4a** were always obtained in a (70:30) selectivity determined by ^{1}H-NMR analysis of the crude mixture. [d] CsOPiv as base. nr = No reaction.

Control experiments

Then, tests reactions in the absence of $CoBr_2$, PC, HE, DMAP and Blue LED were performed proving that all the additives are required for the transformation (Table S3).

Table S3. Control experiments.[a]

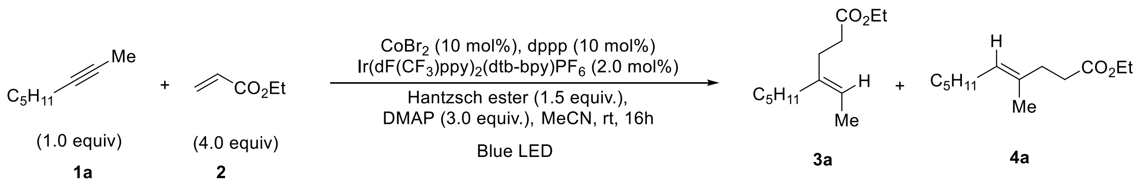

Entry	$CoBr_2$	Photocatalyst	Hantzsch ester	DMAP	Blue LED	
**1**	x	+	+	+	+	nr
**2**	+	x	+	+	+	nr
**3**	+	+	x	+	+	nr
**4**	+	+	+	x	+	nr
**5**	+	+	+	+	x	nr
**6**	+	+	+	+	+	reaction

[a] Optimizations were performed on a 0.2 mmol scale using **1a** (1.0 equiv.), **2** (4.0 equiv.), $CoBr_2$ (10 mol%), dppp (10 mol%), $Ir(dF(CF_3)ppy)_2(dtb\text{-}bpy)PF_6$ (2.0 mol%), HE (1.5 equiv) and DMAP (3.0 equiv.) in MeCN over a period of 16h under irradiation with a 4.8 W Blue LED strip. nr = No reaction.

Screening of ligands

In order to improve the selectivity, several ligands phosphine ligands were tested. We observed that for 2-octyne (**1a**) the previous result with dppp was not improved (Table S4) whereas for aryl substituted alkynes xantphos proved to be the best ligand (Table S5)

Table S4. Screening of ligands: alkyl-alkyl disubstituted alkynes.

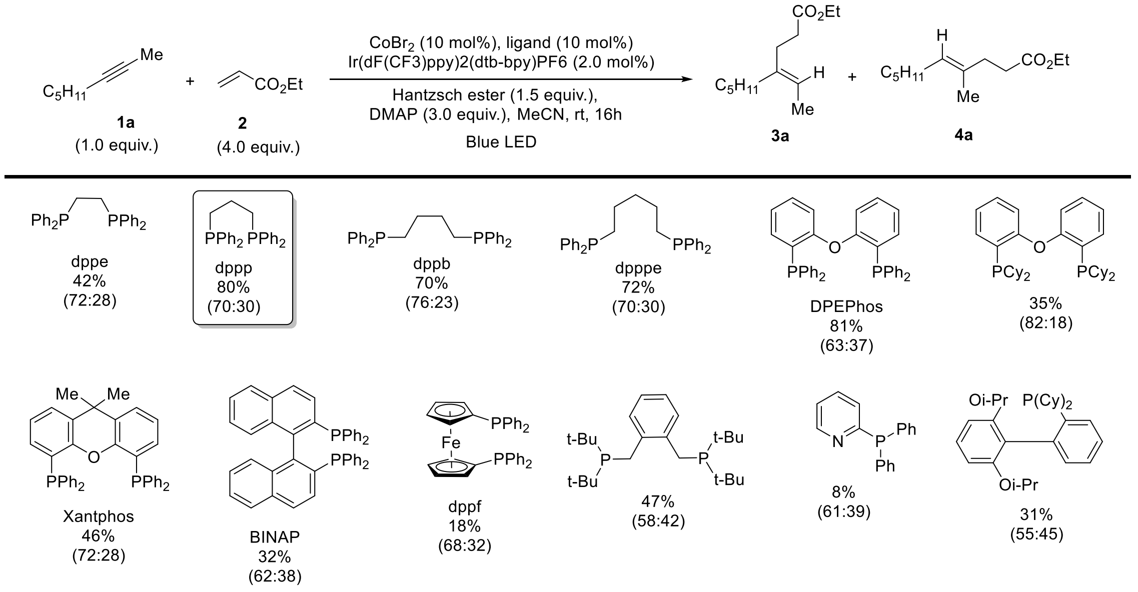

Table S5. Screening of ligands: aryl substituted alkynes.

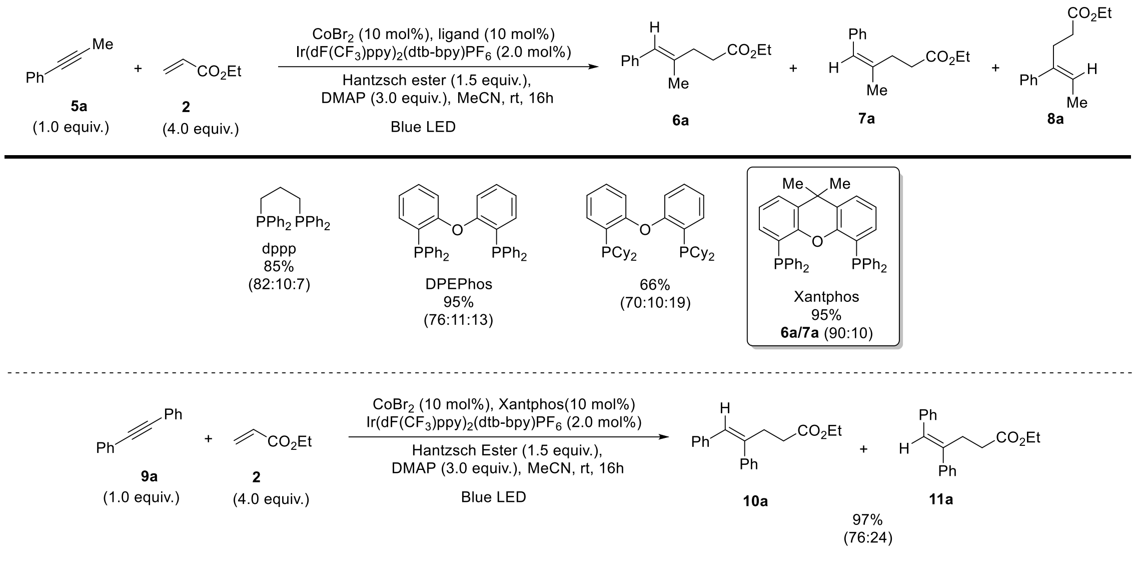

Scope attempts

Other alkenes **Table S6** and alkynes were tested **Table S7** without success.

Table S6. Unreactive alkenes.

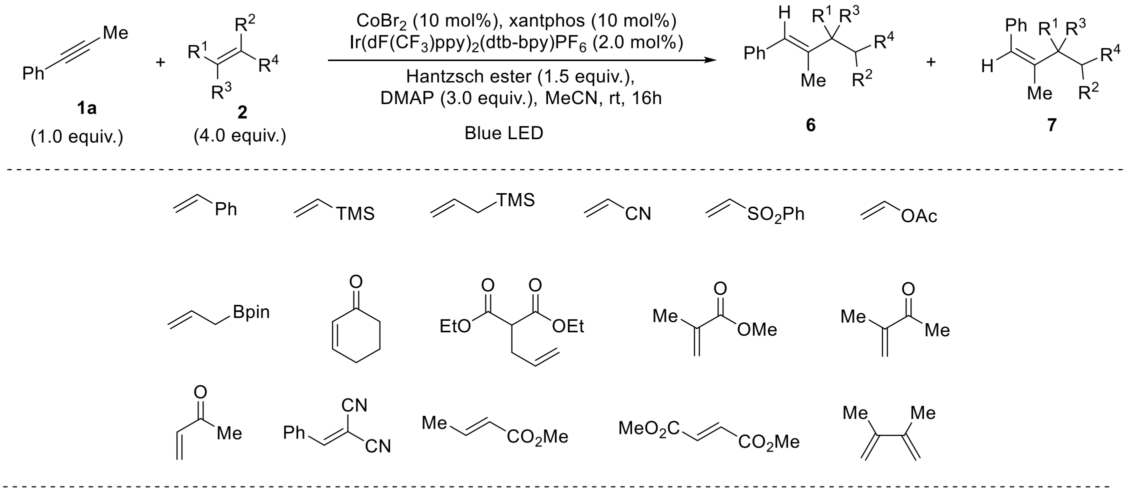

Table S7. Unreactive alkynes.

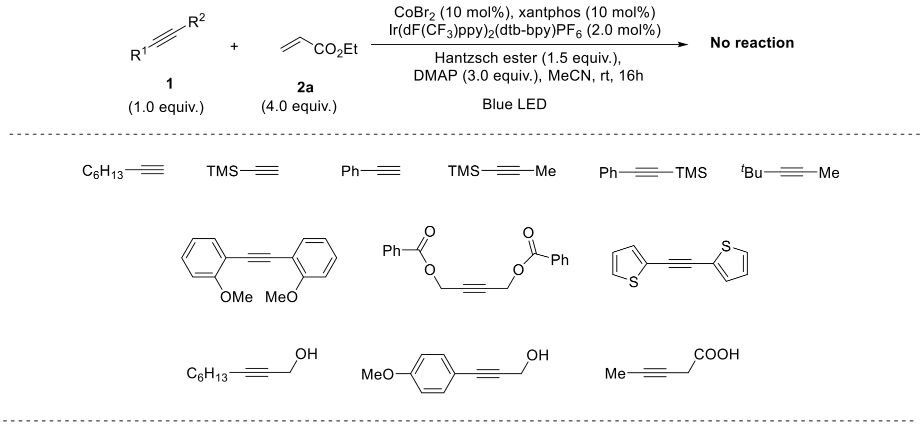

General procedure and characterization for alkyl-alkyl disubstituted alkynes.

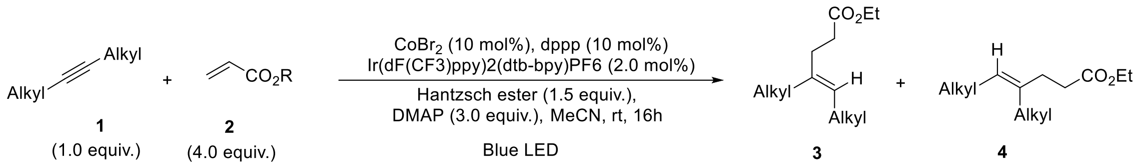

A flame-dried Schlenk tube was charged with $CoBr_2$ (4.37 mg, 0.02 mmol, 10 mol%), dppp (8.25 mg, 0.02 mmol, 10 mol%), Ir(dF(CF_3)ppy)$_2$(dtb-bpy)PF_6 (4.48 mg, 0.02 mmol, 2.0 mol%), Hantzsch ester (75.99 mg, 0.30 mmol, 1.5 equiv.) and DMAP (73.30mg, 0.6 mmol, 3.0 equiv). The tube was evacuated and backfilled with argon for three times. 2 ml of MeCN was added and the mixture was stirred for 10 min at room temperature. Then, the corresponding alkyne (0.2 mmol, 1.0 equiv.) and the acrylate **2** (4.0 equiv.) were added. The resulting mixture was irradiated with a 4.8 W Blue LED strip and stirred at room temperature for 16h. The crude was filtered through a short pad of silica (3 cm) and washed with DCM, dried under vacuum and analysed by ^{1}H-NMR. The resulting residue was purified by flash chromatography (n-pentane/ethyl acetate). ^{1}H-NMR, ^{13}C-NMR spectra and HR-MS were recorded from the mixture; only unambiguous NMR signals were assigned).

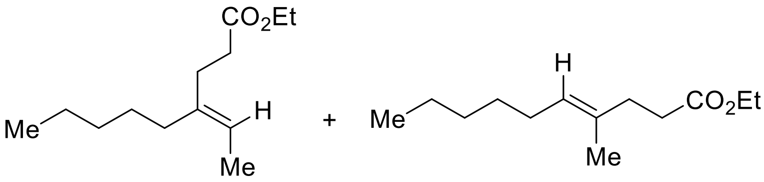

3a **4a**

Ethyl (E)-4-ethylidenenonanoate (3a) and ethyl (E)-4-methyldec-4-enoate (4a): The representative procedure was followed using 2-octyne **1a** (30 µl, 0.20 mmol, 1.0 equiv.) and ethyl acrylate **2a** (82 µl, 0.80 mmol, 4.0 equiv.). After 16 h, flash chromatography (neutral Al_2O_3, n-pentane:EtOAc = 40:1, R_f = 0.61; n-pentane:EtOAc = 10:1) afforded an inseparable mixture of **3a/4a** (34.11 mg, 80%, **3a:4a** = 70:30) as a colourless oil.

^{1}H-NMR (500 MHz, $CDCl_3$): 5.21 (q, *J* = 6.75 Hz, 1H, **3a**), 5.18-5.14 (m, 1H, **4a**), 4.12 (q, *J* = 7.1 Hz, 2H, **3a**), 4.11 (q, *J* = 7.15, 2H, **4a**), 2.41-2.36 (m, **3a/4a**), 2.31-2.27 (m, **3a/4a**), 2.02-1.99 (m, 2H, **3a**), 1.97-1.93 (m, 2H, **4a**), 1.60 (d, *J* = 0.85 Hz, 1H, **4a**), 1.56 (d, *J* = 6.75, 3H, **3a**), 1.39-1.23 (m, 18H, **3a/4a**), 0.9-0.86 (m, 6H, **3a/4a**) **^{13}C-NMR** (125 MHz, $CDCl_3$): 173.71 (C, **3a**), 173.63 (C, **4a**), 138.78 (C, **3a**), 133.16 (C, **4a**), 125.75 (CH, **4a**), 119.17 (CH, **3a**), 60.29 (CH_2, **3a/4a**), 34.82 (CH_2, **4a**), 33.46 (CH_2, **3a**), 33.40 (CH_2, **4a**), 32.03 (CH_2, **3a**), 31.96 (CH_2, **3a**), 31.58 (CH_2, **4a**), 29.81 (CH_2, **3a**), 29.48 (CH_2, **4a**), 27.93 (CH_2, **4a**), 27.89 (CH_2, **3a**), 22.68 (CH_2, **3a/4a**), 15.95 (CH_3, **4a**), 14.33 (CH_3, **3a/4a**), 14.15 (CH_3, **3a/4a**), 13.27 (CH_3, **3a**). (Four C signals could not be located likely due to overlapping).

HR-MS (EI) calc. for $[C_{13}H_{25}O_2]^+$ 213.1849, found 212.1776.

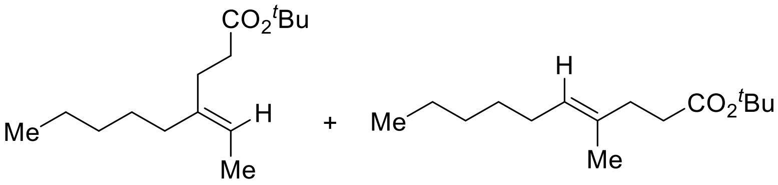

3b **4b**

tert-butyl (E)-4-ethylidenenonanoate (3b) and tert-butyl (E)-4-methyldec-4-enoate (4b): The representative procedure was followed using 2-octyne **1a** (30 µl, 0.20 mmol, 1.0 equiv.) and *tert*-butyl acrylate **2b** (117 µl, 0.80 mmol, 4.0 equiv.). After 16 h, flash chromatography (neutral Al_2O_3, n-pentane:EtOAc = 100:1, R_f = 0.67; n-pentane:EtOAc = 10:1) afforded an inseparable mixture of **3b/4b** (26.25 mg, 55%, **3b:4b** = 68:32) as a colourless oil.

[1]**H-NMR** (500 MHz, $CDCl_3$): 5.21 (q, *J* = 6.75 Hz, 1H, **3b**), 5.16-5.13 (m, 1H, **4b**), 2.38-2.28 (m, 2H), 2.26-2.23 (m, 2H), 2.02-2.00 (m, 2H, **3b**), 1.98-1.94 (m, 2H, **4b**), 1.60-1.59 (m, 2H, **4b**), 1.56 (d, *J* = 6.75, 3H, **3b**), 1.43-1.42 (m, 18H), 0.9-0.86 (m, 6H)) [13]**C-NMR** (125 MHz, $CDCl_3$): 173.08 (C, **3b**), 173.00 (C, **4b**), 138.91 (C, **3b**), 133.35 (C, **4b**), 125.50 (CH, **4b**), 119.02 (CH, **3b**), 80.06 (CH_2), 34.93 (CH_2, **4b**), 33.60 (CH_2, **3b**), 34.51 (CH_2, **4b**), 32.23 (CH_2, **3b**), 31.98 (CH_2, **3b**), 31.62 (CH_2, **4b**), 29.78 (CH_2, **3b**), 29.53 (CH_2, **4b**), 28.19 (CH_3), 27.65 (CH_2, **4b**), 27.90 (CH_2, **3b**), 22.69 (CH_2), 15.98 (CH_3, **4b**), 14.15 (CH_3), 13.24 (CH_3, **3b**). (Some signals could not be located likely due to overlapping).

HRMS (pos. ESI): calculated for $C_{15}H_{29}O_2$ $[M+H]^+$ 241.2155, found 240.3870.

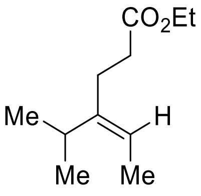

3c

ethyl (Z)-4-isopropylhex-4-enoate (3b): The representative procedure was followed using 4-methylpent-2-yne **1b** (23 µl, 0.20 mmol, 1.0 equiv.) and ethyl acrylate **2a** (82 µl, 0.80 mmol, 6.0 equiv.). After 16 h, flash chromatography (neutral Al_2O_3, n-pentane:EtOAc = 40:1, R_f = 0.65; n-pentane:EtOAc = 10:1) afforded **3b** (12.69 mg, 34%) as a colourless oil.

[1]**H-NMR** (500 MHz, $CDCl_3$): 5.10 (qt, J = 8.5, 1.7 Hz, 1H), 4.13 (q, J = 8.9 Hz, 2H), 2.86 (sp, J = 8.7 Hz, 1H), 2.43-2.39 (m, 2H), 2.28-2.23 (m, 2H), 1.59 (dt, J = 8.5, 1.8 Hz, 3H), 1.24 (t, J = 8.9 Hz, 3H), 0.99 (d, J = 8.75, 6H). [13]**C-NMR** (125 MHz, $CDCl_3$): 173.77 (C), 143.52 (C), 117.12 (CH), 60.27 (CH_2), 33.93 (CH_2), 28.59 (CH), 26.21 (CH_2), 20.88 (CH_3 x 2), 14.34 (CH_3), 12.80 (CH_3).

HRMS (pos. ESI): calculated for $C_{11}H_{21}O_2$ $[M+H]^+$ 185.1497, found 184.2790.

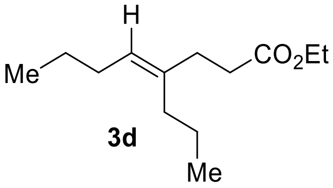

3d

Ethyl (E)-4-butylnon-4-enoate (3d): The representative procedure was followed using 4-octyne **1c** (30 µl, 0.20 mmol, 1.0 equiv.) and ethyl acrylate **2a** (82 µl, 0.80 mmol, 6.0 equiv.). After 16 h, flash chromatography (neutral Al_2O_3, n-pentane:EtOAc = 40:1, R_f = 0.53; n-pentane:EtOAc = 10:1) afforded **3c** (28.21 mg, 66%) as a colourless oil.

[1]**H-NMR** (500 MHz, $CDCl_3$): 5.14 (t, J = 7.15 Hz, 1H), 4.11 (t, J = 7.15 Hz, 1H), 2.40-2.37 (m, 2H), 2.31-2.27 (m, 2H), 2.00-1.93 (m, 4H), 1.42-1.29 (m, 4H), 1.24 (t, J = 7.15 Hz, 3H), 0.90-0.86 (m, 6H). [13]**C-NMR** (125 MHz, $CDCl_3$): 173.67 (C), 137.64 (C), 125.72 (CH), 60.28 (CH_2), 33.57 (CH_2), 32.26 (CH_2), 31.95 (CH_2), 29.86 (CH_2), 23.18 (CH2), 21.61 (CH_2), 14.33 (CH_3), 14.17 (CH_3), 13.88 (CH_3).

HRMS (pos. ESI): calculated for $C_{13}H_{23}O_2$ $[M+H]^+$ 211.1693, found 212.3330.

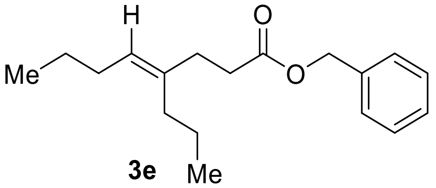

3e

Benzyl (E)-4-propyloct-4-enoate (3e): The representative procedure was followed using 4-octyne **1c** (30 µl, 0.20 mmol, 1.0 equiv.) and benzyl acrylate **2c** (122 µl, 0.80 mmol, 4.0 equiv.). After 16 h, flash chromatography (neutral Al_2O_3, n-pentane:EtOAc = 40:1, R_f = 0.45; n-pentane:EtOAc = 10:1) afforded **3e** (22.22 mg, 43%) as a colourless oil.

^{1}H-NMR (500 MHz, $CDCl_3$): 7.38-7.31 (m, 5H), 5.14 (t, J = 7.15 Hz, 1H), 5.11 (s, 2H), 2.48-2.45 (m, 2H), 2.34-2.31 (m, 2H), 2.00-1.93 (m, 4H), 1.44-1.26 (m, 4H), 0.89-0.86 (m, 6H). **^{13}C-NMR** (125 MHz, $CDCl_3$): 173.47 (C), 137.53 (C), 136.20 (C), 128.62 (CH), 128.29 (CH), 128.24 (CH), 125.85 (CH), 66.21 (CH_2), 33.53 (CH_2), 32.27 (CH_2), 31.89 (CH_2), 23.17 (CH_2), 21.61 (CH2), 14.78 (CH_3), 13.90 (CH_3).

HRMS (pos. ESI): calculated for $C_{18}H_{27}O_2$ $[M+H]^+$ 275.1966, found 274.4040.

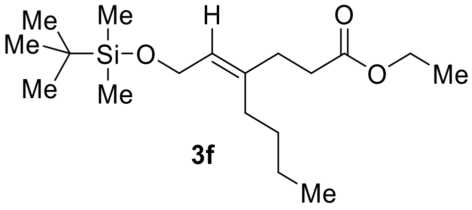

3f

ethyl (E)-4-(2-((tert-butyldimethylsilyl)oxy)ethylidene)octanoate (3f): The representative procedure was followed using tert-butyl(hept-2-yn-1-yloxy)dimethylsilane **1d** (45, 0.20 mmol, 1.0 equiv.) and ethyl acrylate **2a** (82 µl, 0.80 mmol, 6.0 equiv.). After 16 h, flash chromatography (SiO_2, n-pentane:EtOAc = 40:1, R_f = 0.50; n-pentane:EtOAc = 10:1) afforded **3f** (41.79 mg, 65%) as a colourless oil.

^{1}H-NMR (500 MHz, $CDCl_3$): 5.28 (t, J = 6.3 Hz, 1H), 4.18 (d, J = 6.3 Hz, 2H), 4.12 (q, J = 7.15 Hz, 4H), 2.43-2.39 (m, 2H), 2.43-2.40 (m, 2H), 2.34-2.31 (m, 2H), 2.02-1.99 (m, 2H), 1.36-1.28 (m, 4H), 1.25 (t, J = 7.15 Hz, 3H), 0.91-0.88 (m, 12H), 0.06 (s, 6H). **^{13}C-NMR** (125 MHz, $CDCl_3$): 173.44 (C), 139.77 (C), 125.15 (CH), 60.39 (CH_2), 59.99 (CH_2), 33.04 (CH_2), 31.67 (CH_2), 30.82 (CH_2), 30.59 (CH_2), 26.08 (CH_3 x 3), 22.83 (CH_2), 14.33 (CH_3), 14.04 (CH_3), -4.099 (CH_3 x 3).

HRMS (pos. ESI): calculated for $C_{11}H_{21}O_2$ $[M+H]^+$ 329.2467, found 328.5680.

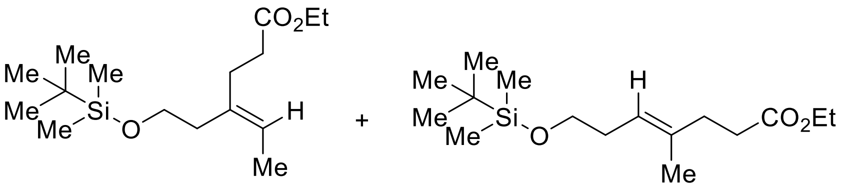

3g **4g**

ethyl (Z)-4-(2-((tert-butyldimethylsilyl)oxy)ethyl)hex-4-enoate (3g) and ethyl (E)-7-((tert-butyldimethylsilyl)oxy)-4-methylhept-4-enoate (4g): The representative procedure was followed using tert-butyldimethyl(pent-3-yn-1-yloxy)silane **1e** (42 mg, 0.20 mmol, 1.0 equiv.) and ethyl acrylate **2a** (82 µl, 0.80 mmol, 4.0 equiv.). After 16 h, flash chromatography (SiO_2, n-pentane:EtOAc = 40:1, R_f = 0.50; n-pentane:EtOAc = 10:1) afforded an inseparable mixture of **3g/4g** (51.79 mg, 81%, **3g:4g** = 58:42) as a colourless oil.

^{1}H-NMR (500 MHz, $CDCl_3$): 5.30 (q, J = 6.8 Hz, 1H, **3g**), 5.19 – 5.11 (m, 1H, **4g**), 4.11 (q, J = 7.15 Hz, 2H, **4g**), 4.10 (q, J = 7.15 Hz, 2H, **3g**), 3.61 (t , J = 7.15 Hz, 2H, **3g**), 3.56 (t , J = 7.15 Hz, 2H, **4g**), 2.42 – 2.35 (m, **3g/4g**), 2.33 – 2.23 (m, **3g/4g**), 2.29 – 2.26 (m, **3g/4g**), 2.23 – 2.19 (m, **3g/4g**), 1.62 (d, J = 0.6 Hz, 3H, **4g**), 1.58 (d, J = 6.8 Hz, 3H, **3g**), 1.24 (t, J = 7.15 Hz, 3H, **4g**), 1.23 (t, J = 7.15 Hz, 3H, **3g**), 0.90 – 0.82 (m, **3g/4g**), 0.05 (s, 6H), 0.04 (s, 6H) **^{13}C-NMR** (125 MHz, $CDCl_3$): 173.53 (C), 135.44 (C, **4g**), 135.32 (C, **3g**), 121.34 (CH), 63.04 (CH_2, **4g**), 61.88

(CH$_2$, **3g**), 60.34 (CH$_2$, **4g**), 60.32 (CH$_2$, **3g**), 34.84 (CH$_2$), 33.79 (CH$_2$), 33.47 (CH$_2$), 33.31 (CH$_2$), 32.78 (CH$_2$), 31.93 (CH$_2$), 26.07 (CH$_3$), 16.18 (CH$_3$, **4g**), 14.37 (CH$_3$), 13.51 (CH$_3$, **3g**), -5.13 (CH$_3$, **4g**), -5.19 (CH$_3$, **3g**).

HRMS (pos. APCI): calculated for $C_{16}H_{33}O_3Si$ $[M+H]^+$ 301.2193, found 300.5140.

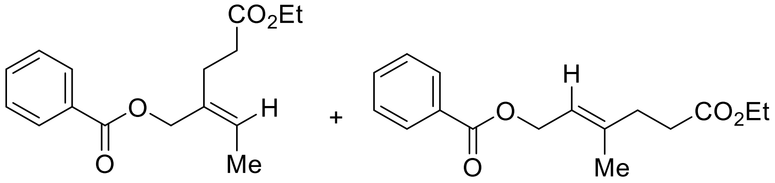

3h **4h**

(Z)-5-ethoxy-2-ethylidene-5-oxopentyl benzoate (3h) and (E)-6-ethoxy-3-methyl-6-oxohex-2-en-1-yl benzoate (4h): The representative procedure was followed using but-2-yn-1-yl benzoate **1f** (36 mg, 0.20 mmol, 1.0 equiv.) and ethyl acrylate **2a** (82 µl, 0.80 mmol, 4.0 equiv.). After 16 h, flash chromatography (SiO$_2$, n-pentane:EtOAc = 40:1, R_f = 0.32; n-pentane:EtOAc = 10:1) afforded an inseparable mixture of **3f/4f** (29.58 mg, 81%, **3h:4h** = 34:66) as a colourless oil.

^{1}H-NMR (500 MHz, CDCl$_3$): 8.05-8.025 (m, **3h/4h**), 7.57-7.53 (m, **3h/4h**), 7.45-7.41 (m, **3h/4h**), 5.59 (q, J = 6.95 Hz, 1H, **3h**), 5.51-5.47 (m, 1H, **4h**), 4.88 (s, 2H, **3h**), 4.83 (dd , J = 7, 0.6 Hz, 2H, **4h**), 4.14-4.09 (m, 4H, **3h/4h**), 2.49 (s, **3h/4h**), 2.47-2.44 (m, **3h/4h**), 2.41-2.38 (m, **3h/4h**), 1.78 (s, 3H, **4h**), 1.74 (d, J = 6.95 Hz, 3H, **3h**), 1.25-1.21 (m, **3h/4h**). **^{13}C-NMR** (125 MHz, CDCl$_3$): 173.16 (C, **3h**), 173.10 (C, **4h**), 140.56 (C), 133.08 (CH$_2$, **3h**), 132.91 (CH, **4h**), 130.48 (C, **4h**), 130.31 (C, **3h**), 129.69 (CH, **3h**), 129.65 (CH, **4h**), 129.45 (CH, **4h**), 128.45 (CH, **3h**), 128.38 (CH, **4h**), 126.33 (CH, **3h**), 119.28 (CH, **4h**), 62.13 (CH$_2$, **3h**), 61.70 (CH$_2$, **4h**), 60.46 (CH$_2$, **4h**), 60.40 (CH$_2$, **3h**), 34.48 (CH$_2$, **4h**), 33.30 (CH$_2$, **3h**), 32.78 (CH$_2$, **4h**), 30.71 (CH$_2$, **3h**), 16.60 (CH$_3$, **4h**), 14.30 (CH$_3$, **3h/4h**), 13.50 (CH$_3$, **3h**).

HRMS (pos. APCI): calculated for $C_{16}H_{24}O_4N$ $[M+NH_4]^+$ 294.1700, found 276.3320.

General procedure and characterization for 1-aryl-1-propynes.

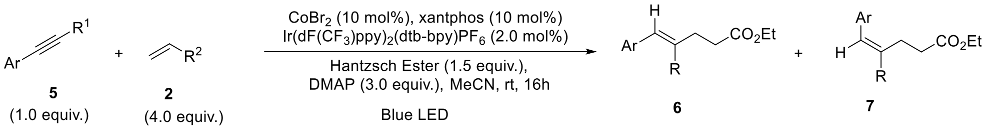

A flame-dried Schlenk tube was charged with CoBr$_2$ (4.37 mg, 0.02 mmol, 10 mol%), xantphos (8.25 mg, 0.02 mmol, 10 mol%), Ir(dF(CF$_3$)ppy)$_2$(dtb-bpy)PF$_6$ (4.48 mg, 0.02 mmol, 2.0 mol%), Hantzsch ester (75.99 mg, 0.30 mmol, 1.5 equiv.) and DMAP (73.30 mg, 0.6 mmol, 3.0 equiv). The tube was evacuated and backfilled with argon for three times. 2 ml of MeCN was added and the mixture was stirred for 10 min at room temperature. Then, the corresponding alkyne (0.2 mmol, 1.0 equiv.) and the acrylate **2** (4.0 equiv.) were added. The resulting mixture was irradiated with a 4.8 W Blue LED strip and stirred at room temperature for 16h. The crude was filtered through a short pad of silica (3 cm) and washed with DCM, dried under vacuum and analysed by ^{1}H-NMR. The resulting residue was purified by flash chromatography (n-pentane/ethyl acetate). ^{1}H-NMR, ^{13}C-NMR spectra and HR-MS were recorded from the mixture; only unambiguous NMR signals were assigned).

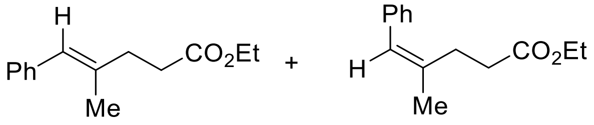

6a **7a**

Ethyl (*E*)-4-methyl-5-phenylpent-4-enoate (6a), ethyl (*Z*)-4-methyl-5-phenylpent-4-enoateand ethyl (7a): The representative procedure was followed using 1-phenyl-1-propyne **5a** (25 µl, 0.20 mmol, 1.0 equiv.) and ethyl acrylate **2** (82 µl, 0.80 mmol, 6.0 equiv.). After 16 h, flash chromatography (SiO_2, n-pentane:EtOAc = 40:1, R_f = 0.43; n-pentane:EtOAc = 10:1) afforded an inseparable mixture of **6a/7a** (42.48 mg, 97%, **6a:7a** = 90:10) as a colourless oil.

^{1}H-NMR (300 MHz, $CDCl_3$): 7.26-7.20 (m, **6a/7a**), 7.15-7.08 (m, **6a/7a**), 6.26 (s, 1H, **7a**), 6.23 (s, 1H, **6a**), 4.11-3.99 (m, **6a/7a**), 2.48-2.40 (m, 8H, **6a/7a**), 1.81 (d, *J* = 1.5 Hz, 3H, **7a**), 1.79 (d, *J* = 1.38 Hz, 1H, **6a**), 1.21-1.13 (m, **6a/7a**). **^{13}C-NMR** (125 MHz, $CDCl_3$): 173.28 (C, **6a**), 138.30 (C, **6a**), 137.18 (C, **6a**), 128.91 (CH, **6a**), 128.61 (CH, **7a**), 118.28 (CH, **7a**), 128.13 (CH, **6a**), 126.99 (CH, **7a**), 126.25 (CH, **7a**), 126.16 (CH, **6a**), 125.75 (CH, **7a**), 60.45 (CH_2, **6a**), 35.74 (CH_2, **6a**), 33.31 (CH_2, **6a**), 33.01 (CH_2, **7a**), 27.95 (CH_2, **7a**), 23.69 (CH_2, **7a**), 17.73 (CH_3, **6a**), 14.37 (CH_3, **6a**), 14.27 (CH_3, **7a**). (Four C signals could not be located likely due to overlapping).

HR-MS (EI) calc. for $[C_{14}H_{18}O_2]^+$ 219.1380, found 218.1307.

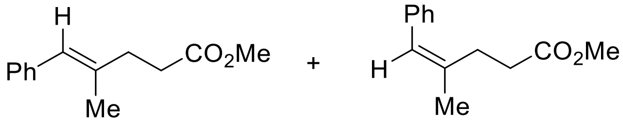

6b **7b**

Methyl (E)-4-methyl-5-phenylpent-4-enoate (6b), methyl (Z)-4-methyl-5-phenylpent-4-enoate (7b): The representative procedure was followed using 1-phenyl-1-propyne **5a** (25 µl, 0.20 mmol, 1.0 equiv.) and methyl acrylate **2d** (72 µl, 0.80 mmol, 4.0 equiv.). After 16 h, flash chromatography (SiO_2, n-pentane:EtOAc = 100:1, R_f = 0.40; n-pentane:EtOAc = 10:1) afforded an inseparable mixture of **6b/7b** (37.44 mg, 92%, **6b:7b** = 83:17) as a colourless oil.

^{1}H-NMR (400 MHz, $CDCl_3$): 7.33-7.29 (m, **6b/7b**), 7.23-7.17 (m, **6b/7b**), 6.33 (s, 1H, **7b**), 6.30 (s, 1H, **6b**), 6.69 (s, 3H, **6b**), 6.65 (s, 3H, **7b**), 2.58-2.47 (m, **6b/7b**), 1.88 (d, *J* = 1.48 Hz, 3H, **7b**), 1.87 (d, *J* = 1.30 Hz, 1H, **6b**). **^{13}C-NMR** (100 MHz, $CDCl_3$): 173.72 (C, **6b**), 138.27 (C, **6b**), 137.08 (C, **6b**), 128.92 (CH, **6b**), 128.59 (CH, **7b**), 128.29 (CH, **7b**), 128.13 (CH, **6b**), 127.08 (CH, **7b**), 126.28 (CH, **7b**), 126.19 (CH, **6b**), 125.79 (CH, **6b**), 51.6692 (CH_3, **6b**), 35.70 (CH_2, **6b**), 33.07 (CH_2, **6b**), 32.74 (CH_2, **7b**), 27.93 (CH_2, **7b**), 23.66 (CH_3, **7b**), 17.73 (CH_3, **6b**).

HRMS (pos. APCI): calculated for $C_{13}H_{17}O_2$ $[M+H]^+$ 205.1223, found 204.2690.

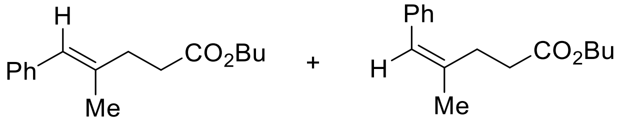

6c **7c**

Butyl (E)-4-methyl-5-phenylpent-4-enoate (6c), butyl (Z)-4-methyl-5-phenylpent-4-enoate (7c): The representative procedure was followed using 1-phenyl-1-propyne **5a** (25 µl, 0.20 mmol, 1.0 equiv.) and buthyl acrylate **2e** (114 µl, 0.80 mmol, 4.0 equiv.). After 16 h, flash chromatography (SiO_2, n-pentane:EtOAc = 100:1, R_f = 0.50; n-pentane:EtOAc = 10:1) afforded an inseparable mixture of **6c/7c** (38.88 mg, 80%, **6c:7c** = 90:10) as a colourless oil.

^{1}H-NMR (500 MHz, $CDCl_3$): 7.33-7.30 (m, **6c/7c**), 7.23-7.18 (m, **6b/7b**), 6.33 (s, 1H, **7c**), 6.30 (s, 1H, **6c**), 4.10 (t, J = 6.7 Hz, 2H, **6c**), 4.06 (t, J = 6.7 Hz, 2H, **7c**), 2.59-2.46 (m, 4H), 1.89 (d, *J* = 1.5 Hz, 3H, **7c**), 1.87 (d, *J* = 1.30 Hz, 1H, **6c**), 1.65-1.59 (m, 2H), 1.44-1.34 (m, 2H), 0.94-0.91

(m, 3H). **^{13}C-NMR** (125 MHz, $CDCl_3$): 173.38 (C, **6c**), 173.32 (C, **7c**), 138.25 (C, **6c**), 138.06 (C, **7c**), 137.14 (C, **6c**), 128.89 (CH, **6c**), 128.58 (CH, **7c**), 128.25 (CH, **7c**), 128.09 (CH, **6c**), 126.94 (CH, **7c**), 126.23 (CH, **7c**), 126.13 (CH, **6c**), 125.68 (CH, **6c**), 64.43 (CH_2, **7c**), 64.39 (CH_2, **6c**), 35.73 (CH_2, **6c**), 33.27 (CH_2, **6c**), 33.00 (CH_2, **7c**), 30.79 (CH_2, **6c**), 30.70 (CH_2, **7c**), 27.97 (CH_2, **7c**), 23.70 (CH_3, **6c**), 19.24 (CH_2, **7c**), 19.20 (CH_2, **7c**), 17.74 (CH_3, **6c**), 13.76 (CH_3).

HRMS (pos. ESI): calculated for $C_{16}H_{23}O_2$ $[M+H]^+$ 247.1698, found 246.1620.

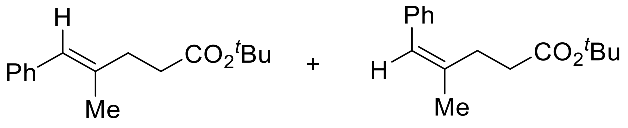

6d **7d**

tert-butyl (E)-4-methyl-5-phenylpent-4-enoate (6d), tert-butyl (Z)-4-methyl-5-phenylpent-4-enoate (7d): The representative procedure was followed using 1-phenyl-1-propyne **5a** (25 µl, 0.20 mmol, 1.0 equiv.) and *tert*-butyl acrylate **2b** (117 µl, 0.80 mmol, 4.0 equiv.). After 16 h, flash chromatography (SiO_2, n-pentane:EtOAc = 100:1, R_f = 0.57; n-pentane:EtOAc = 10:1) afforded an inseparable mixture of **6d/7d** (46.10 mg, 94%, **6c:7c** = 88:12) as a colourless oil.

^{1}H-NMR (400 MHz, $CDCl_3$): 7.34-7.29 (m, **6d/7d**), 7.23-7.17 (m, **6d/7d**), 6.32 (s, 1H, **7d**), 6.30 (s, 1H, **6d**), 2.47-2.45 (m, **6d/7d**), 1.89 (d, *J* = 1.5 Hz, 3H, **7d**), 1.87 (d, *J* = 1.30 Hz, 3H, **6d**), 1.46 (s, 9H, **6d**), 1.46 (s, 9H, **7d**). **^{13}C-NMR** (100 MHz, $CDCl_3$): 172.64 (C, **6d**), 138.34 (C, **6d**), 137.38 (C, **6d**), 128.90 (CH, **6c**), 128.11 (CH, **6c**), 126.08 (CH, **6d**), 125.60 (CH, **6d**), 80.32 (CH_2, **6d**), 35.84 (CH_2, **6d**), 34.42 (CH_2, **6d**), 28.23 (CH_3, **6d**), 17.73 (CH_3, **6d**).

HRMS (pos. ESI): calculated for $C_{16}H_{23}O_2$ $[M+H]^+$ 247.1653, found 246.1620.

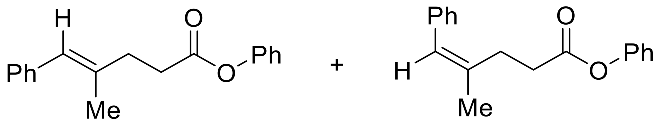

6e **7e**

Phenyl (E)-4-methyl-5-phenylpent-4-enoate (6e), phenyl (Z)-4-methyl-5-phenylpent-4-enoate (7e): The representative procedure was followed using 1-phenyl-1-propyne **5a** (25 µl, 0.20 mmol, 1.0 equiv.) and phenyl acrylate **2f** (110 µl, 0.80 mmol, 4.0 equiv.). After 16 h, flash chromatography (SiO_2, n-pentane:EtOAc = 100:1, R_f = 0.41; n-pentane:EtOAc = 10:1) afforded an inseparable mixture of **6e/7e** (39.77 mg, 75%, **6e:7e** = 88:12) as a colourless oil.

^{1}H-NMR (500 MHz, $CDCl_3$): 7.39-7.33 (m, **6e/7e**), 7.27-7.21 (m, **6e/7e**), 7.10-7.08 (m, **6e/7e**), 6.41 (s, 1H), 2.83-2.80 (m, **6e/7e**), 2.67 (m, **6e/7e**), 2.67-2.64 (m, **6e/7e**), 1.97 (d, *J* = 1.75 Hz, 3H, **7e**), 1.95 (d, *J* = 1.35 Hz, 1H, **6e**). **^{13}C-NMR** (125 MHz, $CDCl_3$): 171.75 (C, **6e**), 171.68 (C, **7e**), 150.81 (C, **6e**), 150.75 (C, **7e**), 138.11 (C, **6e**), 137.97 (C, **7e**), 136.74 (C, **6e**), 136.63 (C, **7e**), 129.49 (CH, **6e**), 129.45 (CH, **7e**), 128.93 (CH, **6e**), 128.01 (CH, **7e**), 128.34 (CH, **7e**), 128.16 (CH, **6e**), 127.38 (CH, **7e**), 126.36 (CH, **7e**), 126.27 (CH, **6e**), 126.16 (CH, **6e**), 125.85 (CH, **6e**), 121.64 (CH, **6e**), 121.58 (CH, **7e**), 35.71 (CH_2, **6e**), 33.27 (CH_2, **6e**), 32.98 (CH_2, **7e**), 27.85 (CH_2, **7e**), 23.71 (CH_3, **7e**), 17.74 (CH_3, **6**).

HRMS (pos. ESI): calculated for $C_{18}H_{19}O_2$ $[M+H]^+$ 267.1385, found 266.1307.

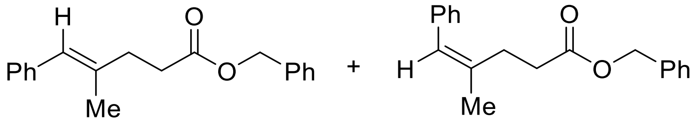

6f **7f**

Benzyl (E)-4-methyl-5-phenylpent-4-enoate (6f), benzyl (Z)-4-methyl-5-phenylpent-4-enoate (7f): The representative procedure was followed using 1-phenyl-1-propyne **5a** (25 µl, 0.20 mmol, 1.0 equiv.) and phenyl acrylate **2c** (110 µl, 0.80 mmol, 4.0 equiv.). After 16 h, flash chromatography (SiO_2, n-pentane:EtOAc = 100:1, R_f = 0.44; n-pentane:EtOAc = 10:1) afforded an inseparable mixture of **6f/7f** (39.77 mg, 70%, **6f:7f** = 98:2) as a colourless oil.

^{1}H-NMR (500 MHz, $CDCl_3$): 7.38-7.30 (m, 7H, **6f**), 7.22-7.19 (m, 3H, **6f**), 6.30 (s, 1H, **6f**), 2.63-2.60 (m, 2H, **6f**), 2.56-2.52 (m, 2H, **6f**), 1.87 (d, *J* = 1.35 Hz, 3H, **6f**). **^{13}C-NMR** (125 MHz, $CDCl_3$): 173.07 (C, **6f**), 138.19 (C, **6f**), 136.96 (C, **6f**), 136.08 (C, **6f**), 128.91 (CH, **6f**), 128.63 (CH, **6f**), 128.32 (CH, **6f**), 128.28 (CH, **6f**), 128.09 (CH, **6f**), 126.16 (CH, **6f**), 125.82 (CH, **6f**), 66.36 (CH_2, **6f**), 35.65 (CH_2, **6f**), 33.22 (CH_2, **6f**), 17.75 (CH_3, **6f**).

HRMS (pos. ESI): calculated for $C_{19}H_{23}O_2$ $[M+H]^+$ 281.1497, found 280.1463.

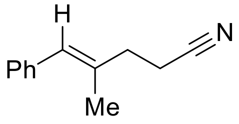

6g

(E)-4-methyl-5-phenylpent-4-enenitrile (6g): The representative procedure was followed using 1-phenyl-1-propyne **5a** (25 µl, 0.20 mmol, 1.0 equiv.) and acrylonitrile **2g** (85 µl, 0.80 mmol, 4.0 equiv.). After 16 h, flash chromatography (SiO_2, n-pentane:EtOAc = 40:1, R_f = 0.25; n-pentane:EtOAc = 10:1) afforded **6b** (11.74 mg, 35%) as a colourless oil.

^{1}H-NMR (500 MHz, $CDCl_3$): 7.28-7.23 (m, 2H), 7.18-7.14 (m, 3H), 6.32 (s, 1H), 2.58-2.55 (m, 2H), 2.53-2.50 (m, 2H), 1.81 (d, J = 1.5 Hz, 3H). **^{13}C-NMR** (125 MHz, $CDCl_3$): 137.48 (C), 134.50 (C), 128.93 (CH x 2), 128.22 (CH x 2), 127.74 (CH), 126.63 (CH), 119.32 (C), 35.92 (CH_2), 17.43 (CH_3), 16.47 (CH_2).

HRMS (pos. ESI): calculated for $C_{12}H_{14}N$ $[M+H]^+$ 172.1082, found 171.1082.

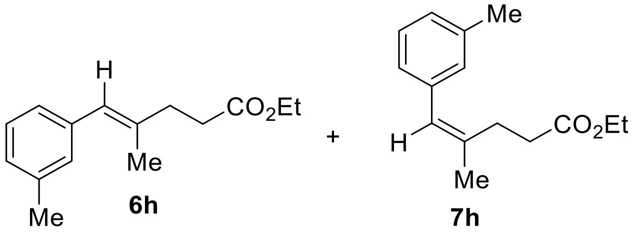

6h **7h**

ethyl (E)-4-methyl-5-(m-tolyl)pent-4-enoate (6h), ethyl (Z)-4-methyl-5-(m-tolyl)pent-4-enoate (7h): The representative procedure was followed using 1-methyl-3-(prop-1-yn-1-yl)benzene **5c** (26, 0.20 mmol, 1.0 equiv.) and ethyl acrylate **2a** (82 µl, 0.80 mmol, 4.0 equiv.). After 16 h, flash chromatography (SiO_2, n-pentane:EtOAc = 40:1, R_f = 0.42; n-pentane:EtOAc = 10:1) afforded an inseparable mixture of **6h/7h** (44.69 mg, 96%, **6h:7h** = 84:16) as a colourless oil.

^{1}H-NMR (400 MHz, $CDCl_3$): 7.22-7.18 (m, **6h/7h**), 7.03-7.00 (m, **6h/7h**), 6.30 (s, 1H, **7h**), 6.28 (s, 1H, **6h**), 4.15 (q, *J* = 7.2 Hz, 2H, **6h**), 4.11 (q, *J* = 7.2 Hz, 2H, **7h**), 2.55-2.48 (m, **6h/7h**), 2.34 (s, **6h/7h**), 1.88 (d, *J* = 1.48 Hz, 3H, **7h**), 1.87 (d, *J* = 1.4 Hz, 3H, **6h**), 1.27 (t, *J* = 7.2 Hz, 3H, **6h**), 1.24 (t, *J* = 7.2 Hz, 3H, **7h**). **^{13}C-NMR** (100 MHz, $CDCl_3$): 173.29 (C, **6h**), 173.25 (C, **7h**), 138.24 (C, **6h**), 138.05 (C, **7h**), 137.75 (C, **7h**),

137.63 (C, **6h**), 136.95 (C, **6h**), 129.66 (CH, **6h**), 129.39 (CH, **7h**), 128.17 (CH, **7h**), 128.02 (CH, **6h**), 127.03 (CH, **7h**), 127.02 (CH, **7h**), 126.92 (CH, **6h**), 125.95 (CH, **6h**), 125.80 (CH, **6h**), 125.62 (CH, **7h**), 60.43 (CH_2, **6h**), 35.75 (CH2, **6h**), 33.33 (CH_2, **6h**), 33.07 (CH_2, **7h**), 28.18 (CH_2, **7h**), 23.69 (CH_3, **7h**), 21.51 (CH_3, **6h**), 17.76 (CH_3, **6h**), 14.36 (CH_3, **6h**), 14.27 (CH_3, **7h**). (some signals could not be located likely due to overlapping).

HRMS (pos. APCI): calculated for $C_{15}H_{24}O_2N$ $[M+NH_4]^+$ 250.161, found 232.3230.

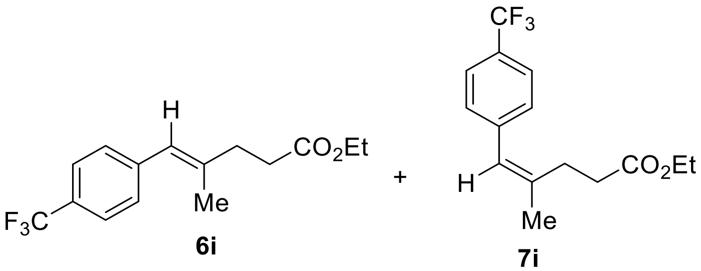

6i **7i**

ethyl (E)-4-methyl-5-(4-(trifluoromethyl)phenyl)pent-4-enoate (6d), ethyl (Z)-4-methyl-5-(4-(trifluoromethyl)phenyl)pent-4-enoate (7d): The representative procedure was followed using 1-(prop-1-yn-1-yl)-4-(trifluoromethyl)benzene **5d** (37, 0.20 mmol, 1.0 equiv.) and ethyl acrylate **2a** (82 µl, 0.80 mmol, 4.0 equiv.). After 16 h, flash chromatography (SiO_2, n-pentane:EtOAc = 40:1, R_f = 0.36; n-pentane:EtOAc = 10:1) afforded an inseparable mixture of **6i/7i** (50.53 mg, 88%, **6i:7i** = 86:14) as a colourless oil.

^{1}H-NMR (500 MHz, $CDCl_3$): 7.65-7.55 (m, **6i/7i**), 7.31-7.29 (m, **6i/7i**), 6.34 (s, 1H, **7i**), 6.31 (s, 1H, **6i**), 4.15 (q, *J* = 7.15 Hz, 2H, **6i**), 4.11 (q, *J* = 7.15 Hz, 2H, **7i**), 2.58-2.49 (m, **6i/7i**), 2.47-2.44 (m, **6i/7i**), 1.91 (d, *J* = 1.45 Hz, 3H, **7i**), 1.87 (d, *J* = 1.4 Hz, 3H, **6i**), 1.26 (t, *J* = 7.15 Hz, 3H, **6i**), 1.23 (t, *J* = 7.15 Hz, 3H, **7i**). **^{13}C-NMR** (125 MHz, $CDCl_3$): 173.07 (C, **6i**), 172.94 (C, **7i**), 141.87 (C, **6i**), 141.70 (C, **7i**), 139.59 (C, **6i**), 136.39 (C, **7i**), 129.07 (CH, **6i**), 128.84 (CH, **7i**), 128.07 (C, J_{C-F} = 30.97 Hz, **6i**), 125.82 (CH, **7i**), 125.21 (C, J_{C-F} = 3.92Hz, **7i**), 125.82 (C, J_{C-F} = 3.53 Hz, **6i**), 124.59 (CH, **6i**), 124.38 (CH, J_{C-F} = 269.95 Hz, **6i**), 60.59 (CH_2, **7i**), 60.53 (CH_2, **6i**), 35.64 (CH_2, **6i**), 33.06 (CH_2, **6i**), 32.76 (CH_2, **7i**), 27.88 (CH_2, **7i**), 23.69 (CH_3, **6i**), 17.81 (CH_3, **7i**), 14.34 (CH_3, **7i**), 14.23 (CH_3, **6i**) (some signals could not be located likely due to overlapping). **^{19}F-NMR** (300 MHz, $CDCl_3$): -62.409, -62.432.

HRMS (pos. APCI): calculated for $C_{15}H_{18}O_2F_3$ $[M+H]^+$ 287.1253, found 286.2942.

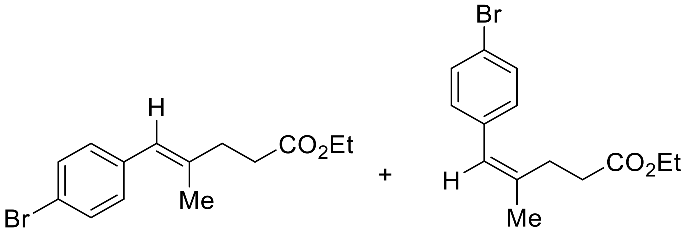

6j **7j**

ethyl (E)-5-(4-bromophenyl)-4-methylpent-4-enoate (6j), ethyl (Z)-5-(4-bromophenyl)-4-methylpent-4-enoate (7j): The representative procedure was followed using 1-bromo-4-(prop-1-yn-1-yl)benzene **5e** (37, 0.20 mmol, 1.0 equiv.) and ethyl acrylate **2a** (82 µl, 0.80 mmol, 4.0 equiv.). After 16 h, flash chromatography (SiO_2, n-pentane:EtOAc = 40:1, R_f = 0.45; n-pentane:EtOAc = 10:1) afforded an inseparable mixture of **6j/7j** (45.49 mg, 80%, **6j:7j** = 98:2) as a colourless oil.

^{1}H-NMR (500 MHz, $CDCl_3$): 7.43-7.41 (m, **6j/7j**), 7.08-7.06 (m, **6j/7j**), 6.24 (s, 1H, **7j**), 6.21 (s, 1H, **6j**), 4.14 (q, J = 7.1 Hz, 2H, **6j**), 2.53-2.46 (m, **6j/7j**), 1.87 (d, J = 1.5 Hz, 3H, **6j**), 1.83 (d, J = 1.4 Hz, 3H, **6j**), 1.27 (t, J = 7.15 Hz, 3H, **6j**). **^{13}C-NMR** (125 MHz, CDCl3): 173.15 (C), 138.11 (C), 137.12 (C), 131.22 (CH), 130.52 (CH), 124.61 (CH), 119.95 (C), 60.49 (CH_2), 35.66 (CH_2), 33.12 (CH_2), 17.74 (CH_3), 14.23 (CH_3).

HRMS (pos. APCI): calculated for $C_{14}H_{18}O_2Br$ $[M+H]^+$ 297.0485, found 296.1920.

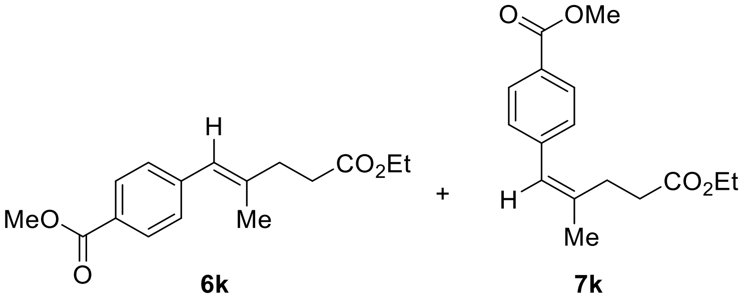

6k **7k**

methyl (E)-4-(5-ethoxy-2-methyl-5-oxopent-1-en-1-yl)benzoate (6k), methyl (Z)-4-(5-ethoxy-2-methyl-5-oxopent-1-en-1-yl)benzoate (7k): The representative procedure was followed using methyl 4-(prop-1-yn-1-yl)benzoate **5f** (36, 0.20 mmol, 1.0 equiv.) and ethyl acrylate **2** (82 µl, 0.80 mmol, 4.0 equiv.). After 16 h, flash chromatography (SiO_2, n-pentane:EtOAc = 40:1, R_f = 0.21; n-pentane:EtOAc = 10:1) afforded an inseparable mixture of **6k/7k** (46.45 mg, 81%, **6k:7k** = 64:36) as a colourless oil.

^{1}H-NMR (500 MHz, $CDCl_3$): 7.98-7.96 (m, **6k/7k**), 7.28-7.24 (m, **6k/7k**), 6.34 (s, 1H, **7k**), 6.32 (s, 1H, **6k**), 4.17-4.08 (m, **6k/7k**), 3.90 (s, **6k/7k**), 2.59-2.55 (m, 2H, **7k**), 2.53-2.52 (m, 4H, **6k**), 2.47-2.43 (m, 2H, **7k**), 1.90 (d, J = 1.9 Hz, 3H, **7k**), 1.88 (d, J = 1.7 Hz, 3H, **6k**), 1.27-120 (m, **6k/7k**). **^{13}C-NMR** (125 MHz, CDCl3): 173.07 (C, **6k**), 172.94 (C, **7k**), 167.05 (C, **6k**), 167.07 (C, **7k**), 143.03 (C, **6k**), 142.85 (C, **7k**), 134.60 (C, **6k**), 139.41 (C, **7k**), 129.64 (CH, **7k**), 129.48 (CH, **6k**), 128.79 (CH, **6k**), 128.55 (CH, **7k**), 127.94 (C, **7k**), 127.81 (C, **6k**), 126.29 (CH, **7k**), 125.08 (CH, **6k**), 60.56 (CH_2, **7k**), 60.50 (CH_2, **6k**), 52.04 (CH_3), 35.80 (CH_2, **6k**), 33.11 (CH_2, **6k**), 32.82 (CH_2, **7k**), 28.03 (CH_2, **7k**), 23.86 (CH_3, **7k**), 17.96 (CH_3, **6k**), 14.35 (CH_3, **6k**), 14.26 (CH_3, **7k**),

HRMS (pos. APCI): calculated for $C_{16}H_{20}O_4$ $[M+H]^+$ 277.0320, found 276.3320

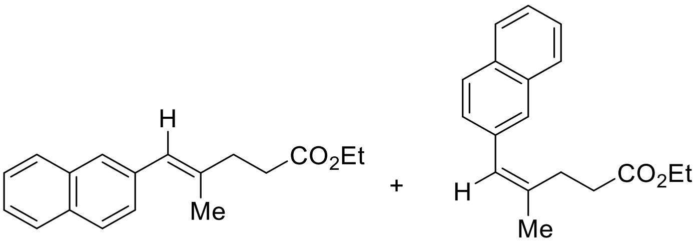

6l **7l**

ethyl (E)-4-methyl-5-(naphthalen-2-yl)pent-4-enoate (6l), ethyl (Z)-4-methyl-5-(naphthalen-2-yl)pent-4-enoate (7l): The representative procedure was followed using methyl 2-(prop-1-yn-1-yl)naphthalene **5g** (33, 0.20 mmol, 1.0 equiv.) and ethyl acrylate **2a** (82 µl, 0.80 mmol, 4.0 equiv.). After 16 h, flash chromatography (SiO_2, n-pentane:EtOAc = 40:1, R_f = 0.43; n-pentane:EtOAc = 10:1) afforded an inseparable mixture of **6l/7l** (37.57 mg, 70%, **6l:7l** = 54:46) as a colourless oil.

^{1}H-NMR (500 MHz, $CDCl_3$): 7.81-7.77 (m, **6l/7l**), 7.66-7.65 (m, **6l/7l**), 7.47-7.41 (m, **6l/7l**), 7.37-7.33 (m, **6l/7l**), 6.48 (s, 1H, **7l**), 6.45 (s, 1H, **6l**), 4.17 (q, J = 7.15 Hz, 2H, **6l**), 4.10 (q, J = 7.1 Hz, 2H, **7l**), 2.68-2.65 (m, 2H, **7l**), 2.58-2.56 (m, 4H, **6l**), 2.52-2.49 (m, 2H, **7l**), 1.95-1.94 (m, **6l/7l**), 1.28 (t, J = 7.15 Hz, 3H, **6l**), 1.22 (d, J = 7.1 Hz, 3H, **7l**). **^{13}C-NMR** (125 MHz, CDCl3): **^{13}C NMR** (125 MHz, CDCl3) δ 173.34 (C), 173.24 (C), 137.75 (C), 135.82 (C), 135.63 (C), 133.54 (C), 133.46 (C), 132.10 (C), 132.05 (C), 127.97 (CH), 127.91 (CH), 127.76 (CH), 127.66 (CH), 127.58 (CH), 127.40 (CH), 127.34 (CH), 127.05 (CH), 127.00 (CH), 126.08 (CH), 125.78 (CH), 125.62 (CH), 60.55 (CH_2), 60.54 (CH_2), 35.86 (CH_2, **6l**), 33.35 (CH_2, **6l**), 33.10 (CH_2, **7l**), 28.13 (CH_2, **7l**), 23.87 (CH_3), 17.95 (CH_3), 14.43 (CH_3, **6l**), 14.31 (CH_3, **6l**) (some signals could not be located likely due to overlapping).

HRMS (pos. ESI): calculated for $C_{18}H_{24}O_2N$ $[M+NH_4]^+$ 286.1802, found 268.1802.

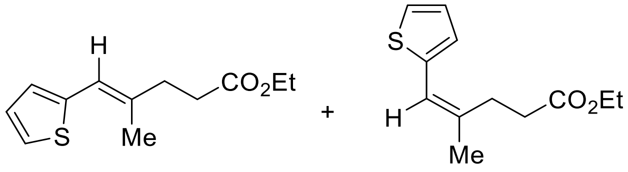

6m **7m**

ethyl (E)-4-methyl-5-(thiophen-2-yl)pent-4-enoate (6m), ethyl (Z)-4-methyl-5-(thiophen-2-yl)pent-4-enoate (7m): The representative procedure was followed using methyl 2-(prop-1-yn-1-yl)thiophene **5h** (25, 0.20 mmol, 1.0 equiv.) and ethyl acrylate **2a** (82 µl, 0.80 mmol, 4.0 equiv.). After 16 h, flash chromatography (SiO_2, n-pentane:EtOAc = 100:1, R_f = 0.48; n-pentane:EtOAc = 10:1) afforded an inseparable mixture of **6m/7m** (21.53 mg, 45%, **6m:7m** = 61:39) as a colourless oil.

^{1}H-NMR (500 MHz, $CDCl_3$): 7.21 (dd, J = 5.1, 0.95 Hz, 1H, **6m**), 7.19 (dd, J = 5.1, 1.1 Hz, 1H, **7m**), 7.00-6.97 (m, **6m/7m**), 6.92-6.91 (m, 1H, **6m**), 6.91-6.90 (m, 1H, **7m**), 6.43 (s, 1H, **6m**), 6.40 (s, 1H, **7m**), 4.17-4.11 (m, **6m/7m**), 2.74-2.71 (m, 2H, **7m**), 2.51-2.48 (m, **6m/7m**), 1.98 (d, J = 1.25 Hz, 3H, **6m**), 1.89 (d, J = 1.5 Hz, 3H, **7m**), 1.27-1023 (m, **6m/7m**). **^{13}C NMR** (125 MHz, CDCl3) δ 173.20 (C, **7m**), 173.15 (C, **6m**), 141.26 (C, **6m**), 140.55 (C, **7m**), 136.68 (C, **7m**), 136.01 (C, **6m**), 126.93 (CH, **7m**), 126.82 (CH, **6m**), 126.38 (CH, **6m**), 126.24 (CH, **7m**), 124.32 (CH, **6m**), 124.20 (CH, **7m**), 119.75(CH, **7m**) , 119.17 (CH, **6m**), 60.60 (CH_2, **7m**), 60.53 (CH_2, **6m**), 36.13 (CH_2), 33.32 (CH_2), 32.48 (CH_2), 28.85 (CH_2), 24.42 (CH_3), 18.50 (CH_3), 14.38 (CH_3), 14.34 (CH_3).

HRMS (pos. ESI): calculated for $C_{12}H_{20}O_2SN$ $[M+NH_4]^+$ 242.1072, found 224.0871.

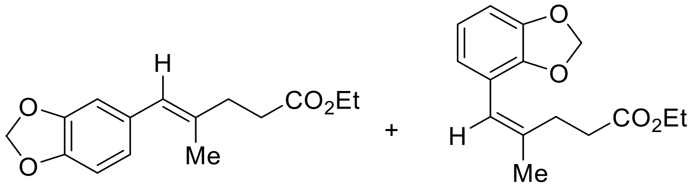

6n **7n**

ethyl (E)-5-(benzo[d][1,3]dioxol-5-yl)-4-methylpent-4-enoate (6n), ethyl (Z)-5-(benzo[d][1,3]dioxol-4-yl)-4-methylpent-4-enoate (7n): The representative procedure was followed using methyl 5-(prop-1-yn-1-yl)benzo[d][1,3]dioxole **5i** (33 mg, 0.20 mmol, 1.0 equiv.) and ethyl acrylate **2a** (82 µl, 0.80 mmol, 4.0 equiv.). After 16 h, flash chromatography (SiO_2, n-pentane:EtOAc = 40:1, R_f = 0.47; n-pentane:EtOAc = 10:1) afforded an inseparable mixture of **6n/7n** (21.53 mg, 34%, **6n:7n** = 69:31) as a colourless oil.

^{1}H-NMR (500 MHz, $CDCl_3$): δ 6.80 -6.75 (m, **6n/7n**), 6.73-6.72 (m, **6n/7n**), 6.70-6.63 (m, **6n/7n**), 6.23 (s, 1H, **7n**), 6.20 (s, 1H, **6n**), 5.94 (s, **6n/7n**), 4.16-4.09 (m, **6n/7n**), 2.572.42 (m, **6n/7n**), 1.85-1.84 (m, **6n/7n**), 1.27-1.23 (m, **6n/7n**). **^{13}C NMR** (125 MHz, $CDCl_3$): 173.34 (C, **6n**), 173.24 (C, **7n**), 147.53 (C, **7n**), 147.41 (C, **6n**), 145.44 (C, **7n**), 145.82 (C, **6n**), 136.38 (C, **7n**), 136.16 (C, **6n**), 132.37 (C, **6n**), 132.16 (C, **7n**), 126.52 (CH, **7n**), 125.33 (CH, **6n**), 122.44 (CH), 109.19 (CH), 108.98 (CH), 108.19 (CH), 108.09 (CH), 100.43 (CH_2), 60.51 (CH_2, **7n**), 60.45 (CH_2, **6n**), 35.86 (CH_2, **6n**), 33.29 (CH_2, **6n**), 32.91 (CH_2, **7n**), 27.88 (CH_2, **7n**), 23.60 (CH_3, **7n**), 17.74 (CH_3, **6n**), 14.37 (CH_3, **6n**), 14.27 (CH_3, **7n**).

HRMS (pos. ESI): calculated for $C_{15}H_{19}O_4$ $[M+H]^+$ 263.1275, found 262.3050.

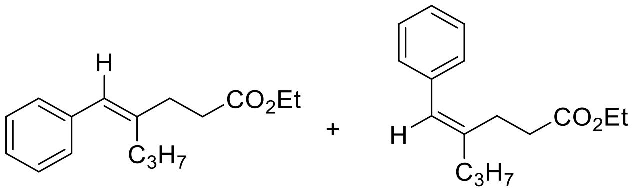

6o **7o**

ethyl (E)-4-benzylideneheptanoate (6o), ethyl (Z)-4-benzylideneheptanoate (7o): The representative procedure was followed using methyl 1-phenyl-1-pentyne **5k** (32 µl, 0.20 mmol, 1.0 equiv.) and ethyl acrylate **2a** (82 µl, 0.80 mmol, 4.0 equiv.). After 16 h, flash chromatography (SiO_2, n-pentane:EtOAc = 40:1, R_f = 0.40; n-pentane:EtOAc = 10:1) afforded an inseparable mixture of **6o/7o** (39.45 mg, 80%, **6o:7o** = 92:8) as a colourless oil.

^{1}H-NMR (500 MHz, $CDCl_3$): 7.32-7.2897 (m, **6o/7o**), 7.20-7.17 (m, **6o/7o**), 6.32 (s, 1H, **7o**), 6.30 (s, 1H, **6o**), 4.16 (q, J = 7.15 Hz, 2H, **6o**), 4.10 (q, J = 7.15 Hz, 2H, **7o**), 2.55-2.48 (m, 4H, **6o**), 2.22-2.19 (m, 2H, **6o**), 1.54-1.47 (m, 2H, **6o**), 1.27 (t, J = 7.15 Hz, 3H, **6o**), 1.23 (t, J = 7.15 Hz, 3H, **7o**), 0.96 (t, J = 7.3 Hz, 3H, **7o**), 0.90 (t, J = 7.35 Hz, 3H, **6o**). **^{13}C NMR** (125 MHz, $CDCl_3$): 173.37 (C, **6o**), 173.26 (C, **7o**), 141.65 (C, **6o**), 141.06 (C, **7o**), 138.29 (C, **6o**), 138.17 (C, **7o**), 128.70 (CH, **6o**), 128.64 (CH, **7o**), 128.27 (CH, **7o**), 128.15 (CH, **6o**), 126.52 (CH, **7o**), 126.22 (CH, **7o**), 126.20 (CH, **6o**), 125.75 (CH, **6o**), 60.45 (CH_2), 39.12 (CH_2, **7o**), 33.39 (CH_2, **6o**), 33.15 (CH_2, **7o**), 32.85 (CH_2, **6o**), 32.22 (CH_2, **6o**), 26.05 (CH_2, **7o**), 21.56 (CH_2, **6o**), 21.28 (CH_2, **7o**), 14.37 (CH_3, **6o**), 14.26 (CH_3, **7o**), 14.20 (CH_3, **6o**), 13.40 (CH_3, **7o**).

HRMS (pos. APCI): calculated for $C_{16}H_{23}O_2$ $[M+H]^+$ 247.1693, found 246.3500.

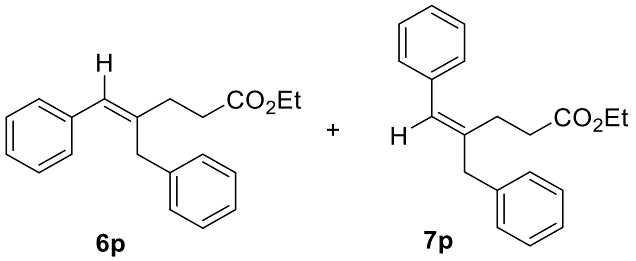

6p **7p**

ethyl (Z)-4-benzyl-5-phenylpent-4-enoate (6q), ethyl (E)-4-benzyl-5-phenylpent-4-enoate (7q): The representative procedure was followed using methyl prop-1-yne-1,3-diyldibenzene **5l** (38 mg, 0.20 mmol, 1.0 equiv.) and ethyl acrylate **2a** (82 µl, 0.80 mmol, 4.0 equiv.). After 16 h, flash chromatography (SiO_2, n-pentane:EtOAc = 40:1, R_f = 0.39; n-pentane:EtOAc = 10:1) afforded an inseparable mixture of **6p/7p** (35.65 mg, 61%, **6p:7p** = 94:6) as a colourless oil.

^{1}H-NMR (400 MHz, $CDCl_3$): 7.35-7.25 (m, **6p/7p**), 7.25-7.18 (m, **6p/7p**), 6.57 (s, 1H, **6 p**), 6.40 (s, 1H, **7 p**), 4.13 (q, J = 7.16 Hz, 2H, **6p**), 4.10 (q, J = 7.16 Hz, 2H, **7p**), 3.65 (s, 2H, **6p**), 3.52 (m, 2H, **7p**), 2.53-2.49 (m, **6p/7p**), 2.46-2.42 (m, **6p/7p**), 1.25 (t, J = 7.12 Hz, 3H, **6p**), 1.22 (t, J = 7.12 Hz, 3H, **7p**). **^{13}C NMR** (100 MHz, $CDCl_3$): 173.11 (C, **6p**), 172.99 (C, **7p**), 140.24 (C, **7p**), 139.46 (C, **7p**), 139.41 (C, **6p**), 139.12 (C, **6p**), 137.83 (CH, **6p**), 137.80 (CH, **7p**), 129.10 (CH, **7p**), 128.81 (CH, **7p**), 128.07 (CH, **7p**), 128.64 (CH, **6p**), 128.60 (CH, **6p**), 128.55 (CH, **6p**), 128.35 (CH, **6p**), 128.32 (CH, **7p**), 127.79 (CH, **6p**), 126.61 (CH, **6p**), 126.53 (CH, **7p**), 126.42 (CH, **7p**), 126.24 (CH, **6p**), 60.43 (CH_2, **6p**), 43.83 (CH_2, **7p**), 36.78 (CH_2, **6p**), 33.18 (CH_2, **6p**), 33.03 (CH_2, **7p**), 32.07 (CH_2, **6p**), 25.95 (CH_2, **7p**), 14.34 (CH_3, **6p**), 14.24 (CH_3, **7p**) (some signals could not be located likely due to overlapping).

HRMS (pos. APCI): calculated for $C_{20}H_{23}O_2$ $[M+H]^+$ 295.1653, found 295.1653.

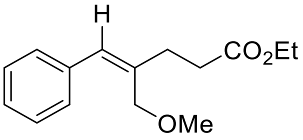

6q

ethyl (Z)-4-(methoxymethyl)-5-phenylpent-4-enoate (6q): The representative procedure was followed using (3-methoxyprop-1-yn-1-yl)benzene **5m** (30 mg, 0.20 mmol, 1.0 equiv.) and ethyl acrylate **2a** (82 µl, 0.80 mmol, 4.0 equiv.). After 16 h, flash chromatography (SiO_2, n-pentane:EtOAc = 40:1, R_f = 0.28; n-pentane:EtOAc = 10:1) afforded **6q** (32.22 mg, 63%) as a colourless oil.

^{1}H-NMR (500 MHz, $CDCl_3$): 7.34-7.30 (m, 1H), 7.25 -7.24 (m, 1H), 7.20 – 7.17 (m, 1H), 6.53 (s, 1H), 4.15 (q, J = 7.15 Hz, 2H), 4.04 (d, J = 0.6 Hz, 2H), 3.30 (s, 3H), 2.47-2.56 (m, 4H), 1.27 (t, J = 7.15 Hz, 3H). **^{13}C NMR** (125 MHz, $CDCl_3$): 173.27 (C), 137.84 (C), 137.07 (C), 129.79 (CH), 128.89 (CH), 128.22 (CH), 126.89 (CH), 70.50 (CH_2), 60.45 (CH_2), 58.24 (CH_3), 33.43 (CH_2), 31.12 (CH_3), 14.41 (CH_3).

HRMS (pos. ESI): calculated for $C_{15}H_{24}O_3N$ $[M+NH_4]^+$ 266.1752, found 248.32.

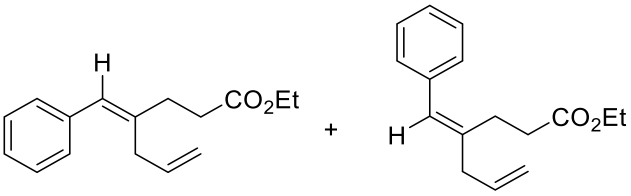

6r **7r**

ethyl (Z)-4-benzylidenehept-6-enoate (6r), ethyl (E)-4-benzylidenehept-6-enoate (7r): The representative procedure was followed using methyl pent-4-en-1-yn-1-ylbenzene **5n** (28 mg, 0.20 mmol, 1.0 equiv.) and ethyl acrylate **2a** (82 µl, 0.80 mmol, 4.0 equiv.). After 16 h, flash chromatography (SiO_2, n-pentane:EtOAc = 80:1, R_f = 0.48; n-pentane:EtOAc = 10:1) afforded an inseparable mixture of **6r/7r** (35.42 mg, 74%, **6r:7r** = 93:7) as a colourless oil.

^{1}H-NMR (500 MHz, $CDCl_3$): 7.32-7.29 (m, **6r/7r**), 7.22-7.18 (m, **6r/7r**), 6.41 (s, 1H, **6r**), 6.35 (s, 1H, **7r**), 5.91-5.83 (m, **6r/7r**), 5.14-5.10 (m, **6r/7r**), 4.16 (q, J = 7.15 Hz, 2H, **6r**), 4.10 (q, J = 7.1 Hz, 2H, **7r**), 3.00-2.98 (m, 2H, **6r**), 2.93-2.91 (m, 2H, **7r**), 2.61-2.43 (m, **6r/7r**), 1.27 (t, J = 7.15 Hz, 3H, **6r**), 1.23 (t, J = 7.1 Hz, 3H, **7r**). **^{13}C NMR** (125 MHz, $CDCl_3$): 173.23 (C, **6r**), 173.09 (C, **7r**), 139.31 (C, **7r**), 138.63 (C, **6r**), 137.88 (C, **7r**), 137.80 (C, **6r**), 136.22 (CH, **7r**), 135.87 (CH, **6r**), 128.61 (CH, **7r**), 128.56 (CH, **6r**), 128.31 (CH, **7r**), 128.21 (CH, **6r**), 127.64 (CH, **7r**), 127.01 (CH, **6r**), 126.47 (CH, **6r**), 126.43 (CH, **7r**), 116.84 (CH_2, **7r**), 116.43 (CH_2, **6r**), 60.48 (CH_2, **7r**), 60.46 (CH_2, **6r**), 41.57 (CH_2, **7r**), 35.50 (CH_2, **6r**), 33.43 (CH_2, **6r**), 32.97 (CH_2, **7r**), 32.28 (CH_2, **6r**), 26.32 (CH_2, **7r**), 14.36 (CH_3, **6r**), 14.25 (CH_3, **7r**).

HRMS (pos. APCI): calculated for $C_{16}H_{24}O_2N$ $[M+NH_4]^+$ 262.1801, found 244.3340.

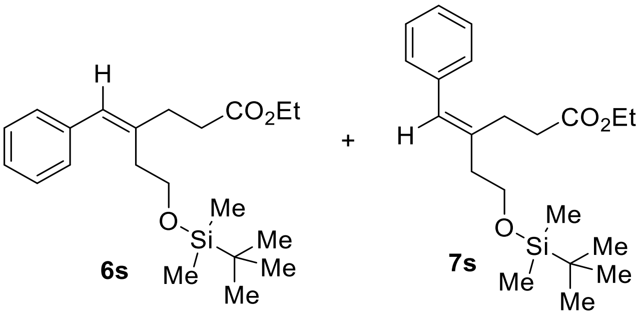

6s **7s**

Ethyl (Z)-4-benzylidene-6-((tert-butyldimethylsilyl)oxy)hexanoate (6s), ethyl (E)-4-benzylidene-6-((tert-butyldimethylsilyl)oxy)hexanoate (7s): The representative procedure was followed using tert-butyldimethyl((4-phenylbut-3-yn-1-yl)oxy)silane **5o** (52 mg, 0.20 mmol, 1.0 equiv.) and ethyl acrylate **2a** (82 µl, 0.80 mmol, 4.0 equiv.). After 16 h, flash chromatography (SiO_2, n-pentane:EtOAc = 80:1, R_f = 0.46; n-pentane:EtOAc = 10:1) afforded an inseparable mixture of **6s/7s** (54.36 mg, 75%, **6s:7s** = 91:98) as a colourless oil.

^{1}H-NMR (500 MHz, $CDCl_3$): 7.31-7.28 (m, **6s/7s**), 7.27-7.25 (m, **6s/7s**), 7.21-7.13 (m, **6s/7s**), 6.38 (s, 1H, **6s**), 6.36 (s, 1H, **7s**), 4.15 (q, J = 7.15 Hz, 2H, **6s**), 4.12-4.07 (m, 2H, **7s**), 3.79 (t, J = 6.8 Hz, 2H, **7s**), 3.74 (t, J = 7.0 Hz, 2H, **6s**), 2.54-2.53 (m, 4H, **6s**), 2.50-2.47 (m, 2H, **6s**), 1.26 (t, J = 7.15 Hz, 3H, **6s**), 1.24 (m, 3H, **7s**), 0.91 (s, 9H, **7s**) , 0.87 (s, 9H, **6s**), 0.74 (s, 6H, **7s**), 0.24 (s, 6H, **6s**). **^{13}C NMR** (125 MHz, $CDCl_3$): 173.28 (C, **6s**), 173.17 (C, **7s**), 141.22 (C, **7s**), 140.26 (C, **7s**), 138.45 (C, **6s**), 138.38 (C, **7s**), 137.95 (C, **6s**), 128.78 (CH, **6s**), 128.16 (CH, **6s**), 127.61 (CH, **6s**), 126.84 (CH, **7s**), 126.36 (CH, **6s**), 124.43 (CH, **7s**), 63.14 (CH_2, **7s**), 62.49 (CH_2, **7s**), 60.45 (CH_2, **6s**), 60.30 (CH_2, **7s**), 34.66 (CH_2, **7s**), 34.18 (CH_2, **6s**), 33.42 (CH_2, **6s**), 33.16 (CH_2, **7s**), 32.76 (CH_2, **6s**), 32.67 (CH_2, **7s**), 26.03 (CH_3, **7s**), 26.00 (CH_3, **6s**), 18.33 (CH_3, **7s**), 14.20 (CH_3, **6s)**, -5.29 (CH_3, **6s**) (some signals could not be located likely due to overlapping).

HRMS (pos. APCI): calculated for $C_{21}H_{34}O_3Si$ $[M+H]^+$ 363.2350, found 362.5850.

General procedure and characterization for diaryl substituted alkynes.

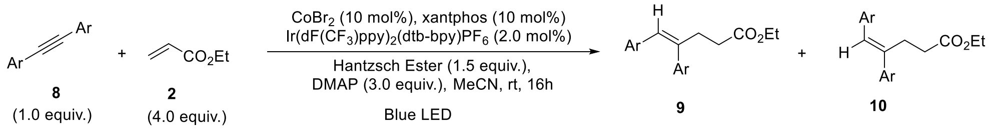

A flame-dried Schlenk tube was charged with $CoBr_2$ (4.37 mg, 0.02 mmol, 10 mol%), xantphos (8.25 mg, 0.02 mmol, 10 mol%), $Ir(dF(CF_3)ppy)_2(dtb\text{-}bpy)PF_6$ (4.48 mg, 0.02 mmol, 2.0 mol%), Hantzsch ester (75.99 mg, 0.30 mmol, 1.5 equiv.) and DMAP (73.30 mg, 0.6 mmol, 3.0 equiv). The tube was evacuated and backfilled with argon for three times. 2 ml of MeCN was added and the mixture was stirred for 10 min at room temperature. Then, the corresponding alkyne (0.2 mmol, 1.0 equiv.) and the ethyl acrylate **2** (4.0 equiv.) were added. The resulting mixture was irradiated with a 4.8 W Blue LED strip and stirred at room temperature for 16h. The crude was filtered through a short pad of silica (3 cm) and washed with DCM, dried under vacuum and analysed by ^{1}H-NMR. The resulting residue was purified by flash chromatography (n-pentane/ethyl acetate). ^{1}H-NMR, ^{13}C-NMR spectra and HR-MS were recorded from the mixture; only unambiguous NMR signals were assigned).

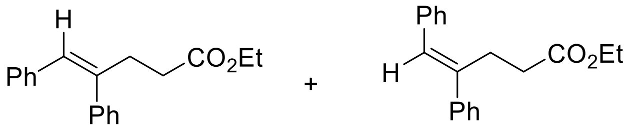

9a **10a**

Ethyl (Z)-4,5-diphenylpent-4-enoate (9a), ethyl (E)-4,5-diphenylpent-4-enoate (10a): The representative procedure was followed using diphenylacetylene **8a** (37.83 mg, 0.20 mmol, 1.0 equiv.) and ethyl acrylate **2a** (82 µl, 0.80 mmol, 4.0 equiv.). After 16 h, flash chromatography (SiO_2, n-pentane:EtOAc = 80:1, R_f = 0.34; pentane:EtOAc = 100:1) afforded an inseparable mixture of **9a/10a** (57.90 mg, 97%, **9a:10a** = 76:24) as a colourless oil.

^{1}H-NMR (400 MHz, $CDCl_3$): 7.47-7.44 (m, **9a/10a**), 7.40-7.34 (m, **9a/10a**), 7.32-7031 (m, **9a/10a**), 7.17-7.14 (m, **9a/10a**), 7.10-7.03 (m, **9a/10a**), 6.92-6.89 (m, **9a/10a**), 6.75 (s, 3H, **10a**), 6.49 (s, 1H, **9a**), 4.11 (q, J = 7.16 Hz, 2H, **9a**), 4.05 (q, J = 7.16 Hz, 2H, **10a**), 3.09-3.05 (m, 2H, **10a**), 2.85-2.81 (m, **9a**), 2.43-2.37 (m, **9a /10a**), 1.24 (t, J = 7.12 Hz, 2H, **9a**), 1.19 (t, J = 7.12 Hz, 2H, **10a**). **^{13}C-NMR** (100 MHz, $CDCl_3$): 173.11 (C, **9a**), 173.03 (C, **10a**), 142.16 (C, **10a**), 141.38 (C, **9a**), 141.16 (C, **10a**), 140.44 (C, **9a**), 137.85 (CH, **10a**), 137.15 (CH, **9a**), 129.53 (CH, **10a**), 129.11 (CH, **9a**), 128.80 (CH, **10a**), 128.76 (CH, **9a**), 128.73 (CH, **9a**), 128.60 (CH, **10a**), 128.49 (CH, **10a**), 127.93 (CH, **9a**), 127.55 (CH, **10a**), 127.25 (CH, **9a**), 126.92 (CH, **10a**), 126.79 (CH, **10a**), 126.46 (CH, **9a**), 60.45 (CH_2, **10a**), 60.42 (CH_2, **9a**), 35.93 (CH_2, **9a**), 33.50 (CH_2, **10a**), 33.22 (CH_2, **9a**), 25.79 (CH_2, **10a**), 14.35 (CH_3, **9a**), 14.34 (CH_3, **10a**).

HRMS (pos. APCI): calculated for $C_{19}H_{21}O_2$ $[M+H]^+$ 281.1536, found 280.3670

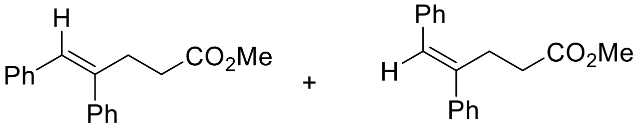

9b **10b**

Ethyl (Z)-4,5-diphenylpent-4-enoate (9b), methyl (E)-4,5-diphenylpent-4-enoate (10b): The representative procedure was followed using diphenylacetylene **8a** (37.83 mg, 0.20 mmol, 1.0 equiv.) and methyl acrylate **2d** (82 µl, 0.80 mmol, 4.0 equiv.). After 16 h, flash chromatography (SiO_2, n-pentane:EtOAc = 40:1, R_f = 0.34; pentane:EtOAc = 10:1) afforded an inseparable mixture of **9b/10b** (54.26 mg, 98%, **9b:10b** = 76:24) as a colourless oil.

^{1}H-NMR (300 MHz, $CDCl_3$): 7.38-7.17 (m, **9b/10b**), 7.09-7.05 (m, **9b/10b**), 7.00-6.96 (m, **9b/10b**), 6.85-6.81 (m, **9b/10b**), 6.67 (s, 1H, **10b**), 6.40 (s, 1H, **9b**), 3.56 (s, 3H, **9b**), 3.51 (s, 3H, **10b**), 3.02-2.96 (m, 2H, **10b**), 2.78-2.72 (m, 2H, **9b**), 2.37-2.29 (m, **9b /10b**). **^{13}C-NMR** (100 MHz, $CDCl_3$): 173.49 (C, **9b**), 173.40 (C, **10b**), 142.04 (C, **10b**), 141.26 (C, **9b**), 141.01 (C, **10b**), 140.34 (C, **9b**), 137.80 (C, **10b**), 137.09 (C, **9b**), 129.55 (CH, **10b**), 129.09 (CH, **9b**), 128.75 (CH, **10b**), 128.72 (CH, **9b**), 128.59 (CH, **10b**), 128.47 (CH, **10b**), 127.91 (CH, **9b**), 127.55 (CH, **10b**), 127.56 (CH, **9b**), 126.91 (CH, **10b**), 126.74 (CH, **10b**), 126.746 (CH), 51.58 (CH_3, **9a/10b**), 35.87 (CH_2, **9b**), 33.21 (CH_2, **10b**), 33.97 (CH_2, **9b**), 25.75 (CH_2, **10b**).

HRMS (pos. APCI): calculated for $C_{18}H_{19}O_2$ $[M+H]^+$ 267.1340, found 266.1307.

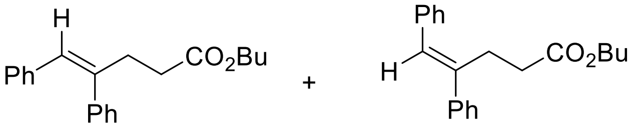

9c **10c**

Butyl (Z)-4,5-diphenylpent-4-enoate (9c), butyl (E)-4,5-diphenylpent-4-enoate (10c): The representative procedure was followed using diphenylacetylene **8a** (37.83 mg, 0.20 mmol, 1.0 equiv.) and butyl acrylate **2e** (82 µl, 0.80 mmol, 4.0 equiv.). After 16 h, flash chromatography (SiO_2, n-pentane:EtOAc = 100:1, R_f = 0.47; pentane:EtOAc = 10:1) afforded an inseparable mixture of **9c/10c** (34.91 mg, 56%, **9c:10c** = 78:22) as a colourless oil.

^{1}H-NMR (500 MHz, $CDCl_3$): 7.38-7.24 (m, **9c/10c**), 7.17-7.15 (m, **9c/10c**), 7.10-7.06 (m, **9c/10c**), 6.92-6.90 (m, **9c/10c**), 6.75 (s, 1H, **10c**), 6.49 (s, 1H, **9c**), 4.07 (q, J = 6.7 Hz, 2H, **9c**), 4.00 (q, J = 6.7 Hz, 2H, **10c**), 3.09-3.06 (m, 2H, **10c**), 2.85-2.82 (m, 2H, **9c**), 2.44-2.39 (m, **9c/10c**), 1.62-1.54 (m, **9c/10c**), 1.41-1.31 (m, **9c/10c**), 0.94-0.90 (m, **9c /10c**). **^{13}C-NMR** (125 MHz, $CDCl_3$): 173.21 (C, **9c**), 173.12 (C, **10c**), 142.09 (C, **10c**), 141.33 (C, **9c**), 141.10 (C, **10c**), 140.39 (C, **9c**), 137.80 (C, **10c**), 137.10 (C, **9c**), 126.47 (CH, **10c**), 129.09 (CH, **9c**), 128.78 (CH, **10c**), 128.72 (CH, **9c**), 128.59 (CH, **10c**), 128.47 (CH, **10c**), 127.90 (CH, **9c**), 127.54 (CH, **10c**), 127.24 (CH, **9c**), 127.20 (CH, **9c**), 126.90 (CH, **10c**), 126.75 (CH, **10c**), 126.44 (CH, **9c**), 64.41 (CH_2, **10c**), 64.38 (CH_2, **9c**), 35.91 (CH_2, **9c**), 33.48 (CH_2, **10c**), 33.17 (CH_2, **9c**), 30.79 (CH_2, **9c**), 30.07 (CH_2, **10c**), 25.78 (CH_2, **10c**), 19.22 (CH_2, **9c**), 19.18 (CH_2, **10c**), 13.77 (CH_2, **9c**).

HRMS (pos. APCI): calculated for $C_{21}H_{25}O_2$ $[M+H]^+$ 308.4210, found 309.1810.

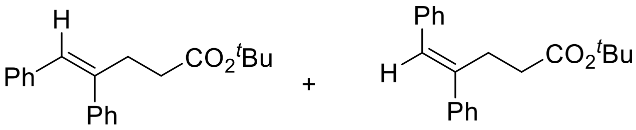

9d **10d**

***tert*-Butyl (Z)-4,5-diphenylpent-4-enoate (9d), tert-butyl (E)-4,5-diphenylpent-4-enoate (10d):** The representative procedure was followed using diphenylacetylene **8a** (37.83 mg, 0.20 mmol, 1.0 equiv.) and *tert*-butyl acrylate **2b** (117 µl, 0.80 mmol, 4.0 equiv.). After 16 h, flash chromatography (SiO_2, n-pentane:EtOAc = 80:1, R_f = 0.50; pentane:EtOAc = 10:1) afforded an inseparable mixture of **9d/10d** (25.25 mg, 40%, **9d:10d** = 79:21) as a colourless oil.

^{1}H-NMR (500 MHz, $CDCl_3$): 7.32-7.24 (m, **9d/10d**), 7.17-7.15 (m, **9d/10d**), 7.09-7.05 (m, **9d/10d**), 6.91-6.89 (m**9d/10d**), 6.73 (s, 1H, **10d**), 6.48 (s, 1H, **9d**), 3.05-3.02 (m, 2H, **10d**), 2.80-2.76 (m, **9d/10d**), 2.34-2.29 (m, **9d/10d**), 1.44 (s, **9d/10d**). **^{13}C-NMR** (125 MHz, $CDCl_3$): 172.48 (C, **9d**), 141.55 (C, **9d**), 170.56 (C, **9d**), 137.20 (C, **9d**), 129.32 (C, **10d**), 129.08 (C, **9d**), 128.85 (CH, **10d**), 128.75 (CH, **9d**), 128.70 (CH, **9d**), 128.57 (CH, **10d**), 128.46 (CH, **10d**), 127.91 (CH, **9d**), 127.49 (CH, **10d**), 127.18 (CH, **9d**), 127.12 (CH, **9d**), 126.86 (CH, **10d**), 126.79 (CH, **10d**), 126.38 (CH, **9d**), 80.37 (CH_2), 36.09 (CH_2, **9d**), 34.69 (CH_2, **10d**), 34.25 (CH_2, **9d**), 28.22 (CH_3, **9d**), 28.15 (CH_3, **10d**), 25.86 (CH_2, **10d**).

HRMS (pos. APCI): calculated for $C_{21}H_{25}O_2$ $[M+H]^+$ 309.1810, found 308.4210.

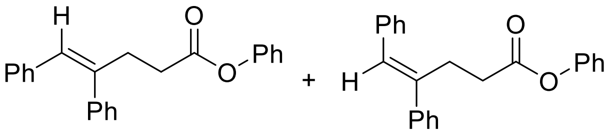

9e **10e**

Phenyl (Z)-4,5-diphenylpent-4-enoate (9e), phenyl (E)-4,5-diphenylpent-4-enoate (10e): The representative procedure was followed using diphenylacetylene **8a** (37.83 mg, 0.20 mmol, 1.0 equiv.) and *tert*-butyl acrylate **2f** (110 µl, 0.80 mmol, 4.0 equiv.). After 16 h, flash chromatography (SiO_2, n-pentane:EtOAc = 100:1, R_f = 0.41; pentane:EtOAc = 10:1) afforded an inseparable mixture of **9e/10e** (50.60 mg, 76%, **9e:10e** = 78:22) as a colourless oil.

^{1}H-NMR (500 MHz, $CDCl_3$): 7.52-7.50 (m, **9e/10e**), 7.43-7.28 (m, **9e/10e**), 7.24-7.21 (m, **9e/10e**), 7.12-7.04 (m, **9e/10e**), 7.00-6.98 (m, **9e/10e**), 6.95-6.93(m, **9e/10e**), 6.81 (s, 1H, **10e**), 6.59 (s, 1H, **9e**), 3.05-3.02 (m, 2H, **10e**), 2.99-2.95 (m, 2H, **9e**), 2.69-2.64 (m, **9e/10e**).**^{13}C-NMR** (125 MHz, $CDCl_3$): 171.59 (C, **9e**), 171.49 (C, **10e**), 150.79 (C, **9e**), 150.72 (C, **10e**), 141.98 (C, **10e**), 140.92 (C, **9e**), 140.75 (C, **10e**), 140.20 (C, **9e**), 137.73 (C, **10e**), 136.97 (C, **9e**), 129.89 (CH, **10e**), 129.49 (CH, **9e**), 129.43 (CH, **10e**), 129.14 (CH, **9e**), 128.85 (CH, **9e**), 128.81 (CH, **10e**), 128.79 (CH, **9e**), 128.72 (CH, **10e**), 128.55 (CH, **10e**), 127.97 (CH, **9e**), 127.67 (CH, **9e**), 127.40 (CH, **10e**), 127.02 (CH, **10e**), 126.84 (CH, **10e**), 127.58 (CH, **9e**), 125.86 (CH, **9e**), 125.82 (CH, **10e**), 121.65 (CH, **9e**), 121.57 (CH, **10e**), 35.92 (CH_2, **9e**), 33.48 (CH_2, **10e**), 33.18 (CH_2, **9e**), 25.68 (CH_2, **10c**).

HRMS (pos. APCI): calculated for $C_{23}H_{21}O_2$ $[M+H]^+$ 329.1497, found 328.4110.

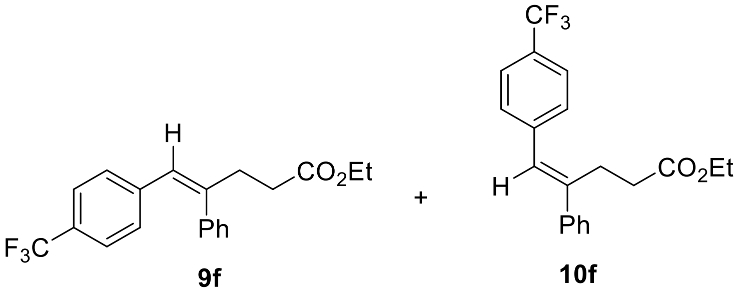

9f **10f**

ethyl (Z)-4-phenyl-5-(4-(trifluoromethyl)phenyl)pent-4-enoate (9f), ethyl (E)-4-phenyl-5-(4-(trifluoromethyl)phenyl)pent-4-enoate (10f): The representative procedure was followed using 1-(phenylethynyl)-4-(trifluoromethyl)benzene **8b** (37.83 mg, 0.20 mmol, 1.0 equiv.) and ethyl acrylate **2a** (43 mg, 0.80 mmol, 4.0 equiv.). After 16 h, flash chromatography (SiO_2, n-pentane:EtOAc = 80:1, R_f = 0.44; pentane:EtOAc = 10:1) afforded an inseparable mixture of **9f/10f** (29.25mg, 42%, **9f:10f** = 90:10) as a colourless oil.

^{1}H-NMR (500 MHz, $CDCl_3$): 7.33-7.27 (m, **9f/10f**), 7.13-7.09 (m, **9f/10f**), 6.99-6.98 (m, **9f/10f**), 4.13-4.04 (m, 2H), 6.57 (s, 1H, **10f**), 6.50 (s, 1H, **9f**), 4.13-4.04 (m, **9f/10f**), 2.87-2.84 (m, **9f/10f**), 2.43-2.40 (m, **9f/10f**), 1.24 (t, J = 7.15 Hz, 3H). **^{13}C-NMR** (125 MHz, C_6H_6): 172.05 (C, **9f**), 171.98 (C, **10f**), 144.28 (C), 141.04 (C), 140.03 (C), 129.50 (CH, **9f**), 129.48 (CH, **10f**), 129.34 (CH, **10f**), 129.03 (CH, **9f**), 128.66 (CH, **9f**), 128.52 (CH, **10f**), 126.09 (CH, **9f**), 125.03 (C, $J_{C\text{-}F}$ = 3.76, **9f**), 60.21 (CH_2, **9f**), 60.17 (CH_2, **10f**), 36.03 (CH_2, **9f**), 35.51 (CH_2, **10f**), 32.92 (CH_2, **9f**), 32.81 (CH_2, **10f**), 14.25 (CH_3, **9f**). **^{19}F-NMR** (300 MHz, $CDCl_3$): -62.495 (**10f**), -62.539 (**9f**).

HRMS (pos. APCI): calculated for $C_{20}H_{20}O_2F_3$ $[M+H]^+$ 349.1410, found 348.3652.

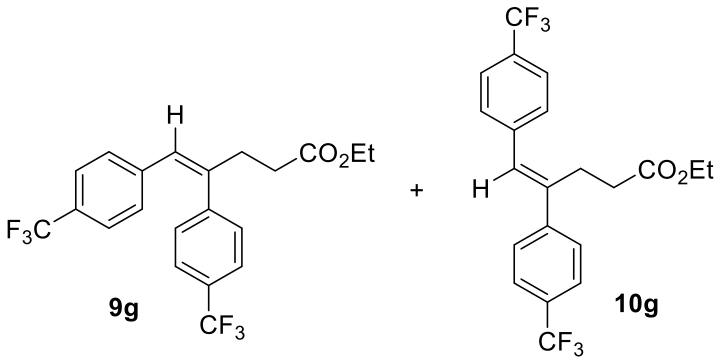

The representative procedure was followed using diphenylacetylene 1,2-bis(4-(trifluoromethyl)phenyl)ethyne **8c** (64 mg, 0.20 mmol, 1.0 equiv.) and ethyl acrylate **2a** (82 µl, 0.80 mmol, 4.0 equiv.). After 16 h, flash chromatography (SiO_2, n-pentane:EtOAc = 40:1) afforded **9g** (18.27 mg, 22%, R_f = 0.45; pentane:EtOAc = 40:1) and **10g** (60.73 mg, 72%, R_f = 0.27; pentane:EtOAc = 40:1) as colourless oils (94% combined yield, **9g:10g** = 75:25).

Ethyl (Z)-4,5-bis(4-(trifluoromethyl)phenyl)pent-4-enoate (9g)

^{1}H-NMR (500 MHz, $CDCl_3$): 7.58-7.57 (m, 2H), 7.36-7.35 (m, 2H), 7.28-7.26 (m, 2H), 6.98-6.97 (m, 2H), 6.59 (s, 1H), 4.12 (q, 2H, J = 7.1 Hz), 2.88-2.58 (m, 2H), 2.41 (t, J = 7.85 Hz, 2H), 1.24 (t, J = 7.1 Hz, 3H). **^{13}C-NMR** (125 MHz, $CDCl_3$): 172.59 (C), 143.62 (C), 142.41 (C), 140.08 (C), 129.88 (C, $J_{C\text{-}F}$ = 32.44 Hz), 129.24 (CH), 129.10 (CH), 128.73 (C, $J_{C\text{-}F}$ = 32.34 Hz), 127.16 (CH), 125.91 (CH, $J_{C\text{-}F}$ = 3.95 Hz), 125.07 (CH, $J_{C\text{-}F}$ = 3.97 Hz), 124.18 (C, $J_{C\text{-}F}$ = 273.77 Hz), 60.63 (CH_2), 35.47 (CH_2), 32.80 (CH_2), 14.31 (CH_3). **^{19}F-NMR** (300 MHz, $CDCl_3$): -62.59, -62.38

HRMS (pos. APCI): calculated for $C_{21}H_{22}O_2NF_6$ $[M+NH_4]^+$ 434.1546, found 416.3434.

Ethyl (E)-4,5-bis(4-(trifluoromethyl)phenyl)pent-4-enoate (10g)

^{1}H-NMR (500 MHz, $CDCl_3$): 7.66-7.40 (m, 4H), 7.57-7.55 (m, 2H), 7.45-7.44 (m, 2H), 6.78 (s, 1H), 4.05 (q, 2H, J = 7.15 Hz), 3.07-3.04 (m, 2H), 2.37-2.34 (m, 2H), 1.19 (t, J = 7.15 Hz, 3H). **^{13}C-NMR** (125 MHz, $CDCl_3$): 172.47 (C), 145.20 (C), 142.01 (C), 140.78 (C), 129.99 (C, $J_{C\text{-}F}$ = 32.37 Hz), 129.75 (CH), 129.31 (C, $J_{C\text{-}F}$ = 32.43 Hz), 129.08 (CH), 127.17 (CH), 125.69 (2xCH, $J_{C\text{-}F}$ = 3.5 Hz), 125.55 (2xCH, $J_{C\text{-}F}$ = 3.59 Hz), 124.20 (C, $J_{C\text{-}F}$ = 273.79 Hz), 60.71 (CH_2), 33.07 (CH_2), 25.68 (CH_2), 14.19 (CH_3). **^{19}F-NMR** (300 MHz, $CDCl_3$): -62.558

HRMS (pos. APCI): calculated for $C_{21}H_{22}O_2NF_6$ $[M+NH_4]^+$ 434.1546, found 416.3434.

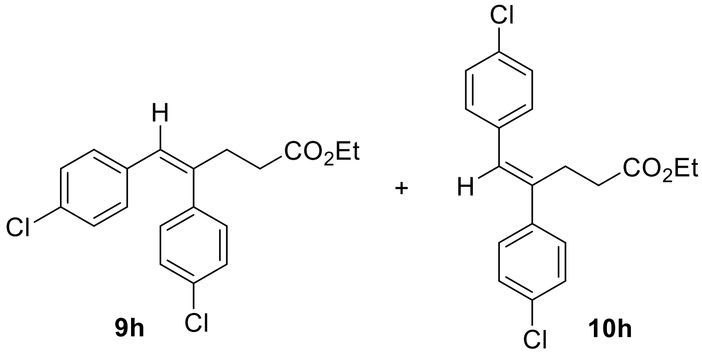

9h **10h**

The representative procedure was followed using diphenylacetylene 11,2-bis(4-chlorophenyl)ethyne **8d** (49 mg, 0.20 mmol, 1.0 equiv.) and ethyl acrylate **2a** (82 µl, 0.80 mmol, 4.0 equiv.). After 16 h, flash chromatography (SiO_2, n-pentane:EtOAc = 40:1) afforded **9h** (32.37 mg, 47%, R_f = 0.33; pentane:EtOAc = 40:1) and **10h** (17.65 mg, 26%, R_f = 0.42; pentane:EtOAc = 40:1) as colourless oils (72% combined yield, **9h:10h** = 68:32).

ethyl (Z)-4,5-bis(4-chlorophenyl)pent-4-enoate (9h)

^{1}H-NMR (500 MHz, $CDCl_3$): 7.29-7.26 (m, 2H), 7.08-7.06 (m, 4H), 6.84-6.81 (m, 2H), 6.45 (s, 1H), 4.10 (q, 2H, J = 7.12 Hz), 2.88-2.58 (m, 2H), 2.41 (t, J = 7.85 Hz, 2H), 1.24 (t, J = 7.1 Hz, 3H). **^{13}C-NMR** (125 MHz, $CDCl_3$): **^{13}C-NMR** (125 MHz, $CDCl_3$): 172.81 (C), 140.79 (C), 138.34 (C), 135.24 (C), 133.36 (C), 132.38 (C), 130.32 (CH), 130.12 (CH), 129.12 (CH), 128.26 (CH), 126.66 (CH), 60.54 (CH_2), 35.51 (CH_2), 32.95 (CH_2), 14.23 (CH_3).

HRMS (pos. APCI): calculated for $C_{19}H_{22}O_2NCl_2$ $[M+NH_4]^+$ 366.1022, found 349.25.

ethyl (E)-4,5-bis(4-chlorophenyl)pent-4-enoate (10h)

^{1}H-NMR (500 MHz, $CDCl_3$): 7.37-7.33 (m, 6H), 7.26-7.24 (m, 2H), 6.65 (s, 1H), 4.06 (q, J = 7.15 Hz, 2H), 2.81-2.78 (m, 2H), 2.39-2.36 (m, 2H), 1.23 (t, J = 7.15 Hz, 3H). **^{13}C-NMR** (125 MHz, $CDCl_3$): 172.70 (C), 140.72 (C), 140.23 (C), 135.88 (C), 133.57 (C), 132.91 (C), 136.08 (CH), 128.81 (CH), 128.72 (CH), 128.05 (CH), 60.62 (CH_2), 33.20 (CH_2), 25.63 (CH_2), 14.23 (CH_3).

HRMS (pos. APCI): calculated for $C_{19}H_{22}O_2NCl_2$ $[M+NH_4]^+$ 366.1022, found 349.2510.

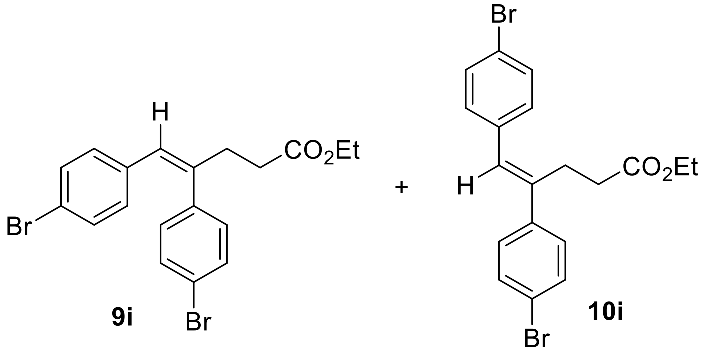

9i **10i**

The representative procedure was followed using diphenylacetylene 1,2-bis(4-bromophenyl)ethyne **8e** (64 mg, 0.20 mmol, 1.0 equiv.) and ethyl acrylate **2a** (82 µl, 0.80 mmol, 4.0 equiv.). After 16 h, flash chromatography (SiO_2, n-pentane:EtOAc = 80:1) afforded **9i** (49.32 mg, 66%, R_f = 0.36; pentane:EtOAc = 40:1) and **10i** (15.48 mg, 21%, R_f = 0.60; pentane:EtOAc = 40:1) as colourless oils (87% combined yield, **9i:10i** = 75:25).

ethyl (Z)-4,5-bis(4-bromophenyl)pent-4-enoate (9i)

^{1}H-NMR (500 MHz, $CDCl_3$): 7.44-7.42 (m, 2H), 7.24-7.21 (m, 2H), 7.02-6.99 (m, 2H), 6.78-6.75 (m, 2H), 6.43 (s, 1H), 4.10 (q, 2H, J = 7.15 Hz), 2.80-2.77 (m, 2H), 2.39-2.34 (m, 2H), 1.23 (t, J = 7.15 Hz, 3H). **^{13}C-NMR** (125 MHz, $CDCl_3$): 172.78 (C), 140.93 (C), 138.79 (C), 135.66 (C), 132.08 (CH), 131.23 (CH), 130.64 (CH), 130.43 (CH), 126.70 (CH), 121.54 (C), 120.59 (C), 60.54 (CH_2), 35.47 (CH_2), 32.92 (CH_2), 14.34 (CH_3).

HRMS (pos. APCI): calculated for $C_{19}H_{22}O_2NBr_2$ $[M+NH_4]^+$ 455.9991, found 438.1890.

ethyl (E)-4,5-bis(4-bromophenyl)pent-4-enoate (10i)

^{1}H-NMR (500 MHz, $CDCl_3$): 7.51-7.48 (m, 4H), 7.31-7.29 (m, 2H), 7.19-7.17 (m, 2H), 6.63 (s, 1H), 4.06 (q, J = 7.15 Hz, 2H), 3.01-2.97 (m, 2H), 2.36-2.33 (m, 2H), 1.99 (t, J = 7.15 Hz, 3H). **^{13}C-NMR** (125 MHz, $CDCl_3$): 172.67 (C), 140.84 (C), 140.69 (C), 136.32 (C), 131.78 (CH), 131.68 (CH), 130.39 (CH), 128.77 (CH), 128.38 (CH), 121.71 (C), 121.07 (C), 60.63 (CH_2), 33.18 (CH_2), 25.58 (CH_2), 14.24 (CH_3).

HRMS (pos. APCI): calculated for $C_{19}H_{19}O_2Br_2$ $[M+H]^+$ 436.9746, found 438.1890.

Deuteration labelling experiments

The reaction was carried out with deuterated Hantzsch ester (**11**) under the standard conditions to gain more insights for the mechanism (Scheme S1). This transformation shows that two of the deuterium atoms of **11** are incorporated into the final products (Figure S1). The deuteration of Hc is more evident in the ^{13}C-NMR, see Figure S2.

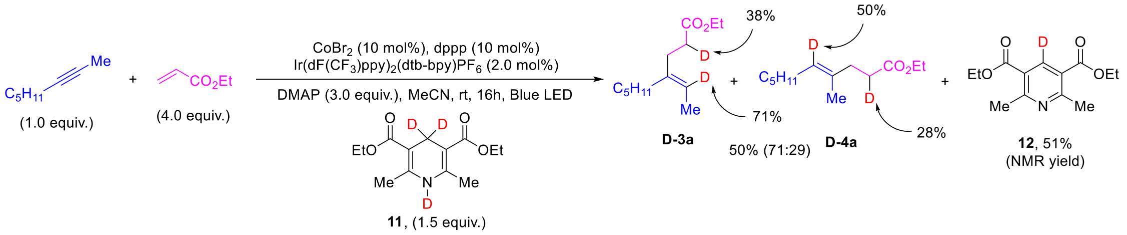

Scheme S1. Reaction employing deuterated Hantzsch ester (**11**)

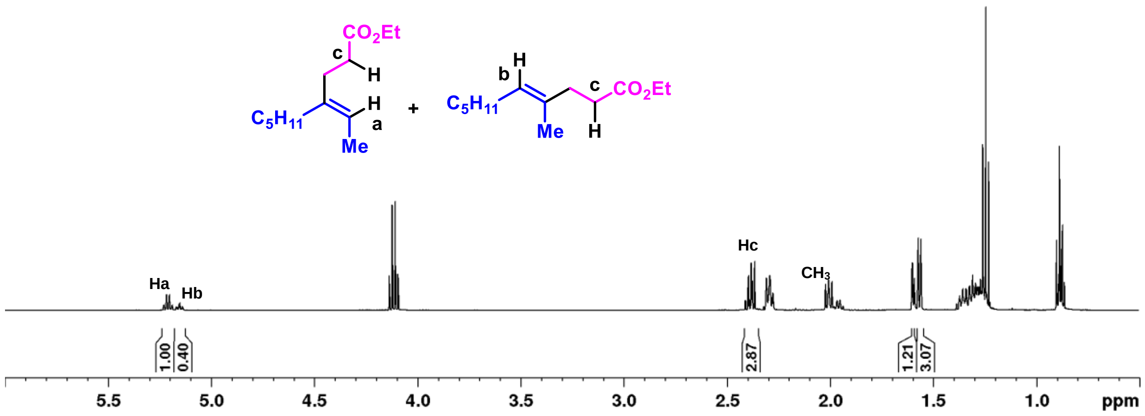

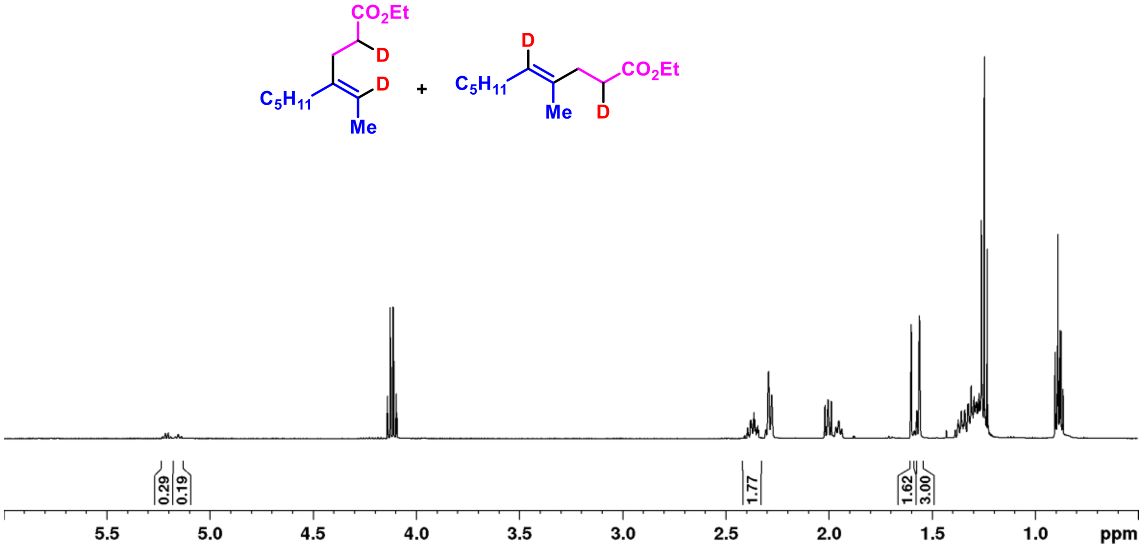

Figure S1. ^{1}H-NMR of no deuterated vs. deuterated compounds.

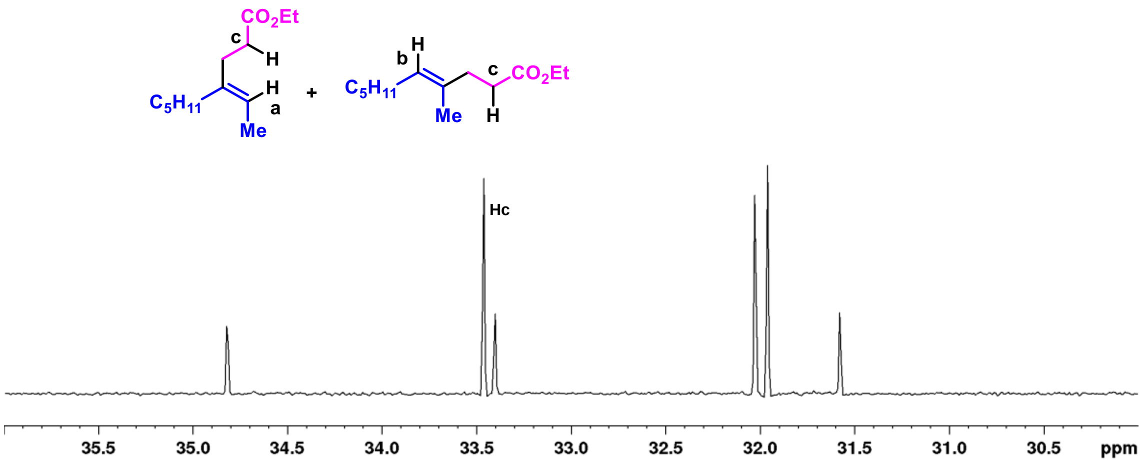

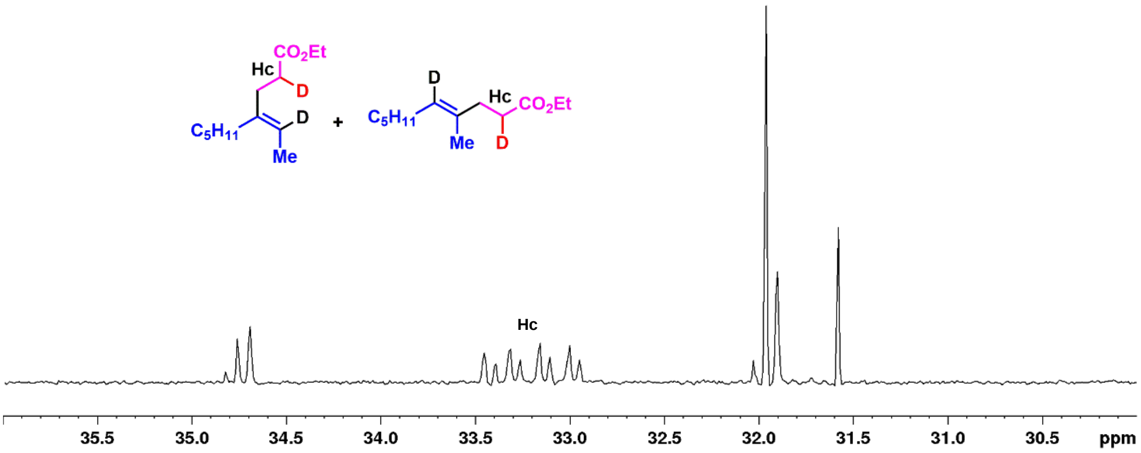

Figure S2. ^{13}C-NMR of no deuterated vs. deuterated compounds.

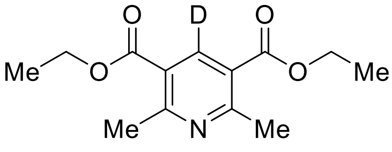

12

Diethyl 2,6-dimethylpyridine-3,5-dicarboxylate-4-d (12): ^{1}H-NMR (400 MHz, $CDCl_3$): 4.39 (q, *J* = 7.12 Hz, 4H), 2.84 (s, 3H), 1.41 (t, *J* = 7.12, 3H). **^{13}C-NMR** (100 MHz, $CDCl_3$): 166.05 (C), 162.30 (C), 123.14 (C), 61.48 (CH_2), 24.99 (CH_3), 14.37 (CH_3). R_f = 0.15; n-pentane:EtOAc = 10:1. White solid (mp = 71-74 ºC).

HR-MS (ESI) calc. for $[C_{13}H_{17}{}^{2}HO_4N]^+$ 253.1291, found 252.2881.

Quantum yield measurement

Determination of the light intensity of the blue LED

The photon flux of blue LED was determined by standard ferrioxalate actinometry. A 0.15 M solution of ferrioxalate was prepared by dissolving potassium ferrioxalate hydrate (1.47 g, 3.0 mmol) in 20.0 mL of 0.20 M aqueous sulfuric acid. A 0.15 M buffered solution of 1,10-phenanthroline was prepared by dissolving 1,10-phenanthroline (1.35 g, 7.5 mmol) and sodium acetate (3.08 g, 37.5 mmol) in 50 mL of 0.20 M aqueous sulfuric acid. To a 10 ml Schlenk tube equipped with a stir bar was added 1.0 mL of the ferrioxalate solution. The tube was sealed and placed 2.5 cm away from one 34 W blue LED. After irradiation for 5 seconds, 3.0 mL of the aqueous sulfuric acid and 4.0 mL of the buffered solution was added to the vial. The solution was then allowed to rest for 1 hour to allow the resultant ferrous ions to react completely with 1,10-phenanthroline. 50 μL of the resulting solution was taken as an aliquot and diluted with 2.0 mL of 0.20 M aqueous sulfuric acid. The absorbance of the resulting solution in a cuvette (l = 1.0 cm) at 510 nm was measured by UV-Vis spectrometer. A non-irradiated sample and other samples with different irradiation time were also prepared and the absorbance at 510 nm was measured. The moles of Fe^{2+} formed were determined using Beer's Law:

$$\text{moles } Fe^{2+} = \frac{V_1 \times V_3 \times \Delta A}{10^3 \times V_2 \times l \times \varepsilon}$$

Where V_1 (2.05mL) is the irradiated volume, V_2 (0.05 mL) is the aliquot of the irradiated solution taken for the determination of the ferrous ions. V_3 (8mL) is the final volume after complexation with phenanthroline (all in mL), l is the path length (1 cm), and ΔA (510 nm) is the optical difference in absorbance between the irradiated and non-irradiated solutions, ε (510 nm) is the molar absorptivity of $Fe(phen)_3^{2+}$ (11100 L $mol^{-1}cm^{-1}$). The moles of Fe^{2+} formed for each sample are shown below:

**Irradiation time (s)**	5.34	10.09	15.39	20.34	25.31
**ΔA**	0.4052	0.5495	0.6572	0.8351	0.9724
**Fe^{2+}**	1.19	1.62	1.94	2.27	2.87

The moles of Fe2+ formed are plotted as a function of time (t). The slope is shown as: d (moles Fe^{2+})/dt = 8.38×10^{-7}

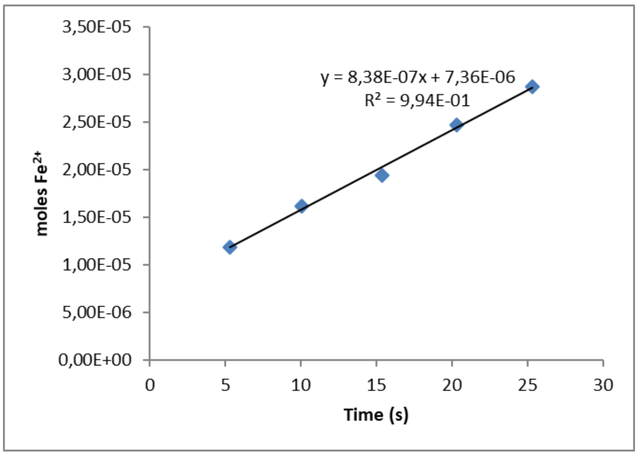

Figure S3. Moles of Fe^{2+} *vs.* time (t).

The photon flux can be calculated as:

$$\text{photo flux (Einstein.s}^{-1}\text{)} = \frac{\text{moles Fe}^{2+}}{\Phi \cdot t \cdot f} = \frac{\frac{d(\text{moles Fe}^{2+})}{dt}}{\Phi \cdot f} = \frac{8.38 \times 10^{-7}}{0.842 \cdot 0.966} = 1.03 \times 10^{-6}$$

Where Φ is the quantum yield for the ferrioxalate actinometer (0.845 for a 0.15 solution at 457.9 nm), t is the irradiated time, and f is the fraction of light absorbed at λ = 450 nm ($f = 1–10^{-A}$). The absorbance of the ferrioxalate solution at 450 nm is 1.472 indicating f ($f = 1–10^{-A}$) is 0.966.

Measurement of quantum yield

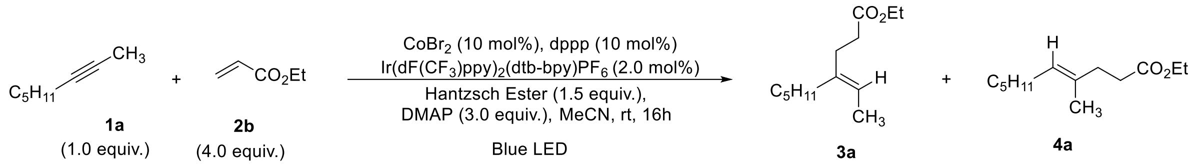

A flame-dried Schlenk tube was charged with $CoBr_2$ (4.37 mg, 0.02 mmol, 10 mol%), dppp (8.25 mg, 0.02 mmol, 10 mol%), $Ir(dF(CF_3)ppy)_2(dtb\text{-}bpy)PF_6$ (4.48 mg, 0.02 mmol, 2.0 mol%), Hantzsch ester (75.99 mg, 0.30 mmol, 1.5 equiv.) and DMAP (73.30mg, 0.6 mmol, 3.0 equiv). The tube was evacuated and backfilled with argon for three times. 1 ml of MeCN was added and the mixture was stirred for 10 min at room temperature. Then, 2-octyne (**1a**) (0.2 mmol, 1.0 equiv.) and ethyl acrylate (**2a**) (4.0 equiv.) were added. The resulting mixture was placed approximately 2.5 cm away from one 34 W blue LED and irradiated and stirred for 1 hour at room temperature. The solvents were removed under reduced pressure. The moles of **3a/4a** were measured by ^{1}H NMR using 1,2-dichloroethane as internal standard (7.98×10^{-5} mol). The quantum yield calculation is then as following:

$$\Phi = \frac{\text{moles of product}}{\text{moles of absorbed photons}} = \frac{\text{moles of product}}{\text{flux} \cdot t \cdot f} = \frac{7.98 \times 10^{-5}}{1.03 \times 10^{-6} \cdot 3600 \cdot 0.379} = 0.056$$

Where flux is the photon flux determined by ferrioxalate actinometry (1.03×10^{-6} Einstein/s), t is the time (3600 s), and f is the fraction of light absorbed by $Ir(dF(CF_3)ppy)_2(dtb\text{-}bpy)PF_6$ at 450 nm. A 2×10^{-3} M solution of $Ir(dF(CF_3)ppy)_2(dtb\text{-}bpy)PF_6$ in MeCN was prepared. The absorbance of the solution at 450 nm was 0.379. The fraction of light absorbed at 450 nm was calculated: $f = 1.0000 - 10^{-A} = 1.0000 - 10^{-0.379} = 0.58$.

Stern-Volmer fluorescence quenching studies

Rates of quenching (k_q) were determined using Stern-Volmer kinetics: $\frac{I_0}{I} = k_q\ \tau_0\ [Quencher] + 1$

Were I_0 is the luminescence intensity without the quencher, I is the intensity with the quencher, and τ_0 is the lifetime of the photocatalyst (2300 ns for $[Ir(dF(CF_3)ppy)_2(dtb\text{-}bpy)PF_6]$ in acetonitrile).[6]

The quenching studies were carried out using a 1.28×10^{-3} M solution of $[Ir(dF(CF_3)ppy)_2(dtb\text{-}bpy)PF_6$ in acetonitrile and a 6.66×10^{-3} M solution of the potential quencher in acetronitrile. The samples were prepared in 10 ml volumetric flasks adding to each of them 100 µl of the solution of $[Ir(dF(CF_3)ppy)_2(dtb\text{-}bpy)PF_6$ and increasing amounts of the quencher solution (0, 100 µl, 200 µl, 300 µl, 600 µl, 1200 µl). The samples were measured in a 12.5 x 12.5 x 56 mm quartz cuvette with screw cap. All the samples were irradiated at 375 nm and the fluorescence emissions were detected at 473 nm.

A. Evaluation of 1a as potential quencher for [Ir(dF(CF$_3$)ppy)$_2$(dtb-bpy)PF$_6$].

Not quenching is observed.

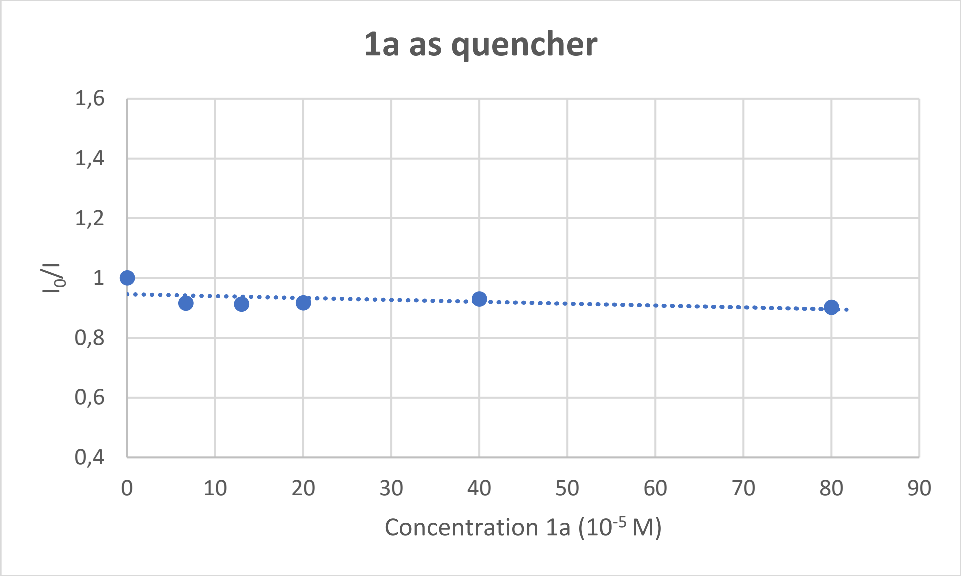

Figure S4. Stern-Volmer plot of [Ir(dF(CF$_3$)ppy)$_2$(dtb-bpy)PF$_6$] at variable concentrations of **1a**.

B. Evaluation of 2a as potential quencher for [Ir(dF(CF$_3$)ppy)$_2$(dtb-bpy)PF$_6$].

Not quenching is observed.

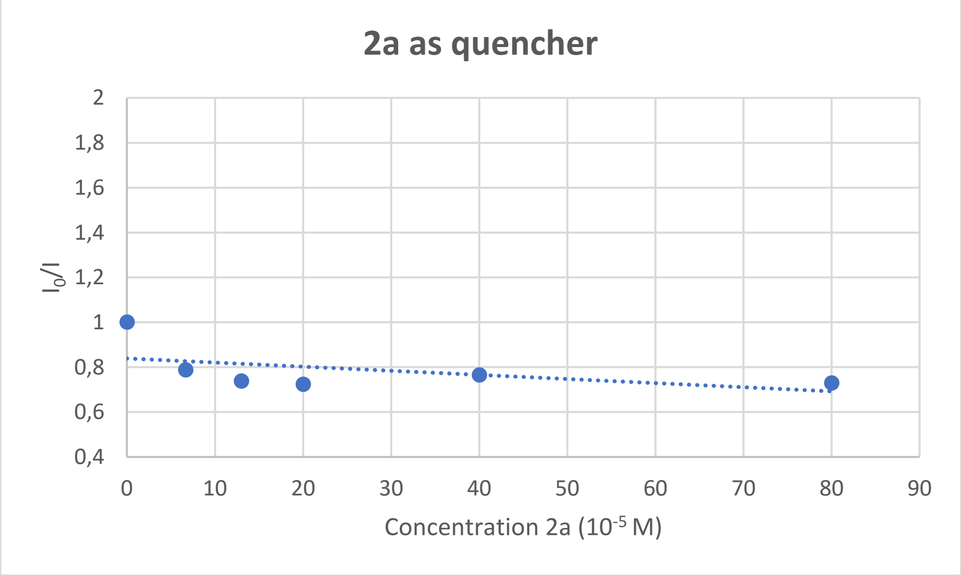

Figure S5. Stern-Volmer plot of [Ir(dF(CF$_3$)ppy)$_2$(dtb-bpy)PF$_6$] at variable concentrations of **2a**.

C. Evaluation of the Hantzsch ester as potential quencher for [Ir(dF(CF$_3$)ppy)$_2$(dtb-bpy)PF$_6$].

Fluorescence quenching was observed with the Hantzsch ester. $K_q = 9.7 \times 10^3\ M^{-1}\ s^{-1}$.

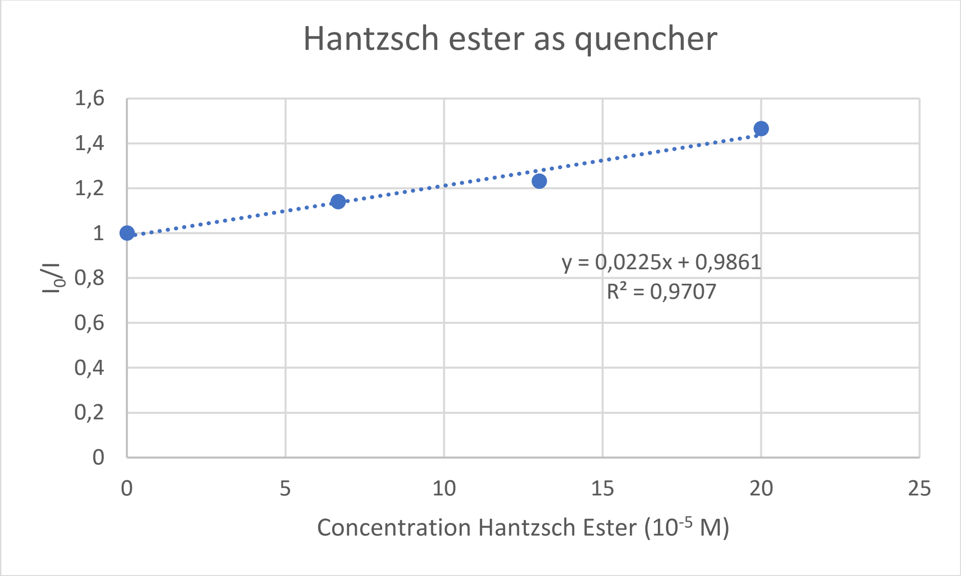

Figure S5. Stern-Volmer plot of [Ir(dF(CF$_3$)ppy)$_2$(dtb-bpy)PF$_6$] at variable concentrations of the **Hantzsch ester**.

On/Off studies

On/Off studies were performing in an NMR tube charged with $CoBr_2$ (1.31 mg, 0.006 mmol, 10 mol%), dppp (2.47 mg, 0.006 mmol, 10 mol%), Ir(dF(CF$_3$)ppy)$_2$(dtb-bpy)PF$_6$ (1.3 mg, 0.001 mmol, 2.0 mol%), Hantzsch ester (22.79 mg, 0.09 mmol, 1.5 equiv.) and DMAP (21.99 mg, 0.18 mmol, 3.0 equiv). The tube was evacuated and backfilled with argon for three times. 0.6 ml of acetonitrile-d$_3$, **1a** (8.82 µl, 0.06 mmol, 1.0 equiv.), **2a** (26 µl, 0.24 mmol, 4.0 equiv.) and internal standard dichloromethane (4 µl, 0.06 mmol, 1.0 equiv.) were added under a flow of argon. The NMR tube was sealed with a cap and covered with parafilm. The mixture was monitored by ^{1}H NMR alternating cycles of irradiation and darkness. As it is shown in Figure S6, the reaction is stopped in darkness and re-started under irradiation.

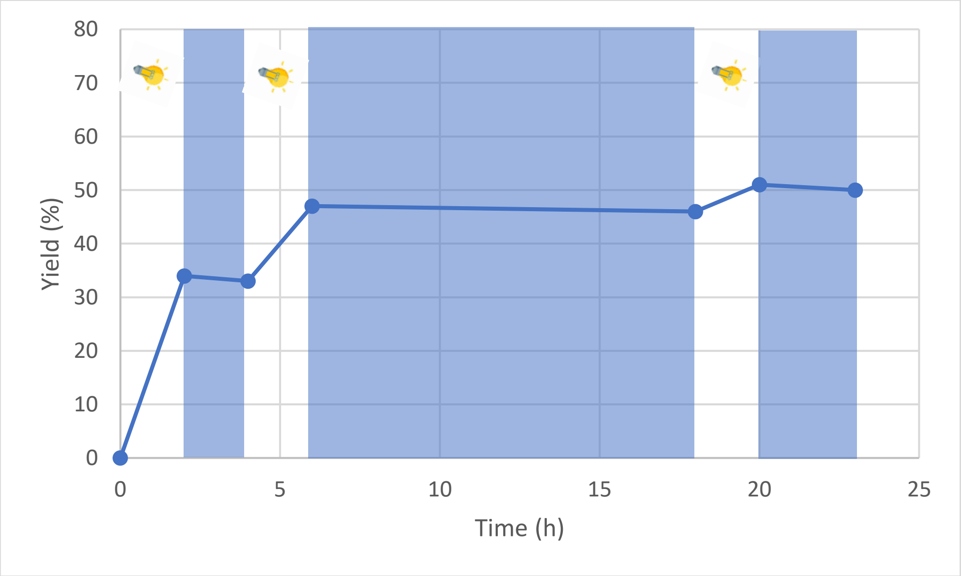

Figure S6. Reaction profile through on/off studies.

References

[1] a) W. Zhang, S. Kraft, J. S. Moore, *J. Am. Chem. Soc.* **2004**, *126*, 329-335; b) A. M. Haydl, L. J. Hilpert, B. Breit, *Chem.–Eur. J.* **2016**, *22*, 6547-6551; c) T. M. Beck, B. Breit, *Eur. J. Org. Chem.* **2016**, 5839-5844; d) S. Gao, H. Liu, Z. Wu, H. Yao and A. Lin, *Green Chem* **2017**, *19*, 1861-1865; e) Z.-W, Chen, Y.-Z, Zhu, J.-W, Ou, Y-P, Wang, J.-Y, Zheng. *J. Org. Chem.* **2014**, *79*, 10988-10998.

[2] H.-J. Xu, Y-S. Kang, H. Shi, P. Zhang, Y.-K. Chen, B. Zhang, Z.-Q. Liu, J. Zhao, W.-Y. Sun, J.-Q. Yu, Yi Lu, *J. Am. Chem. Soc.* **2019**, *141*, 76-79.

[3] L. M. Schneider, V. M. Schmiedel, T. Pecchioli, D. Lentz, Ch. Merten, M. Christmann, *Org. Lett.* **2017**, *19*, 2310-3213.

[4] M. Das, M. Duy Vu, Q. Zhang, X.-W. Liu, *Chem. Sci.* **2019**, *10*, 1687-1691.

[5] a) J. C. Tellis, D. N. Primer, G. A. Molander, *Science* **2014**, *345*, 433-436; b) M. Lee, S. Neukirchen, Ch. Cabrele, O.Reiser, *J. Pept. Sci.* **2017**, *23*, 566-573.

[6] a) S. Mukherjee, B. Maji, A. Tlahuext-Aca, F. Glorius, *J. Am. Chem. Soc.* **2016**, *138*, 16200−16203. b) M. S. Lowry, J. D. Goldsmith, J. D. Slinker, R. Rohl, R. A. Pascal, C. G. Malliaras, S. Bernhard, *Chem. Mater.* **2005**, *17*, 5712-5719.

Copies of NMR experiments of the products

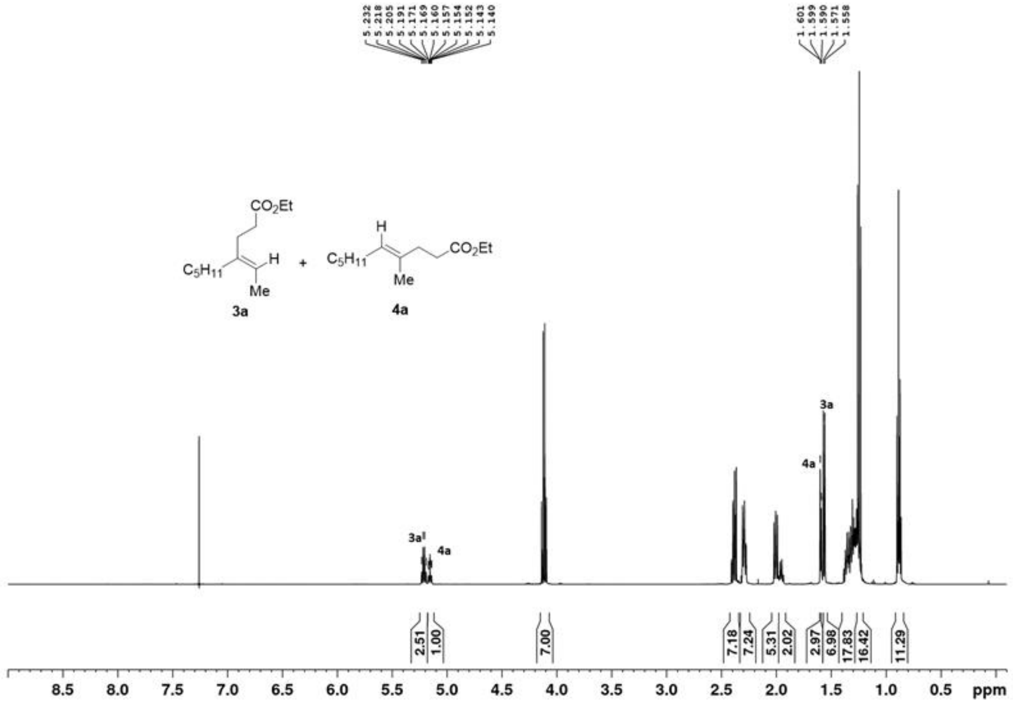

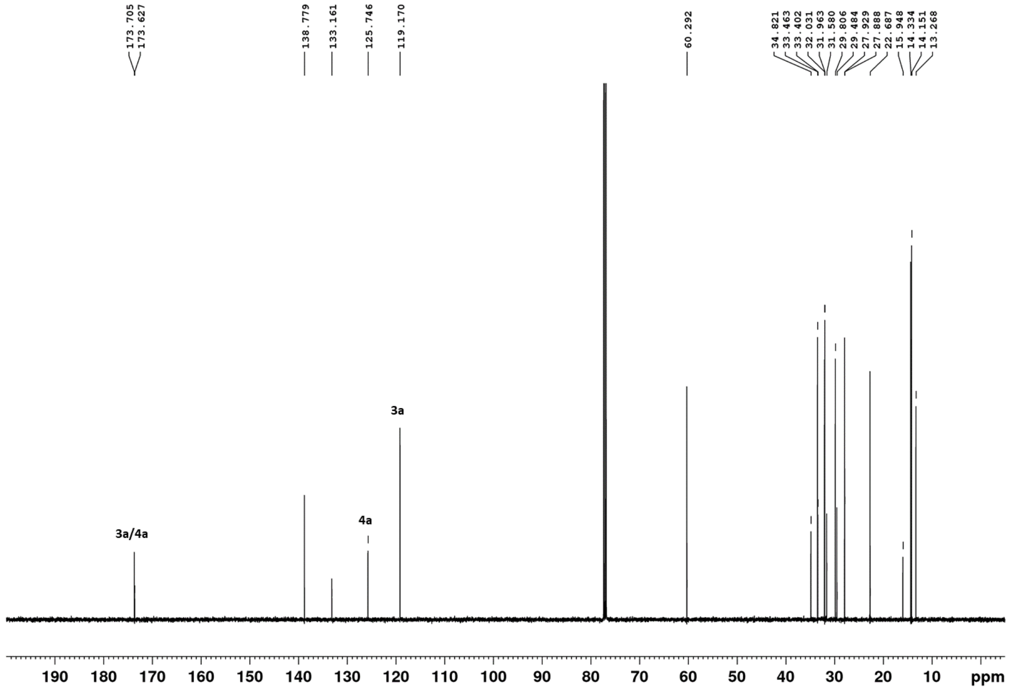

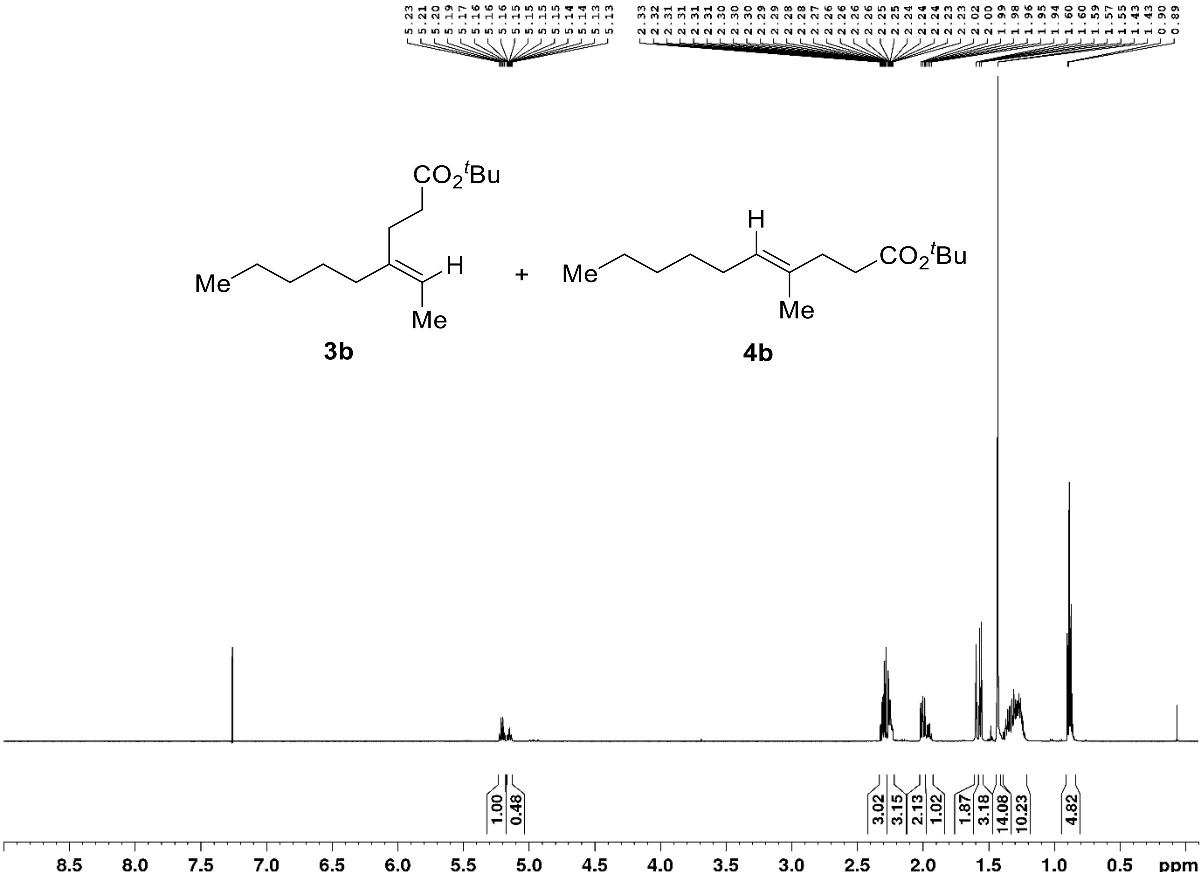
CO2tBu
Me
H
Me
+
Me
H
CO2tBu
Me
3b
4b
1.00
0.48
3.02
3.15
2.13
1.02
1.87
3.18
14.08
10.23
4.82
8.5 8.0 7.5 7.0 6.5 6.0 5.5 5.0 4.5 4.0 3.5 3.0 2.5 2.0 1.5 1.0 0.5 ppm

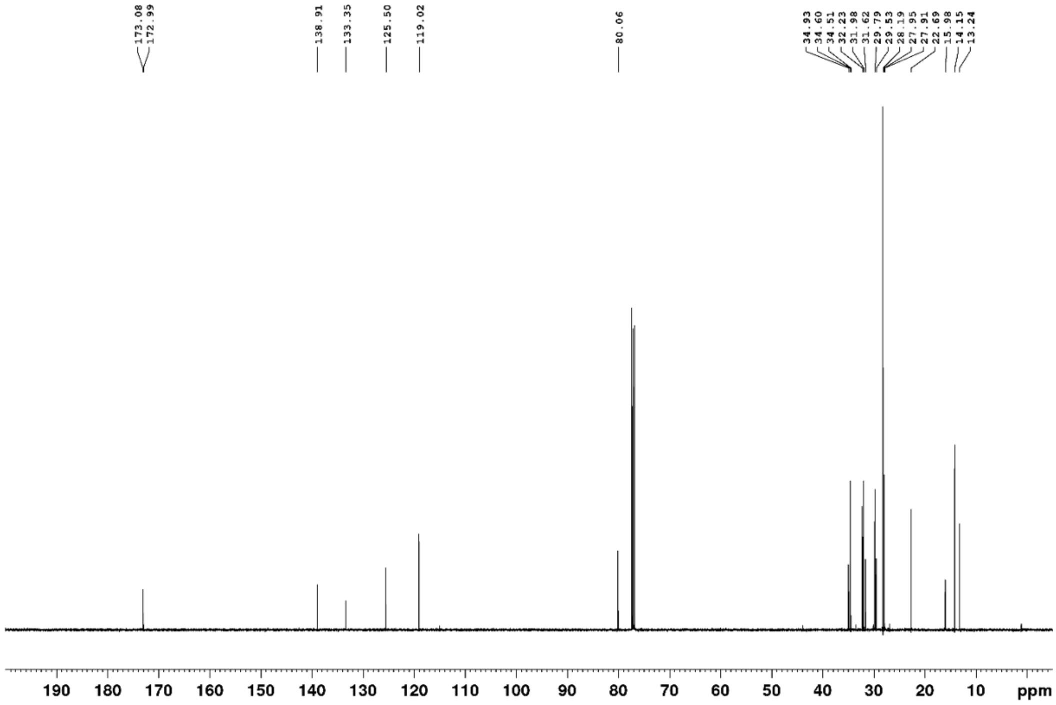
190 180 170 160 150 140 130 120 110 100 90 80 70 60 50 40 30 20 10 ppm

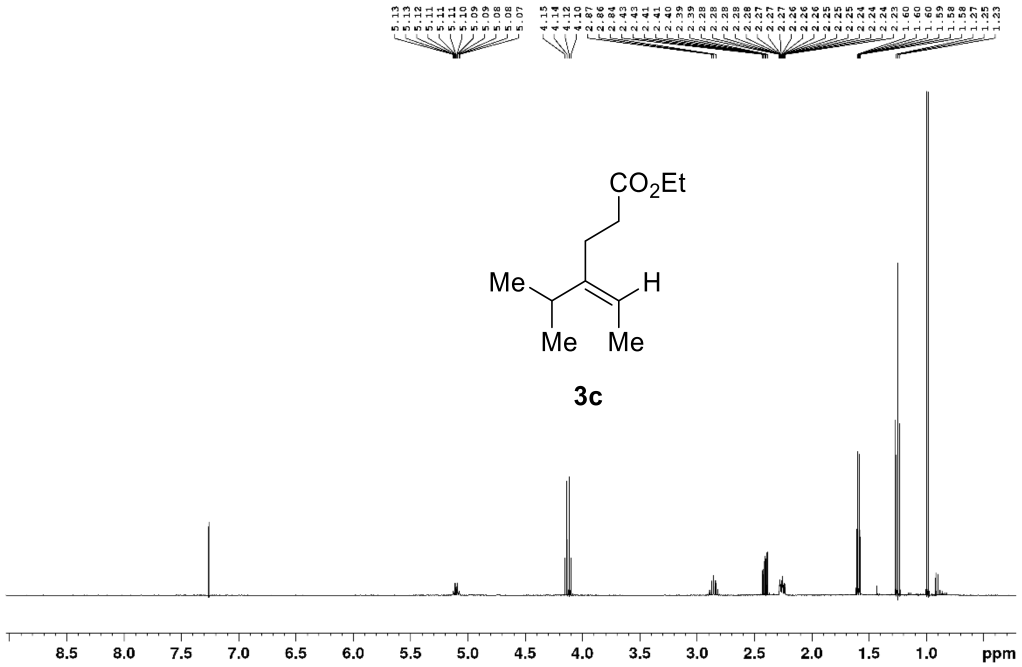
CO2Et
Me
H
Me
Me
3c
ppm

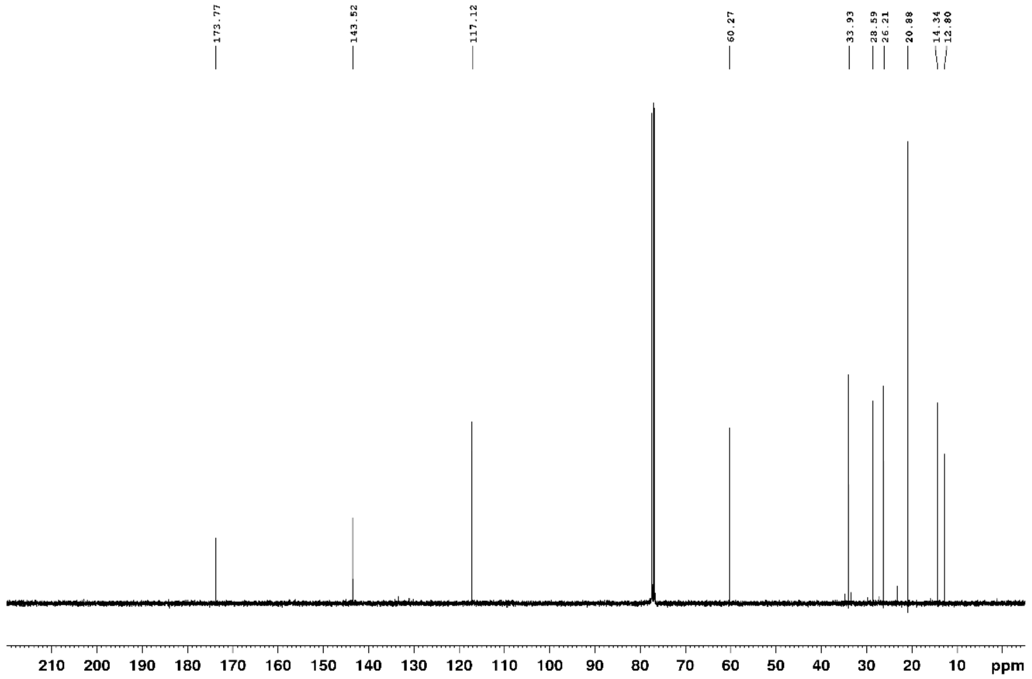
173.77
143.52
117.12
60.27
33.93
28.59
26.21
20.88
14.34
12.80
ppm

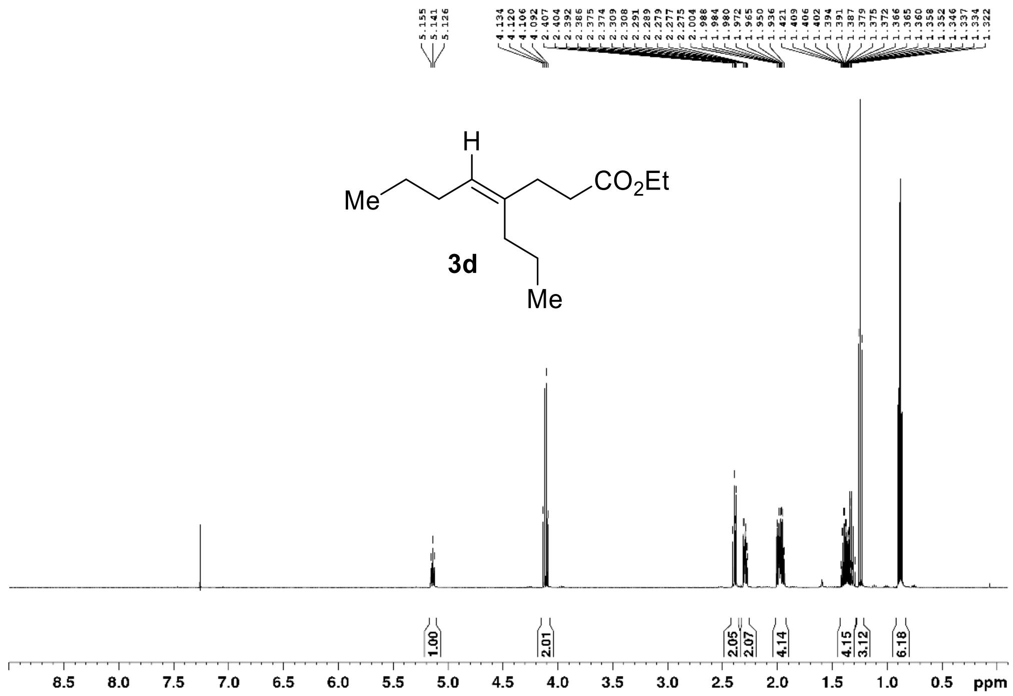
H
Me
CO2Et
3d
Me
1.00
2.01
2.05
2.07
4.14
4.15
3.12
6.18
8.5 8.0 7.5 7.0 6.5 6.0 5.5 5.0 4.5 4.0 3.5 3.0 2.5 2.0 1.5 1.0 0.5 ppm

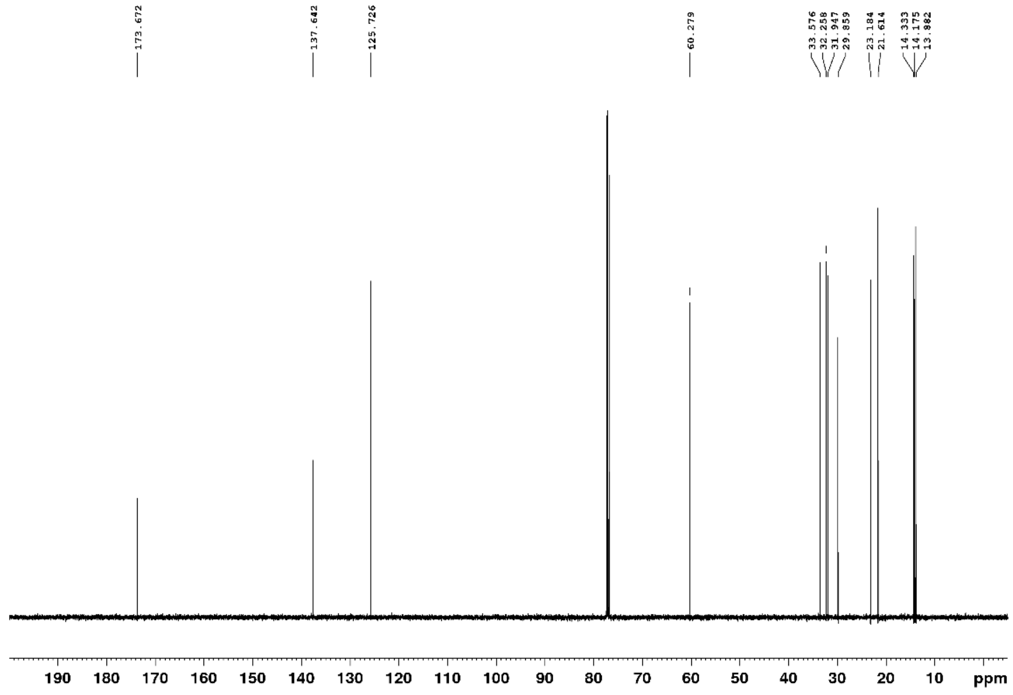
173.672
137.642
125.726
60.279
33.576
32.258
31.947
29.859
23.184
21.614
14.333
14.175
13.882
190 180 170 160 150 140 130 120 110 100 90 80 70 60 50 40 30 20 10 ppm

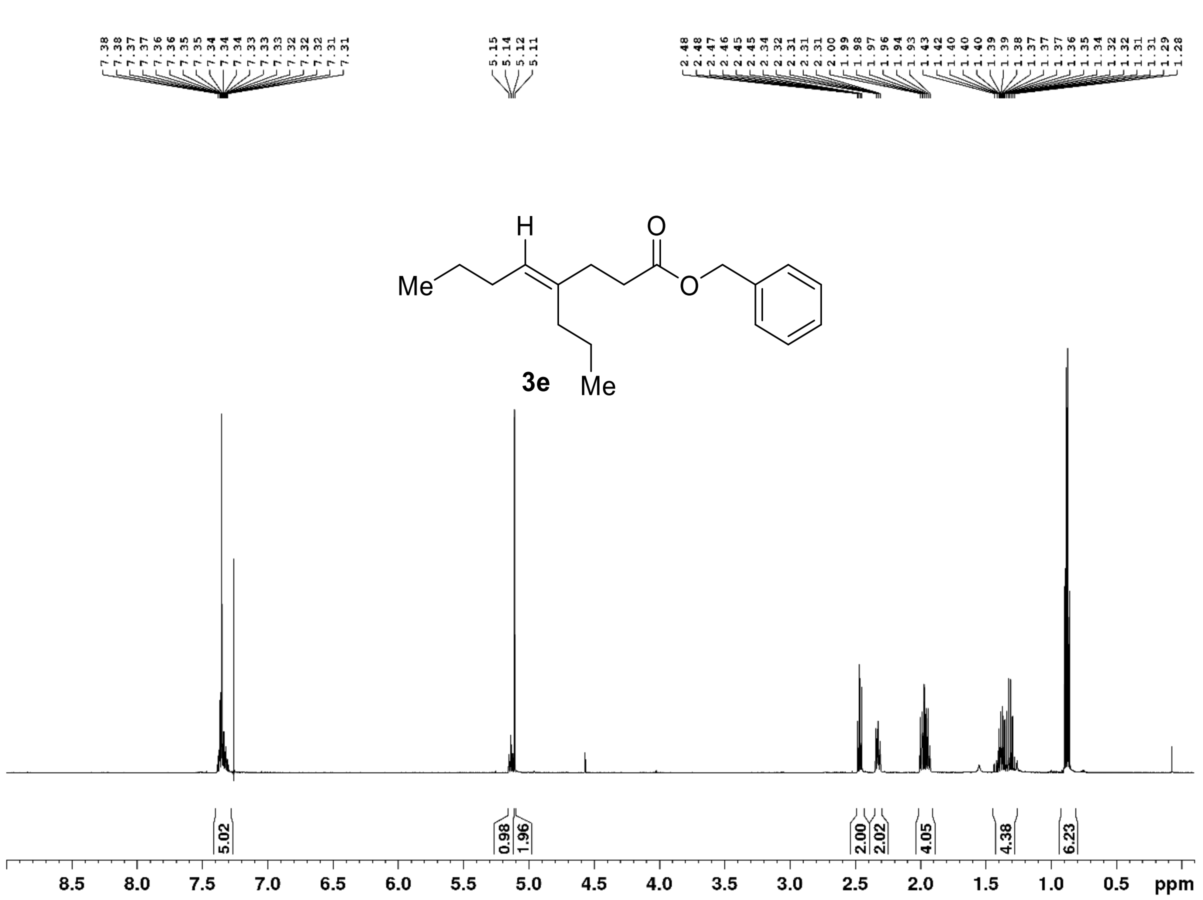
7.38 7.38 7.37 7.36 7.36 7.35 7.35 7.34 7.34 7.34 7.33 7.33 7.33 7.32 7.32 7.32 7.31 7.31
5.15 5.14 5.12 5.11
2.48 2.48 2.47 2.46 2.45 2.45 2.34 2.32 2.31 2.31 2.31 2.00 1.99 1.98 1.97 1.96 1.94 1.94 1.93 1.43 1.42 1.40 1.40 1.40 1.39 1.39 1.38 1.37 1.37 1.36 1.35 1.34 1.32 1.32 1.31 1.31 1.29 1.28
H
O
Me
O
3e
Me
5.02
0.98
1.96
2.00
2.02
4.05
4.38
6.23
8.5 8.0 7.5 7.0 6.5 6.0 5.5 5.0 4.5 4.0 3.5 3.0 2.5 2.0 1.5 1.0 0.5 ppm

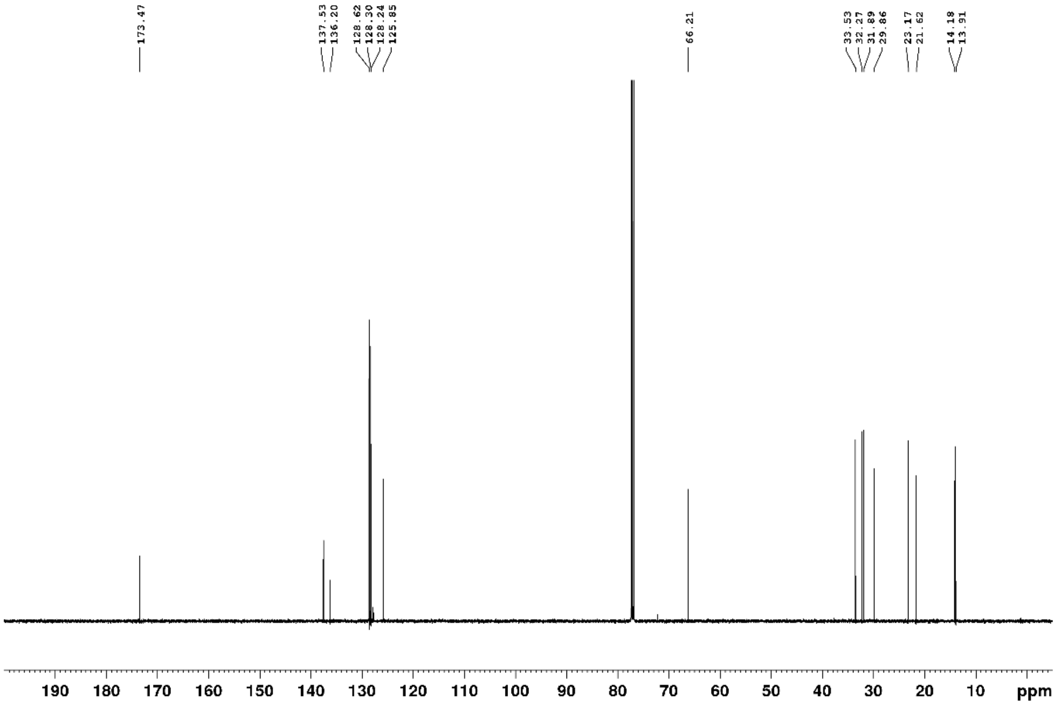
173.47
137.53 136.20 128.62 128.30 128.24 125.85
66.21
33.53 32.27 31.89 29.86 23.17 21.62 14.18 13.91
190 180 170 160 150 140 130 120 110 100 90 80 70 60 50 40 30 20 10 ppm

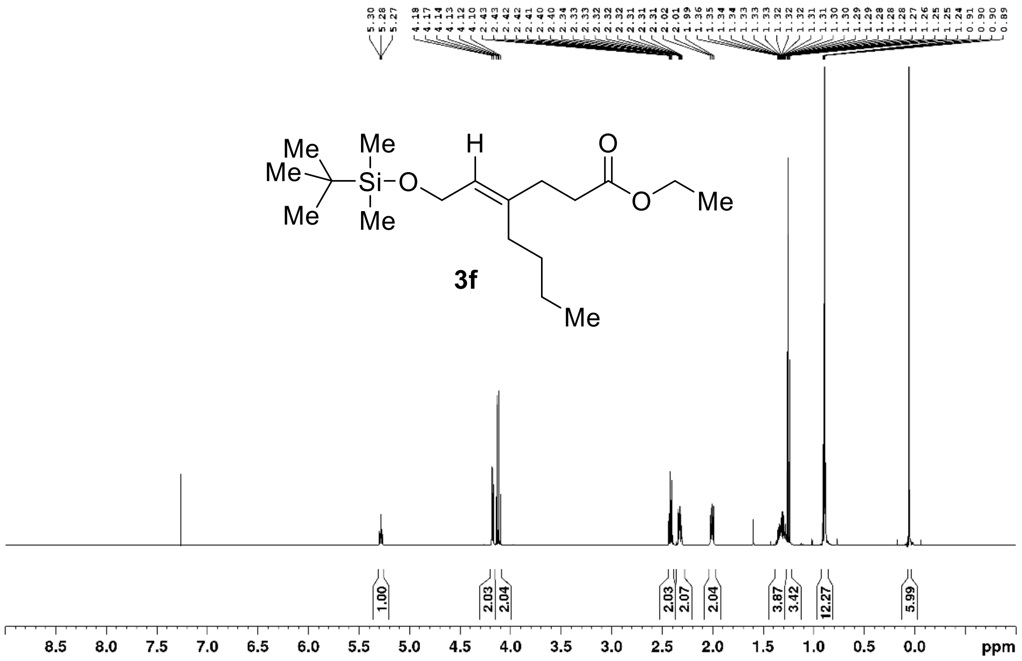
Me
Me
Me
Me
Si
O
Me
H
O
O
Me
3f
Me
1.00
2.03
2.04
2.03
2.07
2.04
3.87
3.42
12.27
5.99
8.5 8.0 7.5 7.0 6.5 6.0 5.5 5.0 4.5 4.0 3.5 3.0 2.5 2.0 1.5 1.0 0.5 0.0 ppm

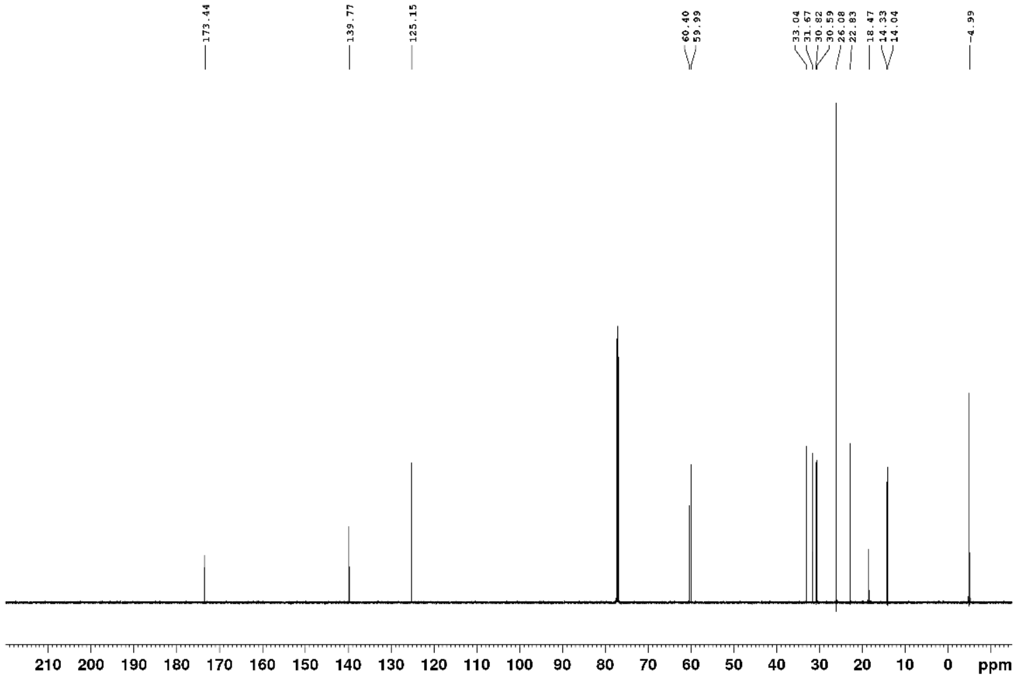
173.44
139.77
125.15
60.40
59.99
33.04
31.67
30.82
30.59
26.08
22.83
18.47
14.33
14.04
-4.99
210 200 190 180 170 160 150 140 130 120 110 100 90 80 70 60 50 40 30 20 10 0 ppm

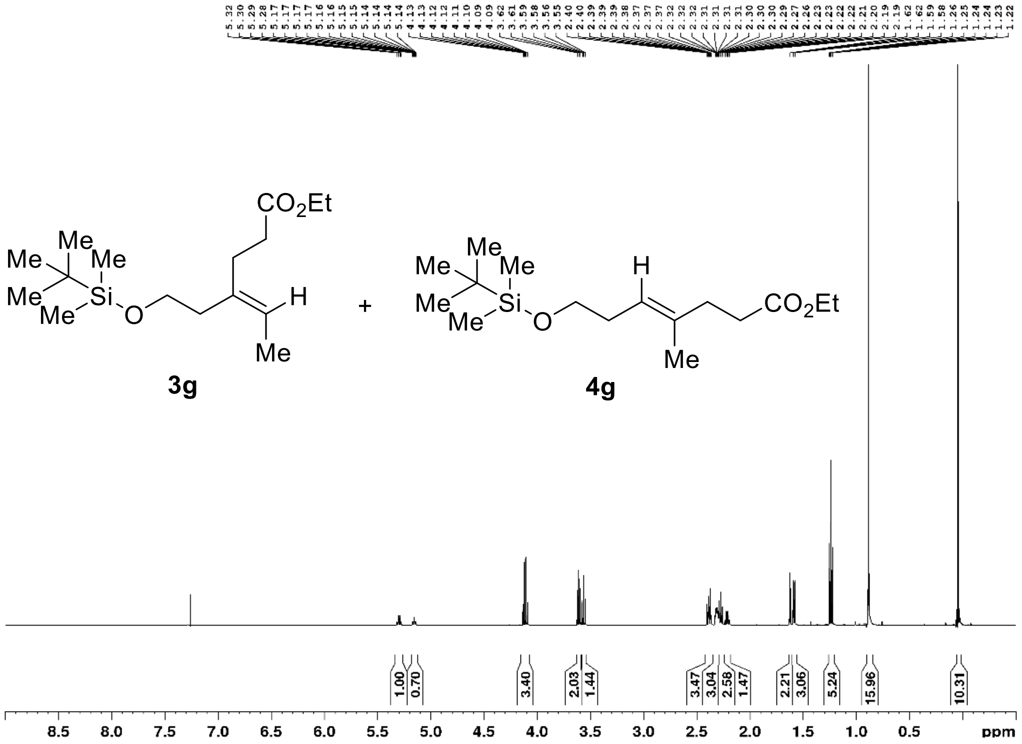
CO_2Et
Me
Me
Me
Me
Me
Si
O
H
Me
+
H
CO_2Et
3g
4g
1.00
0.70
3.40
2.03
1.44
3.47
3.04
2.58
1.47
2.21
3.06
5.24
15.96
10.31
8.5 8.0 7.5 7.0 6.5 6.0 5.5 5.0 4.5 4.0 3.5 3.0 2.5 2.0 1.5 1.0 0.5 ppm

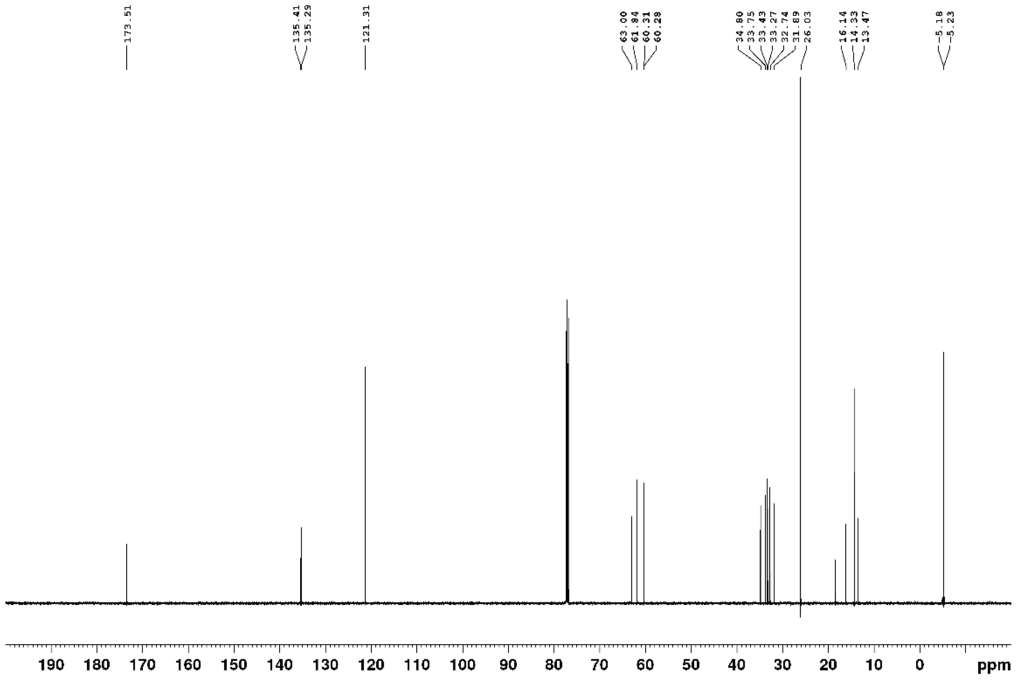
173.51
135.41
135.29
121.31
63.00
61.84
60.31
60.28
34.80
33.75
33.43
33.27
32.74
31.89
26.03
16.14
14.33
13.47
-5.18
-5.23
190 180 170 160 150 140 130 120 110 100 90 80 70 60 50 40 30 20 10 0 ppm

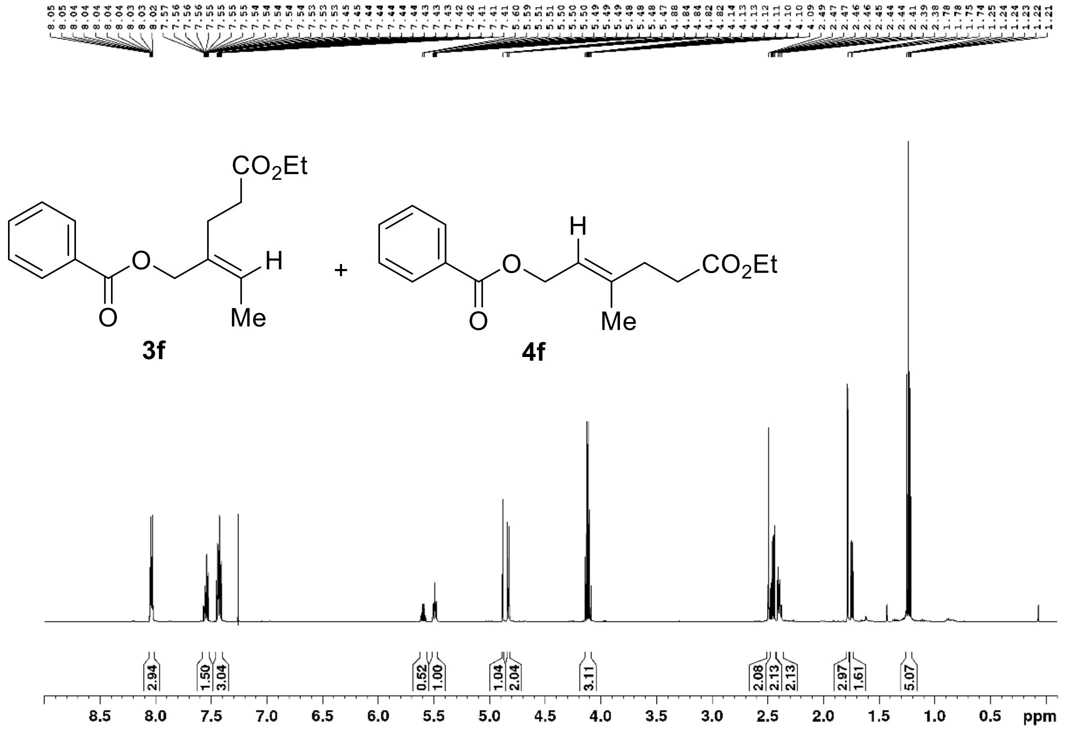
CO2Et
O
H
Me
+
3f
4f
ppm

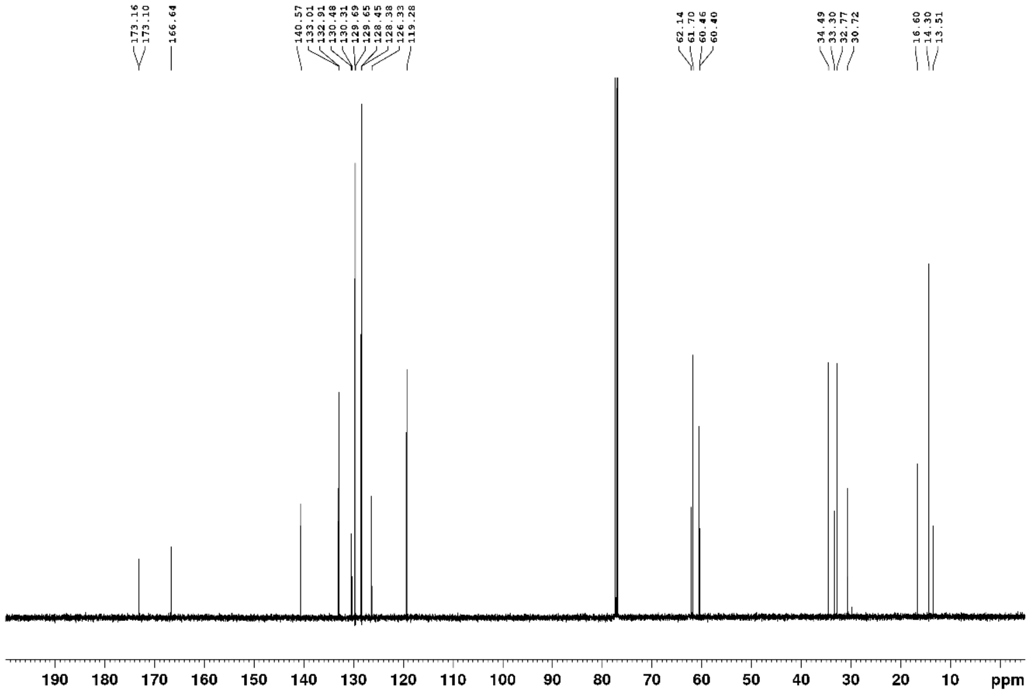
173.16
173.10
166.64
140.57
133.01
132.91
130.48
130.31
129.69
129.65
128.45
128.38
126.33
119.28
62.14
61.70
60.46
60.40
34.49
33.30
32.77
30.72
16.60
14.30
13.51
ppm

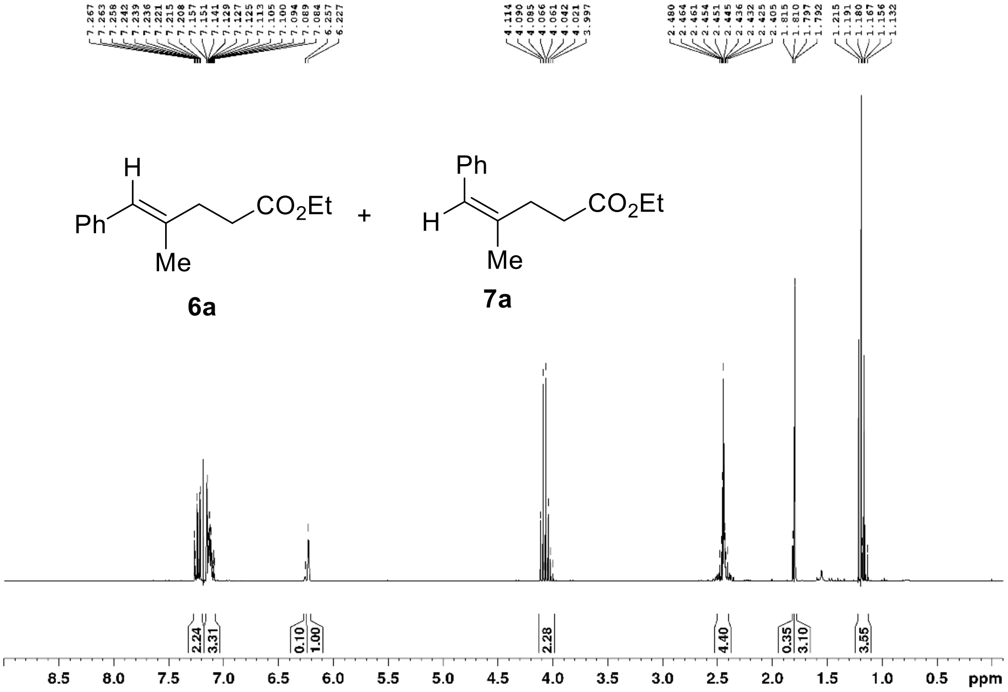
6a
7a
ppm

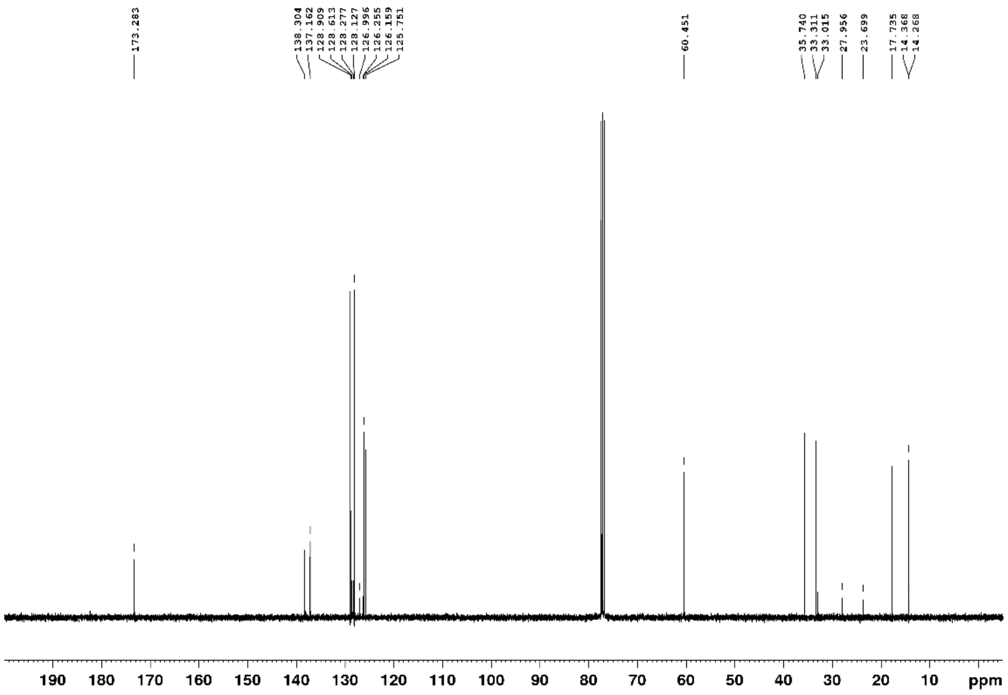
ppm

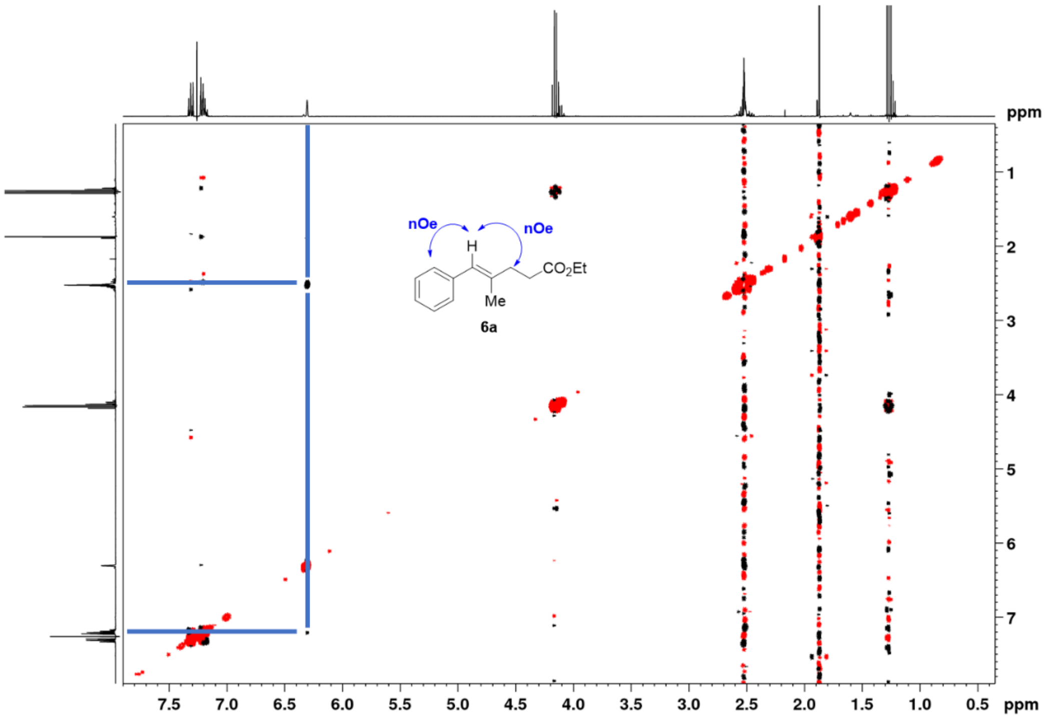
nOe
nOe
H
CO2Et
Me
6a
ppm
7.5
7.0
6.5
6.0
5.5
5.0
4.5
4.0
3.5
3.0
2.5
2.0
1.5
1.0
0.5
ppm
1
2
3
4
5
6
7

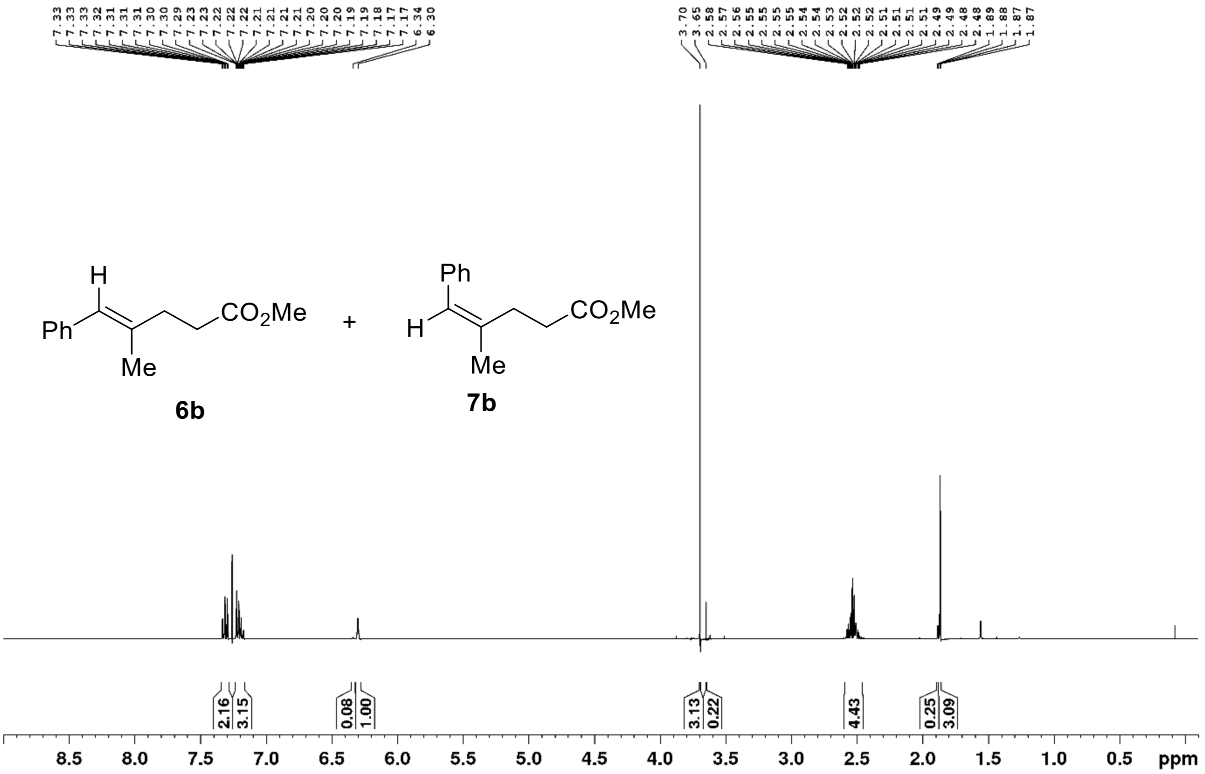
H
Ph
CO2Me
Me
6b
+
Ph
H
CO2Me
Me
7b
2.16
3.15
0.08
1.00
3.13
0.22
4.43
0.25
3.09
8.5
8.0
7.5
7.0
6.5
6.0
5.5
5.0
4.5
4.0
3.5
3.0
2.5
2.0
1.5
1.0
0.5
ppm

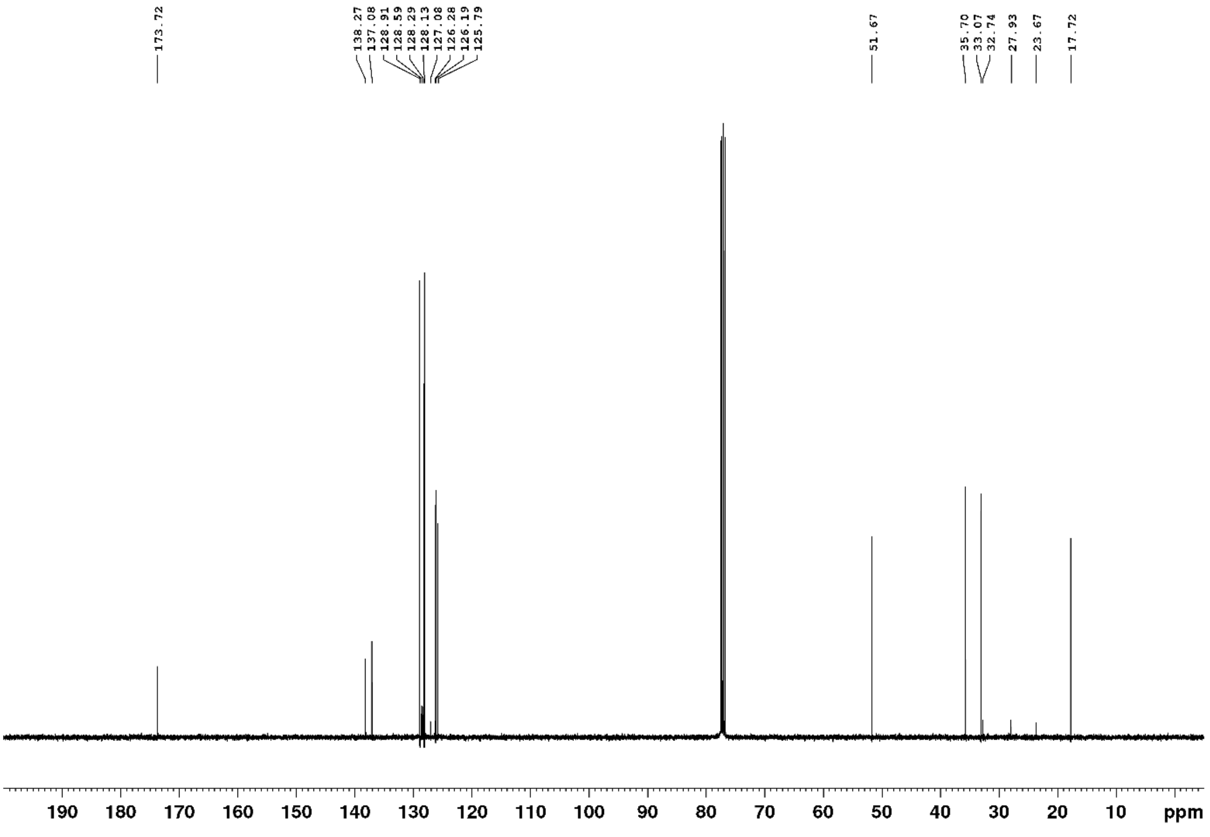
173.72
138.27
137.08
128.91
128.59
128.29
128.13
127.08
126.28
126.19
125.79
51.67
35.70
33.07
32.74
27.93
23.67
17.72
190
180
170
160
150
140
130
120
110
100
90
80
70
60
50
40
30
20
10
ppm

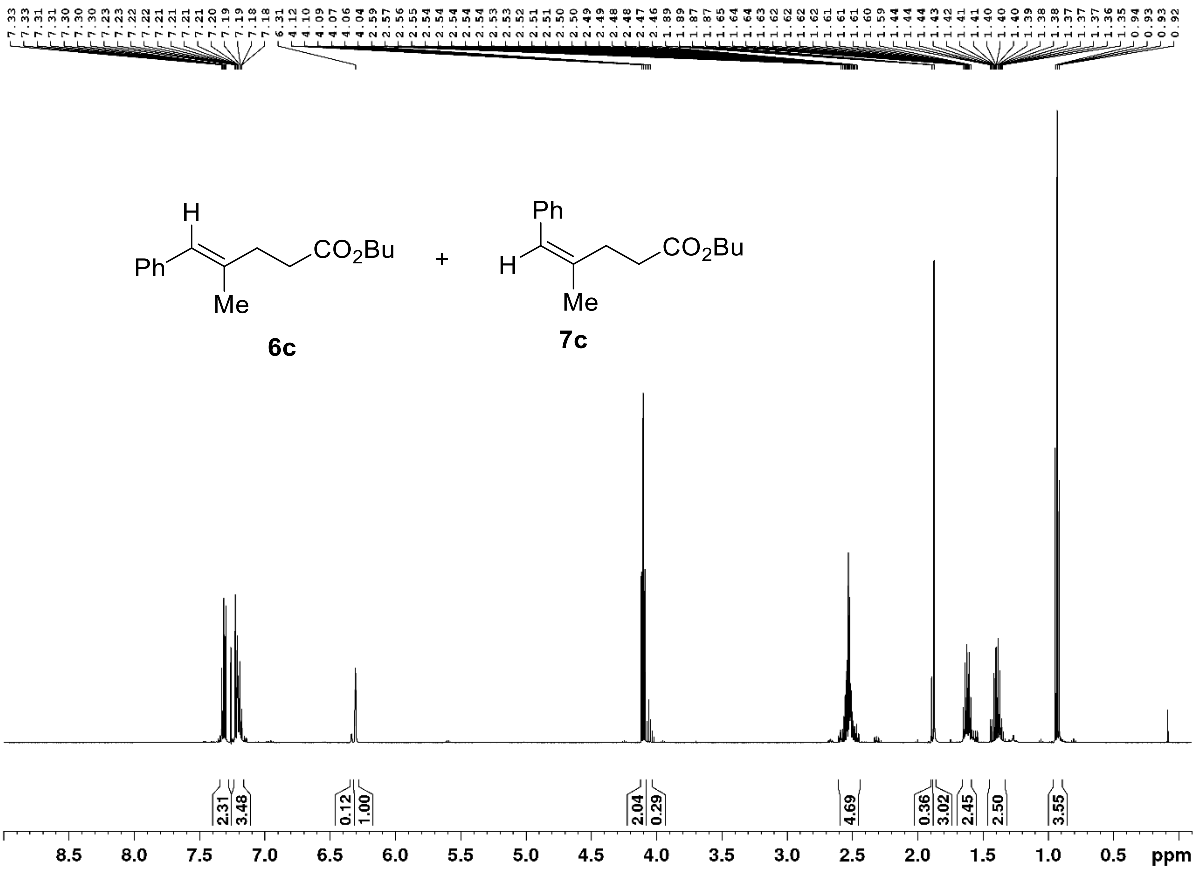
H
Ph
CO2Bu
Me
6c
+
Ph
H
CO2Bu
Me
7c
2.31
3.48
0.12
1.00
2.04
0.29
4.69
0.36
3.02
2.45
2.50
3.55
8.5
8.0
7.5
7.0
6.5
6.0
5.5
5.0
4.5
4.0
3.5
3.0
2.5
2.0
1.5
1.0
0.5
ppm

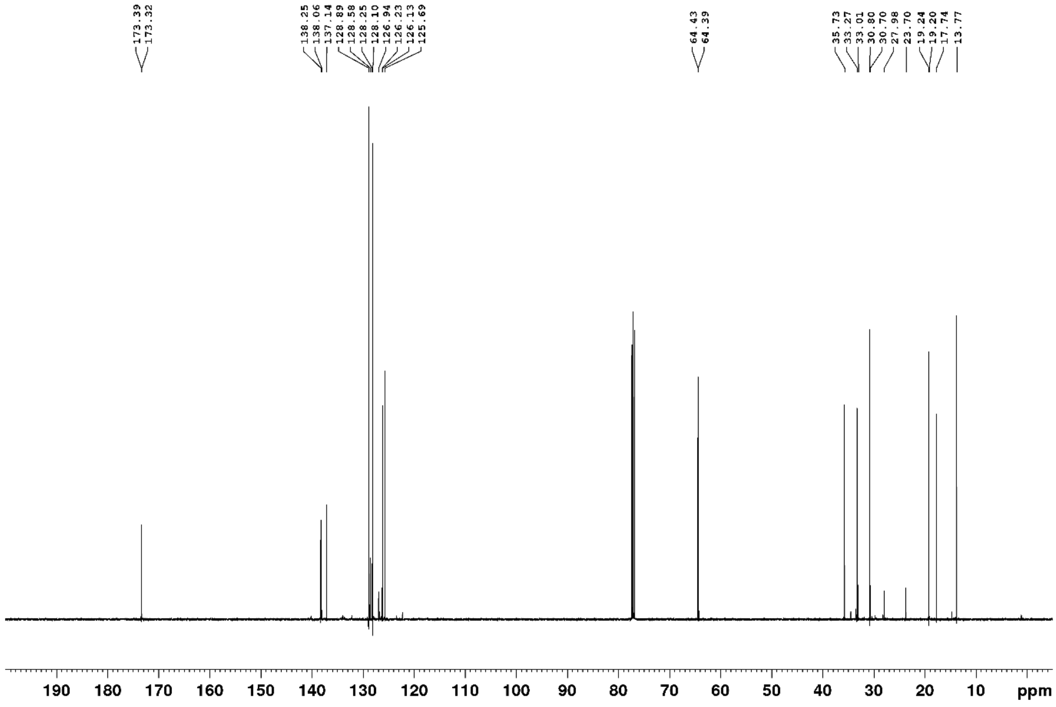
173.39
173.32
138.25
138.06
137.14
128.89
128.58
128.25
128.10
126.94
126.23
126.13
125.69
64.43
64.39
35.73
33.27
33.01
30.80
30.70
27.98
23.70
19.24
19.20
17.74
13.77
190
180
170
160
150
140
130
120
110
100
90
80
70
60
50
40
30
20
10
ppm

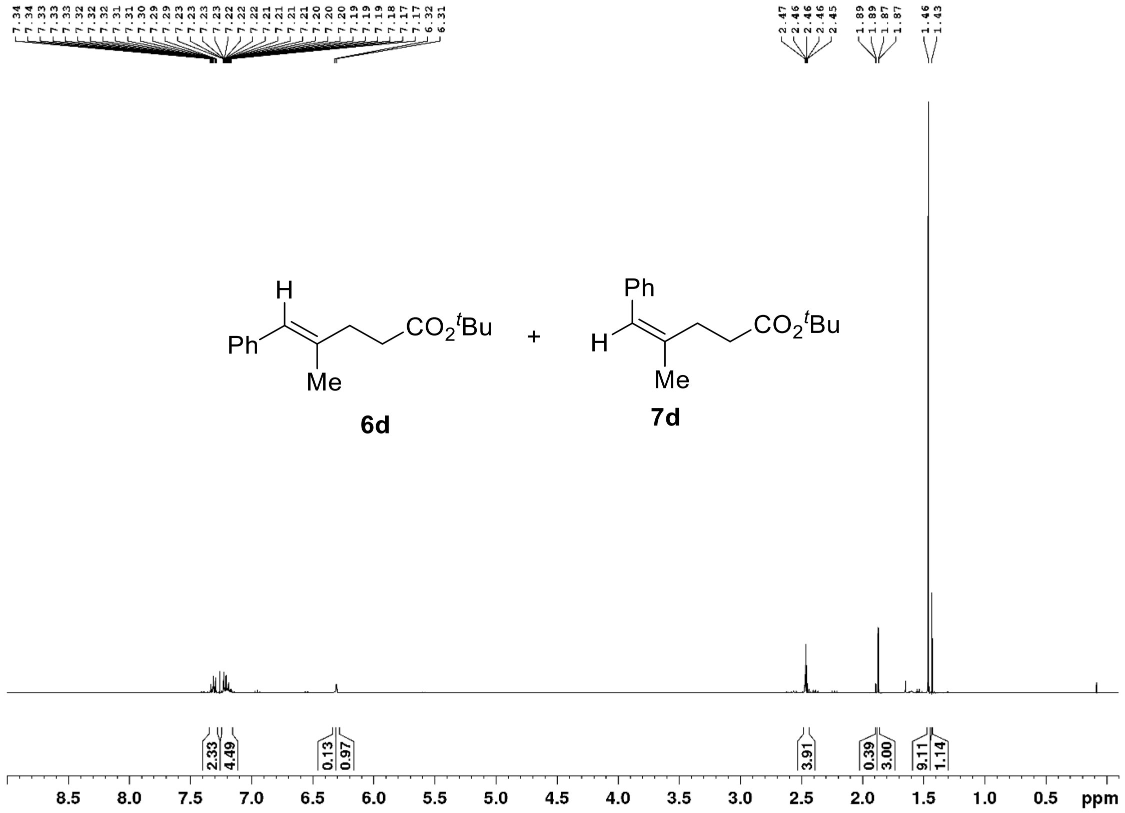
H
Ph
CO2tBu
Me
6d
+
Ph
H
CO2tBu
Me
7d
2.33
4.49
0.13
0.97
3.91
0.39
3.00
9.11
1.14
8.5
8.0
7.5
7.0
6.5
6.0
5.5
5.0
4.5
4.0
3.5
3.0
2.5
2.0
1.5
1.0
0.5
ppm

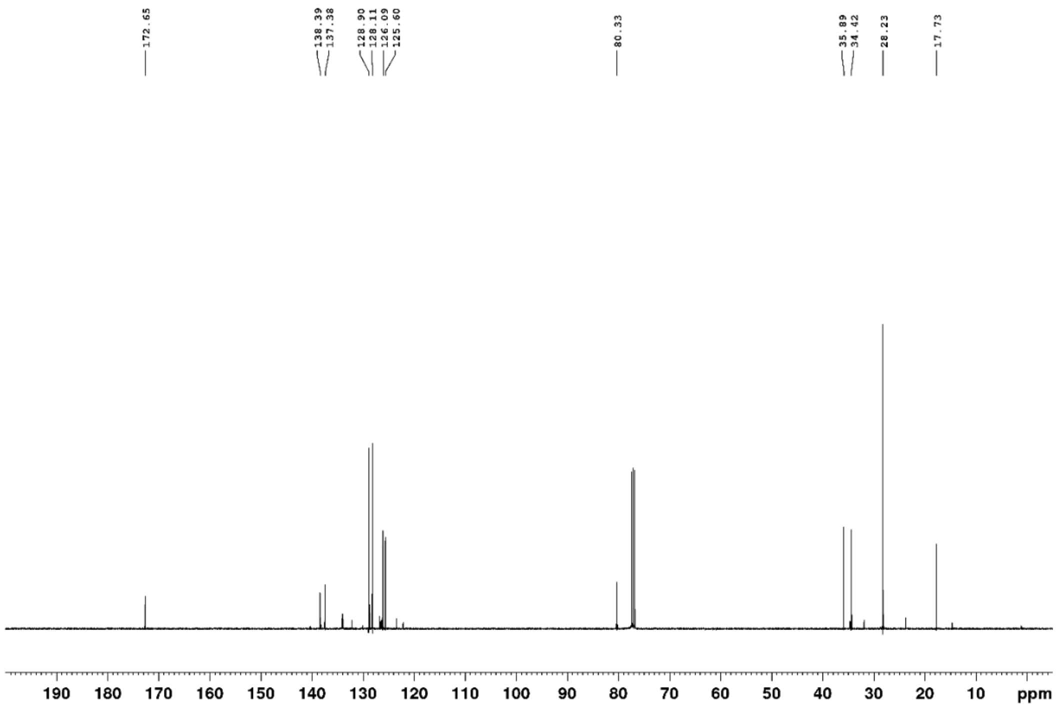
172.65
138.39
137.38
128.90
128.11
126.09
125.60
80.33
35.89
34.42
28.23
17.73
190
180
170
160
150
140
130
120
110
100
90
80
70
60
50
40
30
20
10
ppm

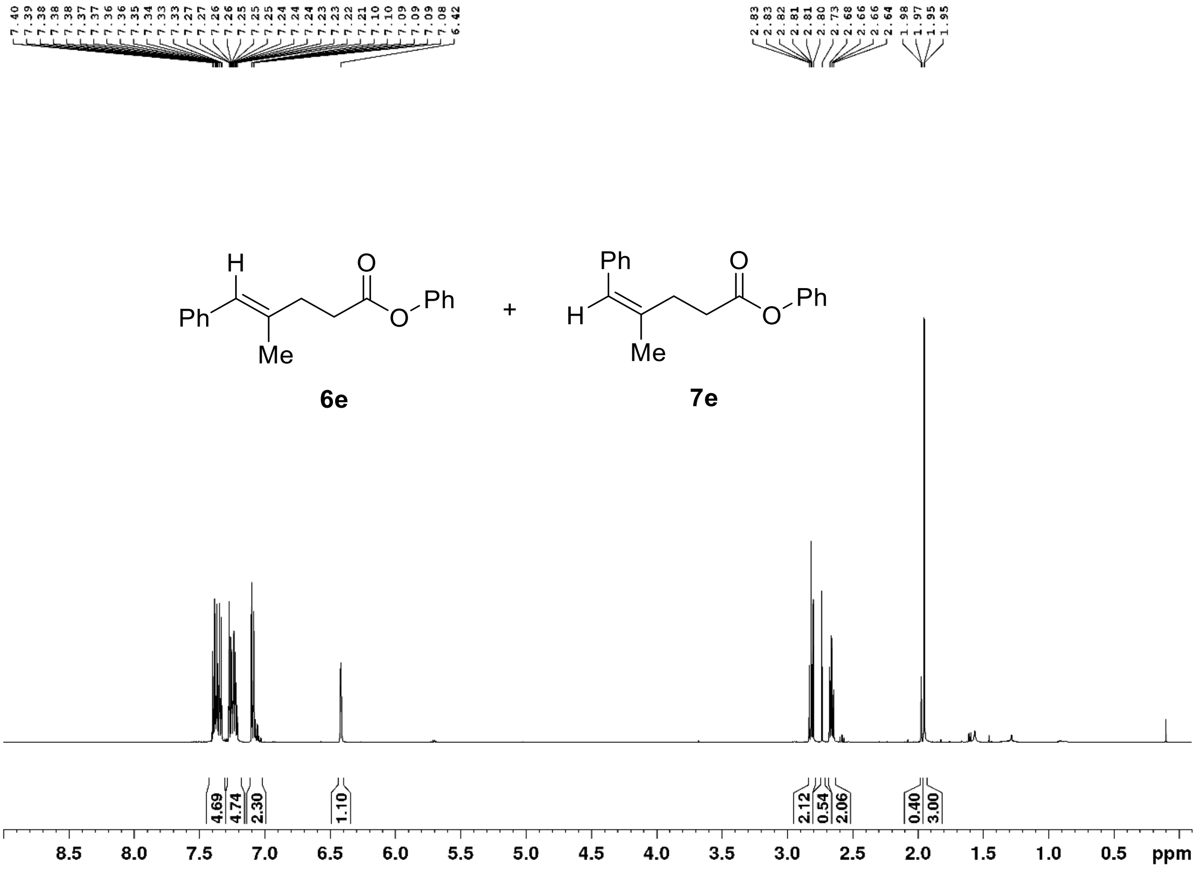
6e
7e

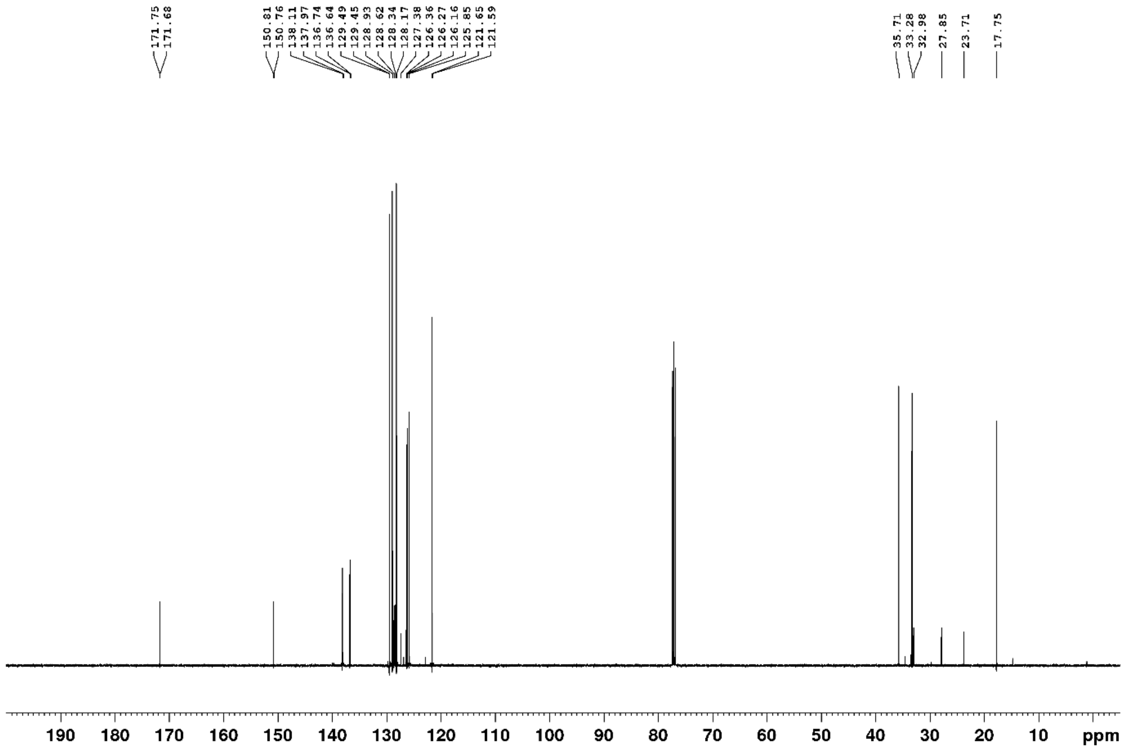

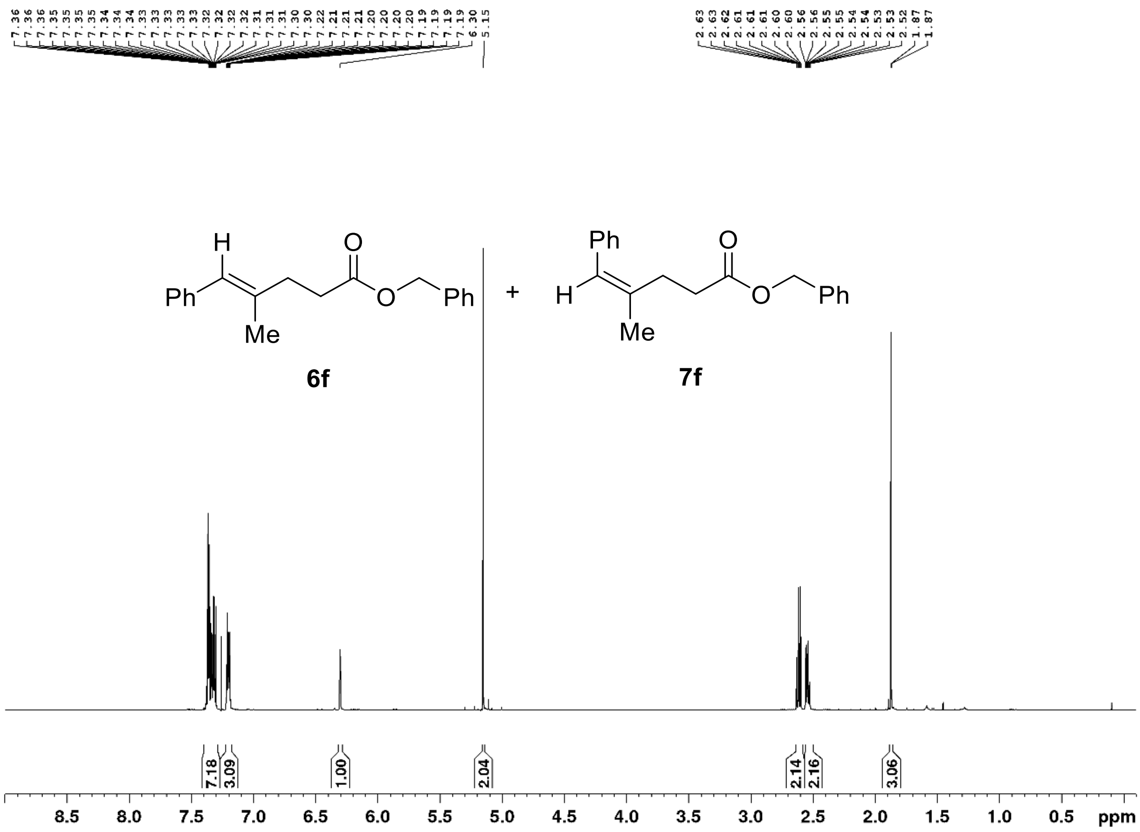
7.36 7.36 7.35 7.35 7.35 7.34 7.34 7.33 7.33 7.33 7.33 7.32 7.32 7.32 7.31 7.31 7.30 7.30 7.22 7.21 7.21 7.21 7.20 7.20 7.20 7.19 7.19 7.19 6.30 5.15
2.63 2.63 2.62 2.61 2.61 2.60 2.60 2.56 2.56 2.55 2.55 2.54 2.54 2.53 2.53 2.52 1.87 1.87
H
O
Ph
O
Ph
Me
6f
+
Ph
O
H
O
Ph
Me
7f
7.18 3.09 1.00 2.04 2.14 2.16 3.06
8.5 8.0 7.5 7.0 6.5 6.0 5.5 5.0 4.5 4.0 3.5 3.0 2.5 2.0 1.5 1.0 0.5 ppm

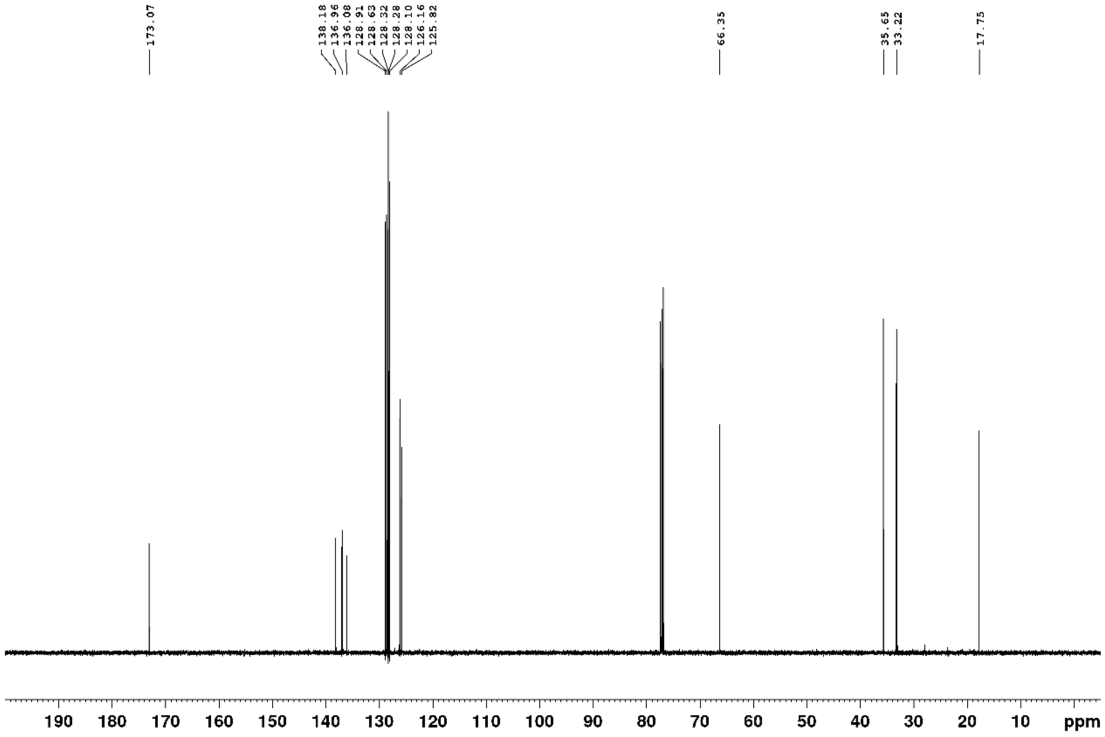
173.07
138.18 136.96 136.08 128.91 128.63 128.32 128.28 128.10 126.16 125.82
66.35
35.65 33.22
17.75
190 180 170 160 150 140 130 120 110 100 90 80 70 60 50 40 30 20 10 ppm

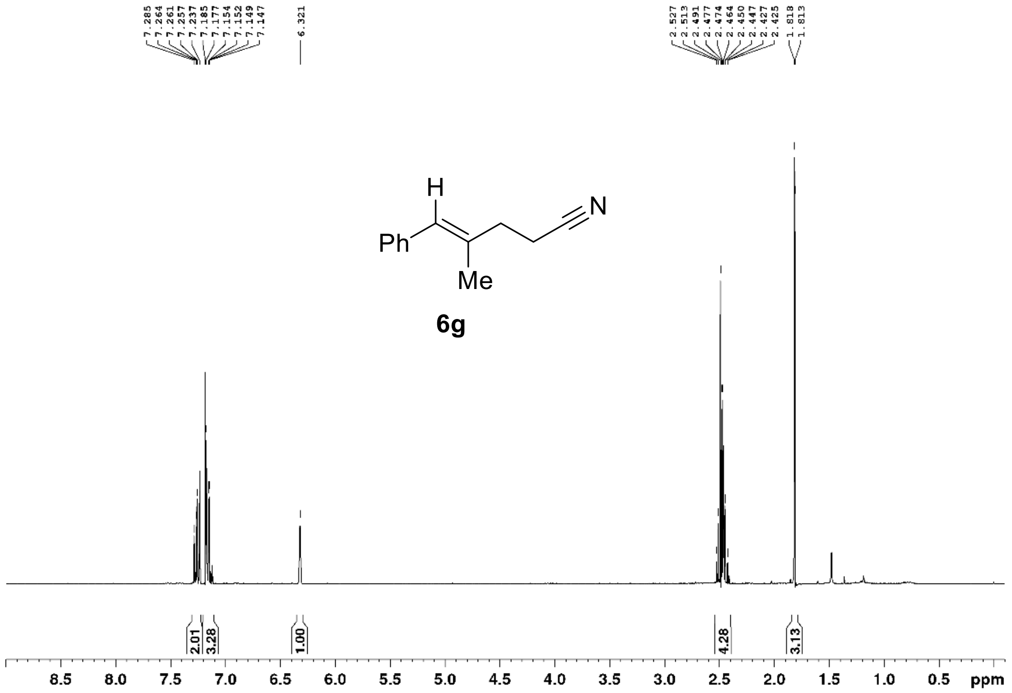
H
N
Ph
Me
6g

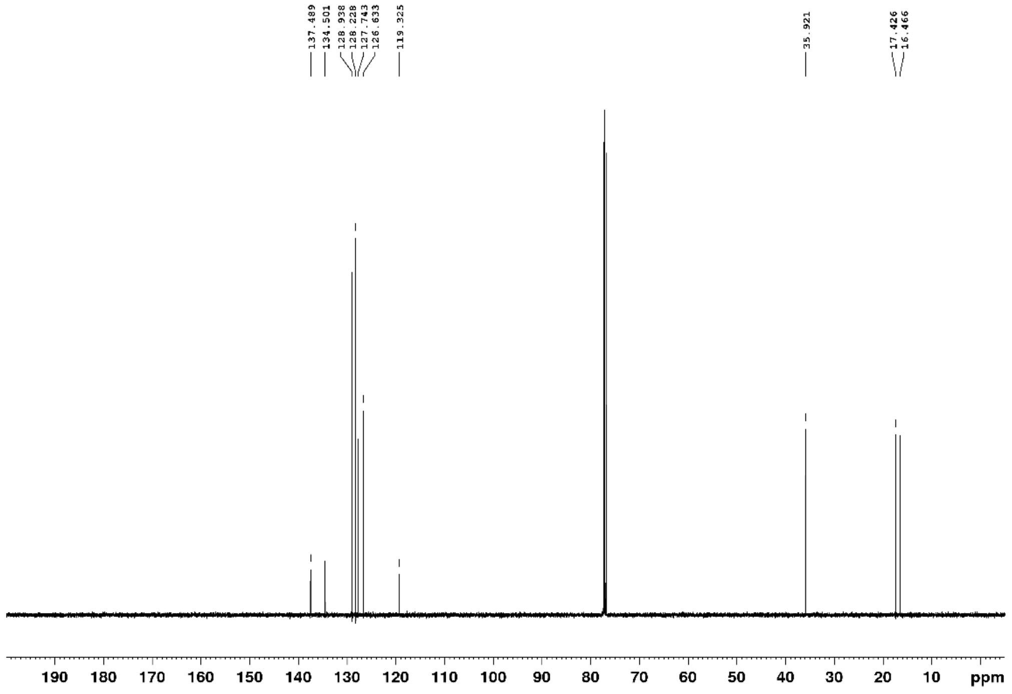

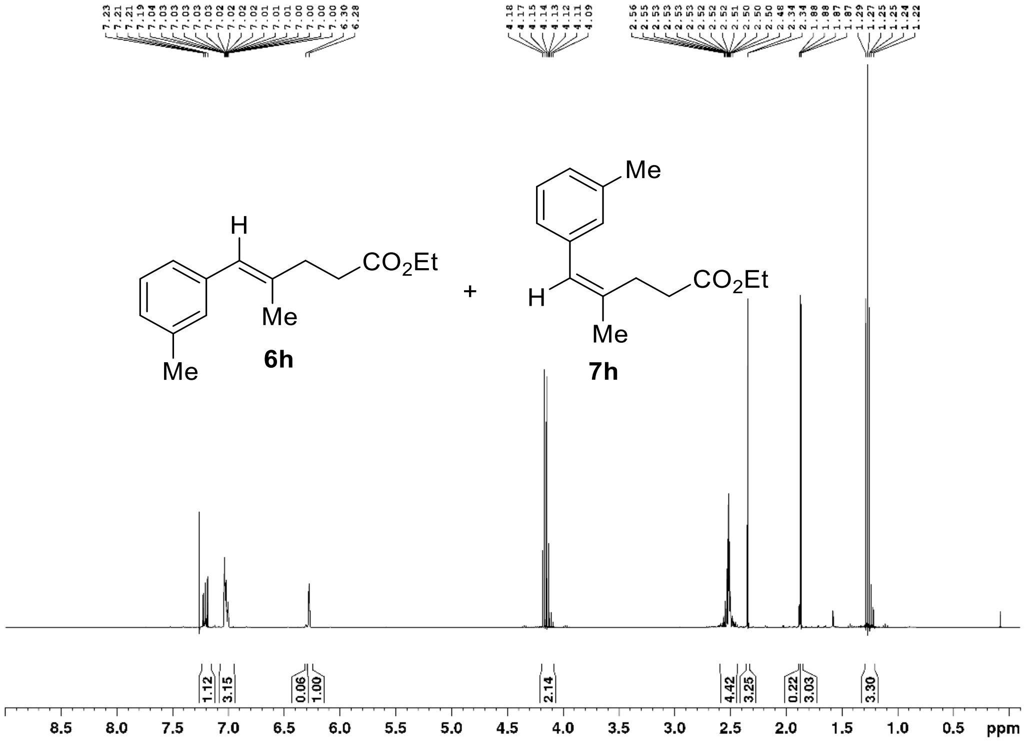
Me
H
CO_2Et
+
H
CO_2Et
Me
Me
Me
6h
7h
8.5 8.0 7.5 7.0 6.5 6.0 5.5 5.0 4.5 4.0 3.5 3.0 2.5 2.0 1.5 1.0 0.5 ppm

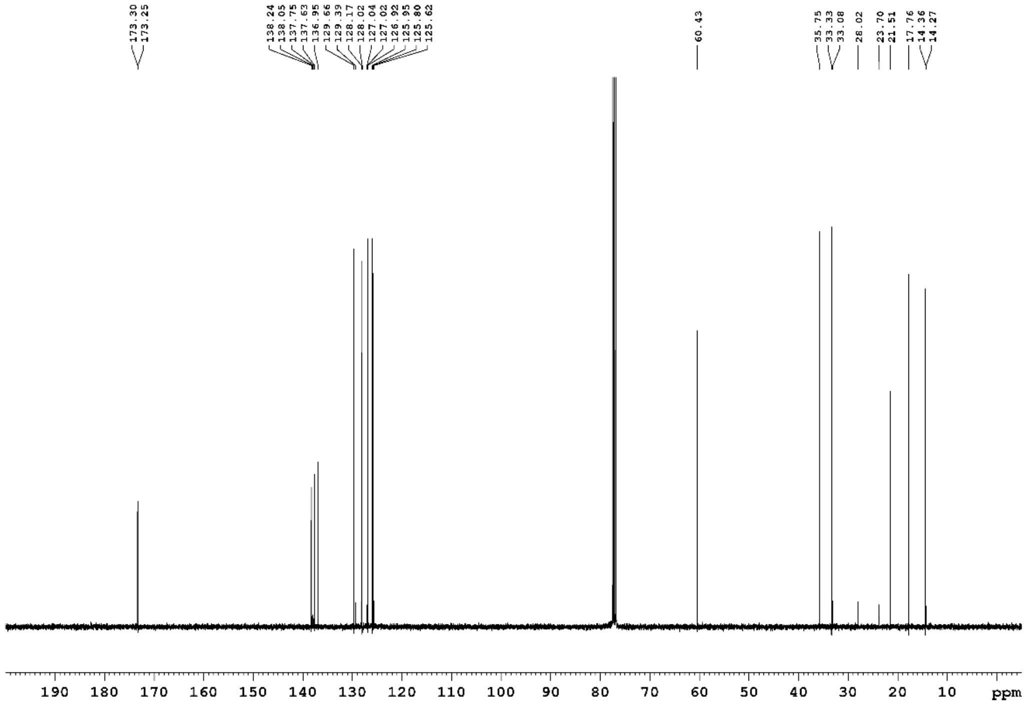
190 180 170 160 150 140 130 120 110 100 90 80 70 60 50 40 30 20 10 ppm

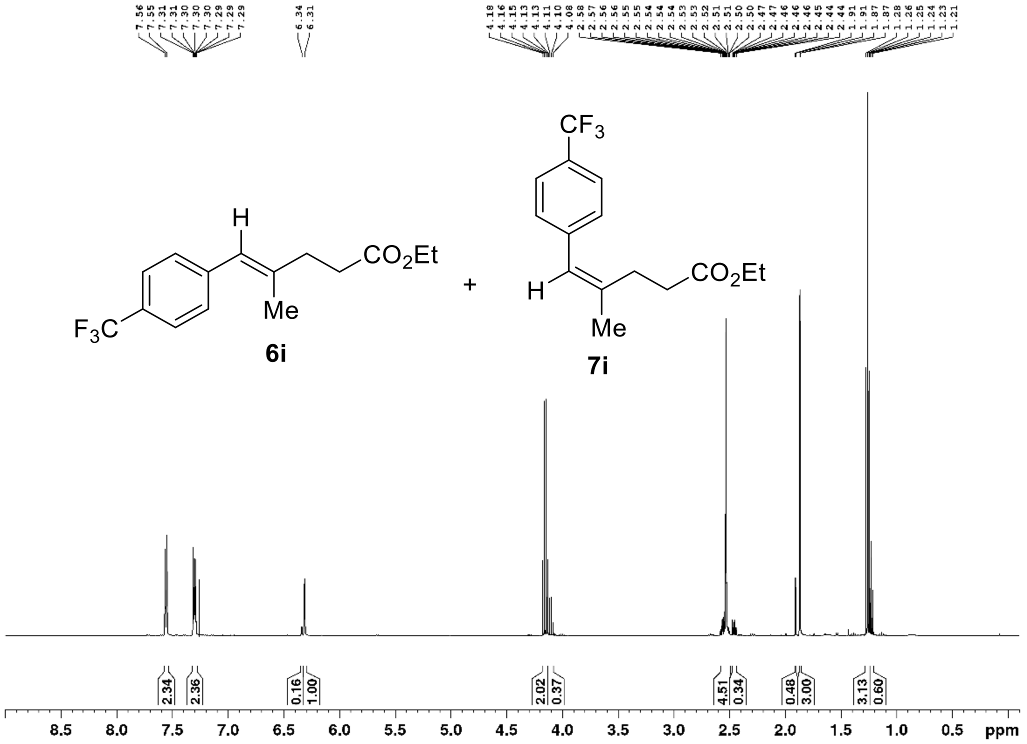
CF3
H
CO2Et
Me
F3C
6i
+
H
CO2Et
Me
7i
ppm

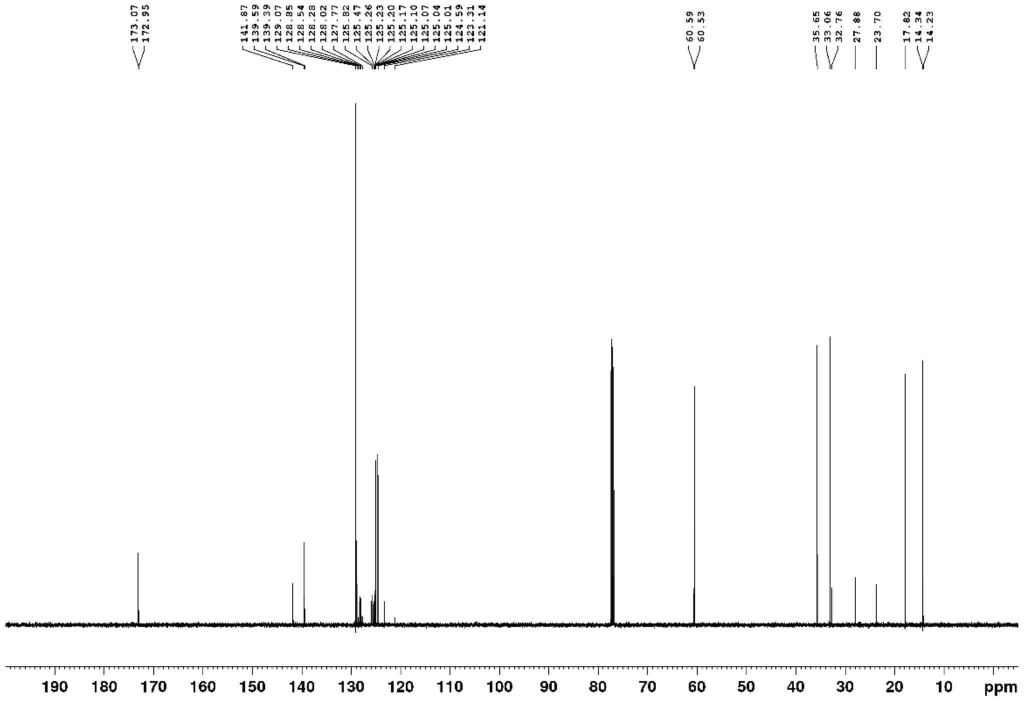
ppm

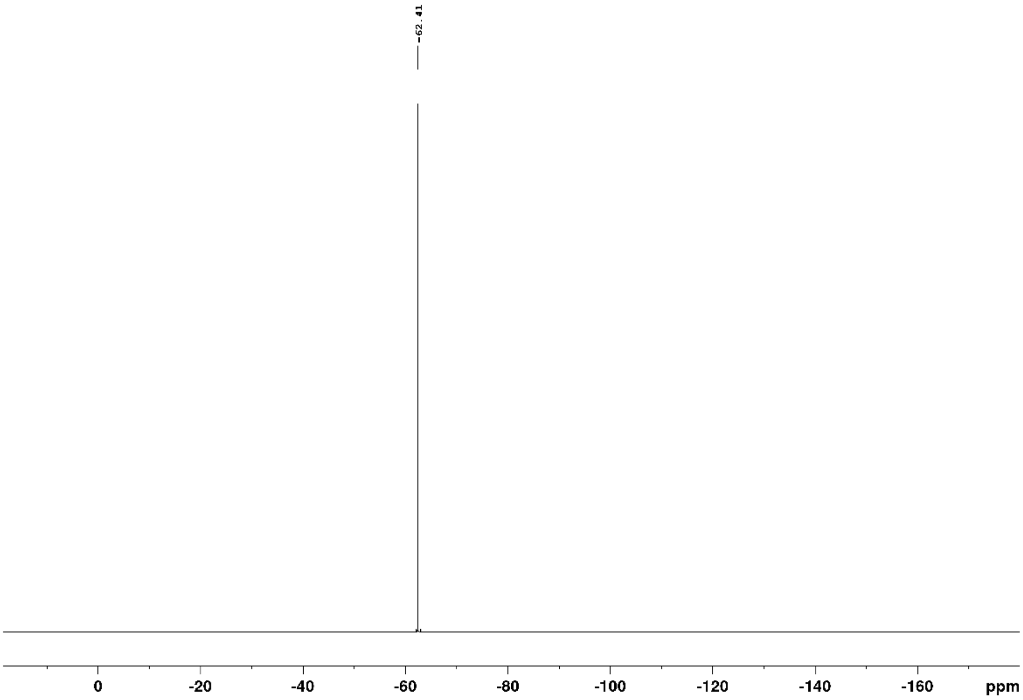
-62.41
0
-20
-40
-60
-80
-100
-120
-140
-160
ppm

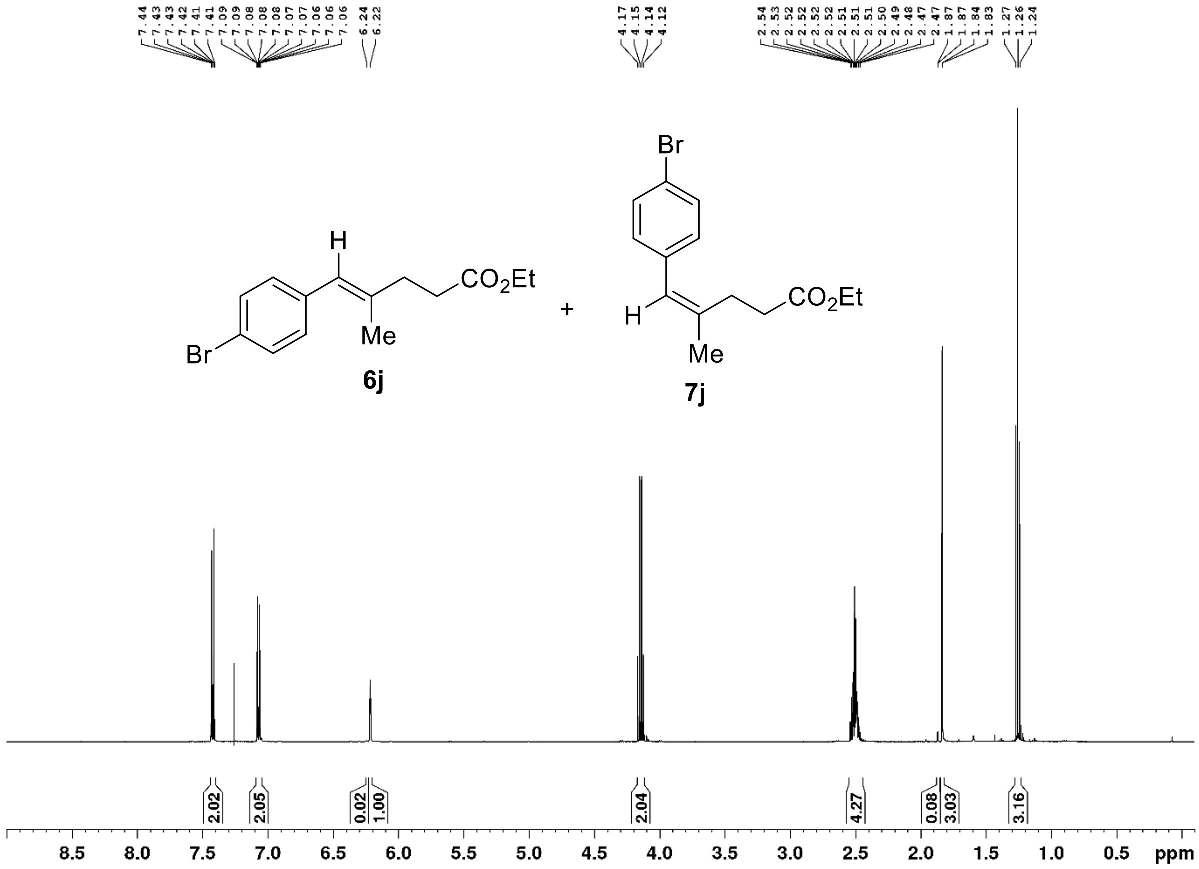
7.44 7.43 7.43 7.42 7.41 7.41 7.09 7.09 7.08 7.08 7.07 7.07 7.06 7.06 7.06 6.24 6.22
4.17 4.15 4.14 4.12
2.54 2.53 2.52 2.52 2.52 2.52 2.51 2.51 2.51 2.50 2.49 2.48 2.47 2.47 1.87 1.87 1.84 1.83 1.27 1.26 1.24
Br
H
CO2Et
Me
6j
+
7j
2.02 2.05 0.02 1.00 2.04 4.27 0.08 3.03 3.16
8.5 8.0 7.5 7.0 6.5 6.0 5.5 5.0 4.5 4.0 3.5 3.0 2.5 2.0 1.5 1.0 0.5 ppm

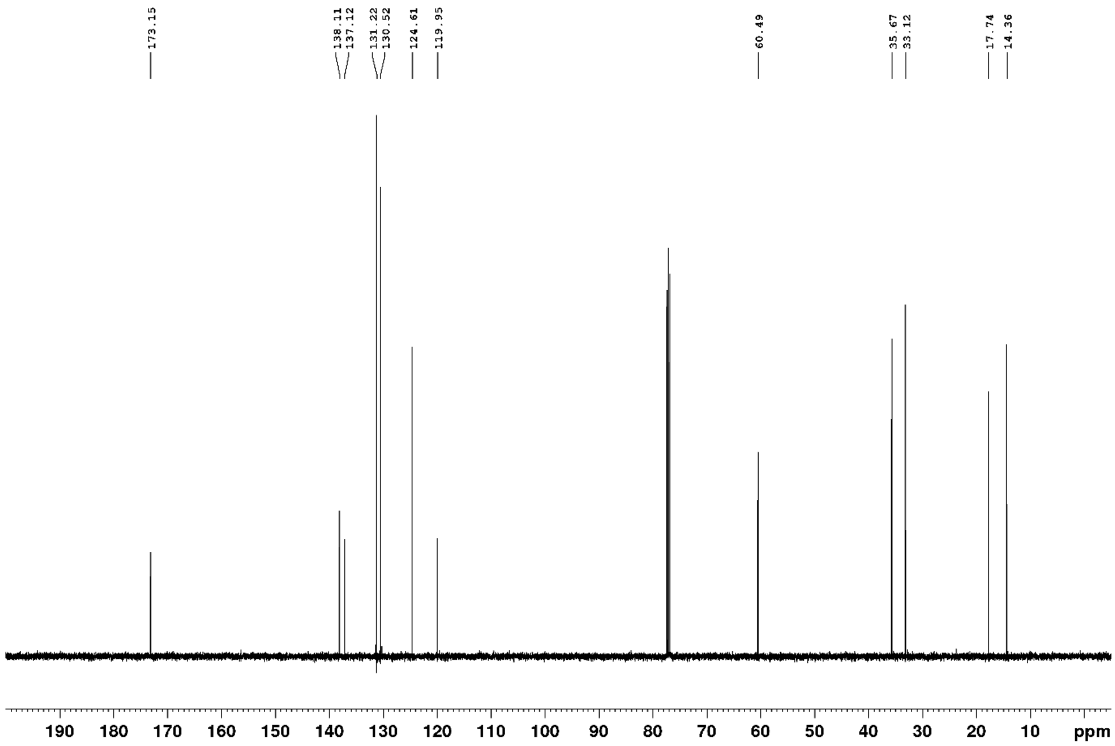
173.15
138.11 137.12 131.22 130.52 124.61 119.95
60.49
35.67 33.12
17.74 14.36
190 180 170 160 150 140 130 120 110 100 90 80 70 60 50 40 30 20 10 ppm

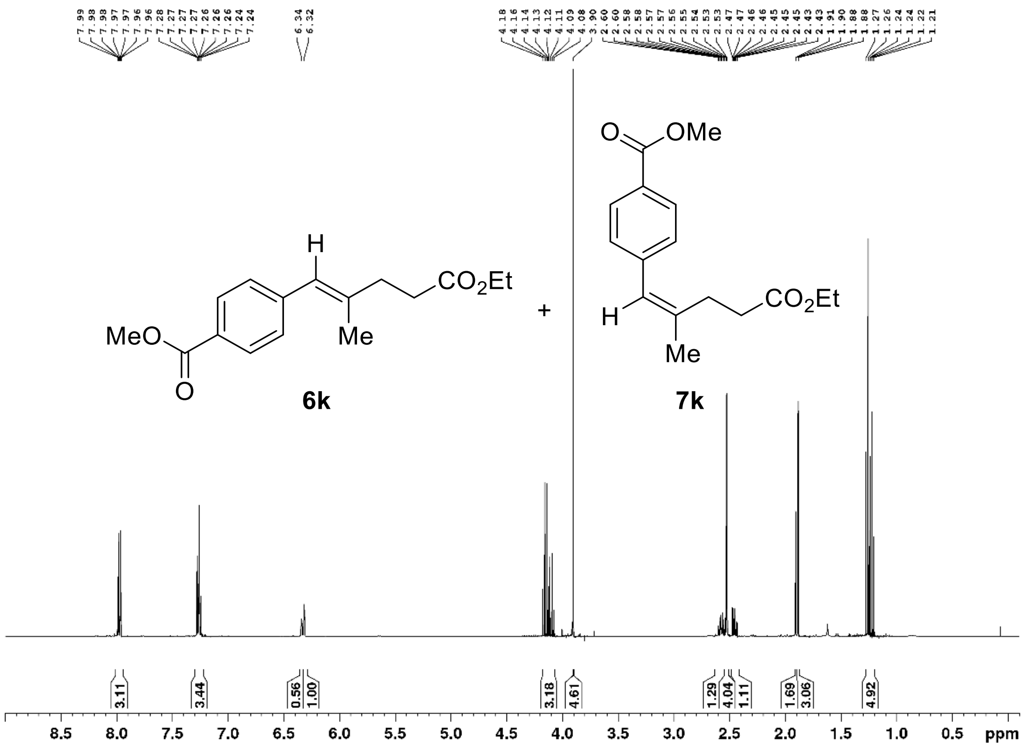
H
CO2Et
MeO
Me
O
6k
+
OMe
H
CO2Et
Me
7k
3.11
3.44
0.56
1.00
3.18
4.61
1.29
4.04
1.11
1.69
3.06
4.92
8.5
8.0
7.5
7.0
6.5
6.0
5.5
5.0
4.5
4.0
3.5
3.0
2.5
2.0
1.5
1.0
0.5
ppm

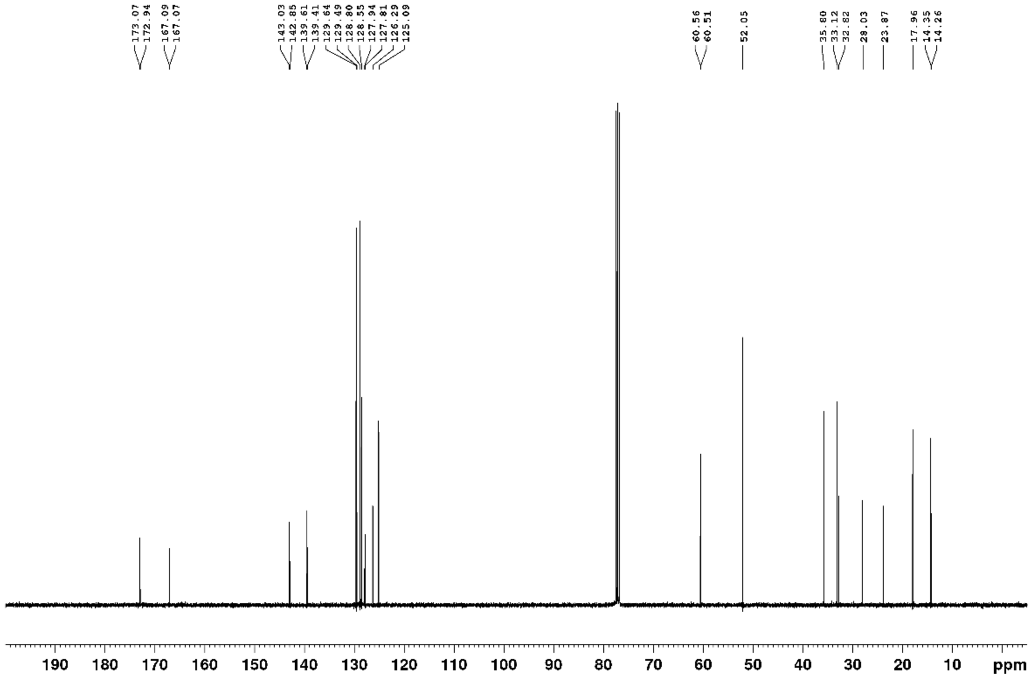
173.07
172.94
167.09
167.07
143.03
142.85
139.61
139.41
129.64
129.49
128.80
128.55
127.94
127.81
126.29
125.09
60.56
60.51
52.05
35.80
33.12
32.82
28.03
23.87
17.96
14.35
14.26
190
180
170
160
150
140
130
120
110
100
90
80
70
60
50
40
30
20
10
ppm

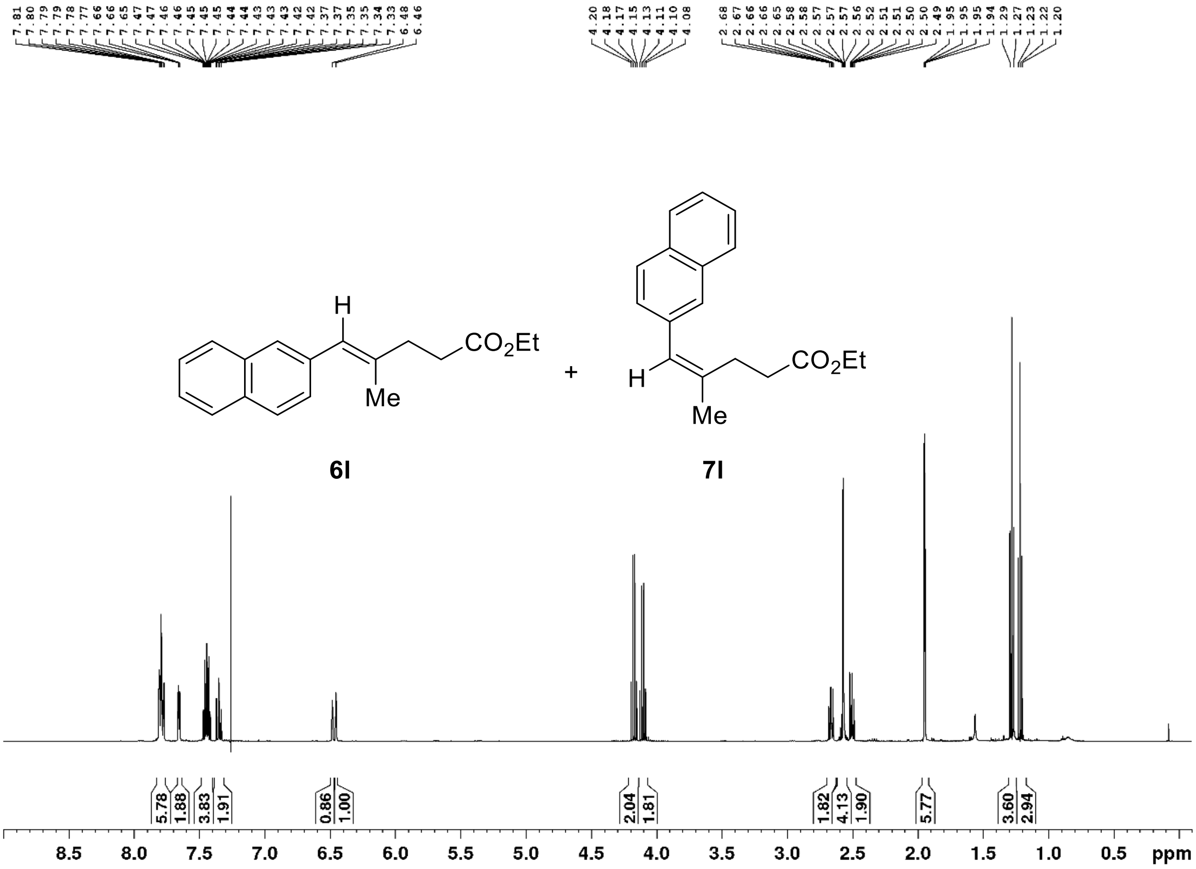
CO2Et
Me
H
6l
7l
ppm

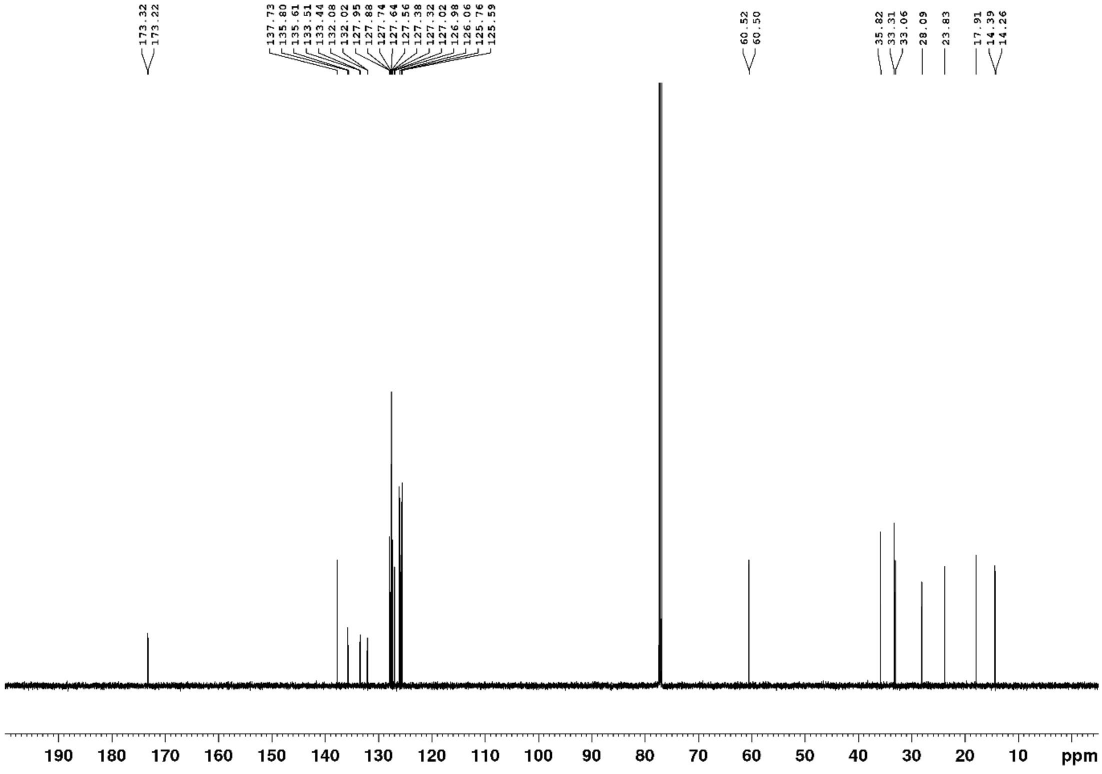
ppm

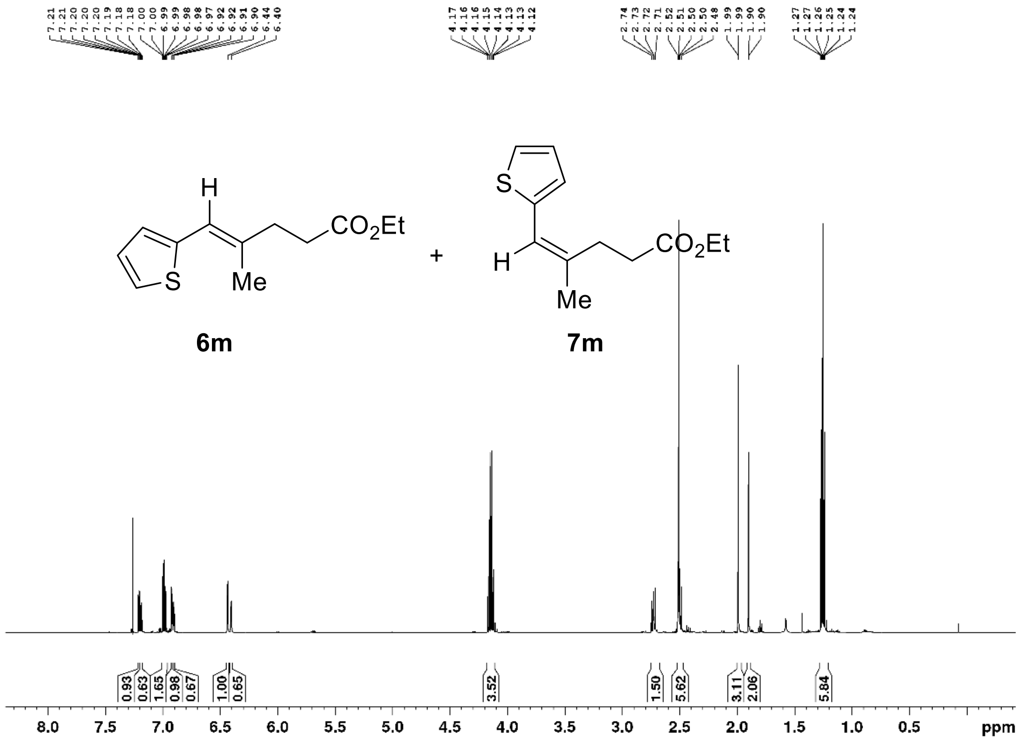
6m
7m
CO2Et
Me
ppm

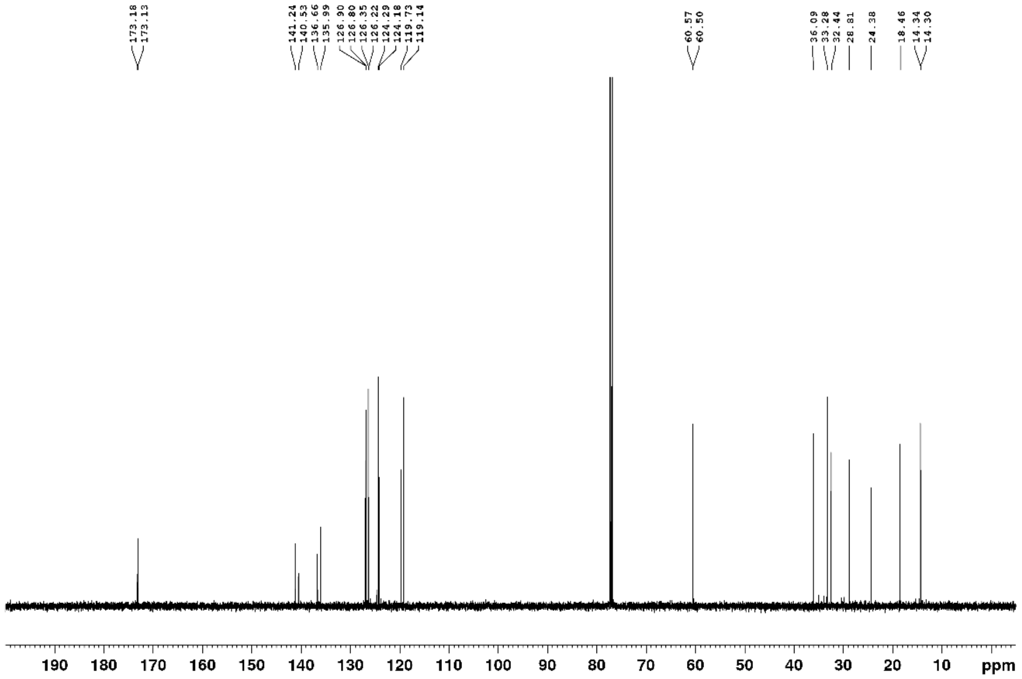
ppm

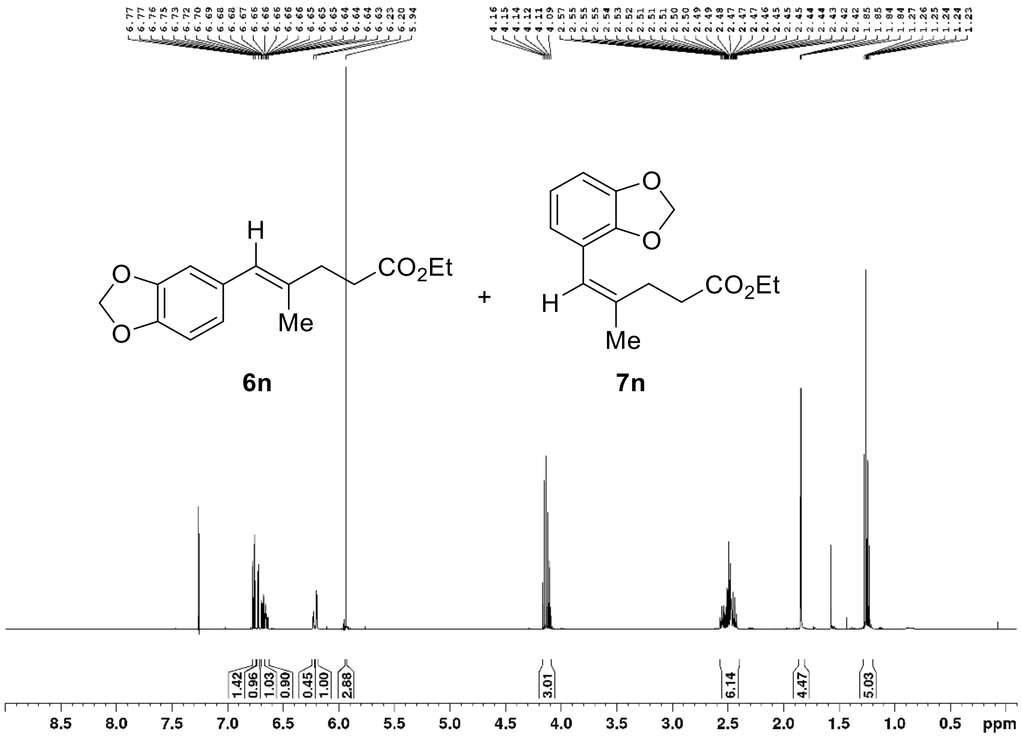
6.77 6.77 6.76 6.75 6.73 6.72 6.70 6.69 6.68 6.68 6.67 6.66 6.66 6.66 6.66 6.66 6.65 6.65 6.65 6.64 6.64 6.64 6.63 6.23 6.20 5.94
4.16 4.15 4.14 4.12 4.11 4.09 2.57 2.55 2.55 2.55 2.54 2.53 2.52 2.51 2.51 2.51 2.50 2.50 2.49 2.49 2.48 2.47 2.47 2.47 2.46 2.45 2.45 2.45 2.44 2.44 2.43 2.42 2.42 1.85 1.85 1.84 1.84 1.27 1.26 1.25 1.24 1.24 1.23
H
CO2Et
Me
+
H
CO2Et
Me
6n
7n
1.42 0.96 1.03 0.90 0.45 1.00 2.88 3.01 6.14 4.47 5.03
8.5 8.0 7.5 7.0 6.5 6.0 5.5 5.0 4.5 4.0 3.5 3.0 2.5 2.0 1.5 1.0 0.5 ppm

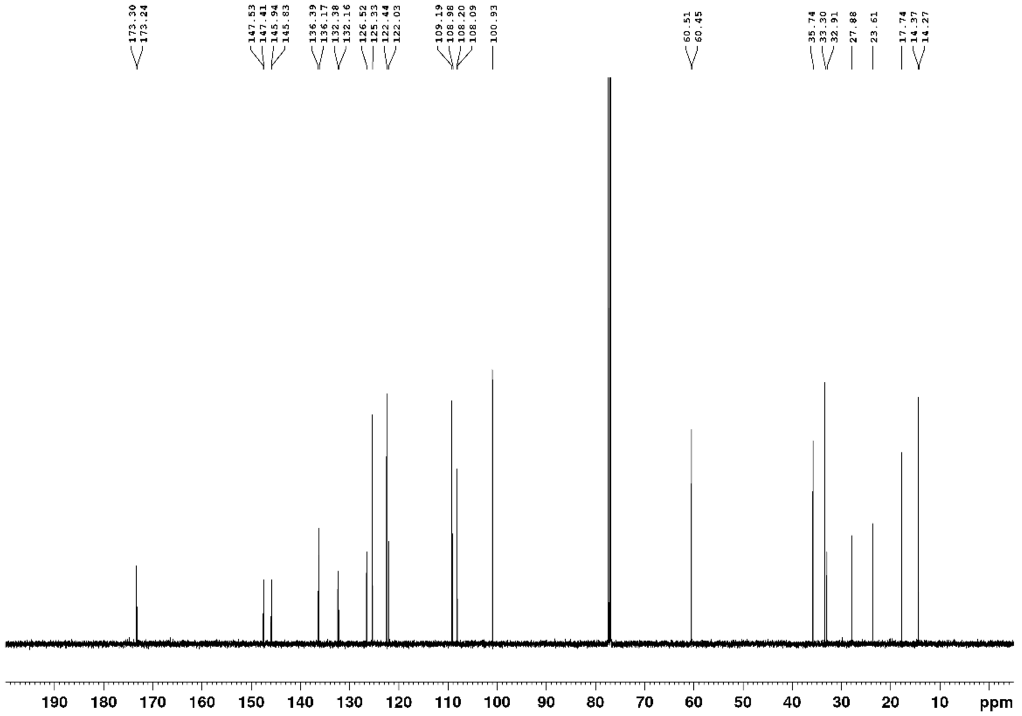
173.30 173.24
147.53 147.41 145.94 145.83
136.39 136.17 132.38 132.16 126.52 125.33 122.44 122.03
109.19 108.98 108.20 108.09 100.93
60.51 60.45
35.74 33.30 32.91 27.88 23.61 17.74 14.37 14.27
190 180 170 160 150 140 130 120 110 100 90 80 70 60 50 40 30 20 10 ppm

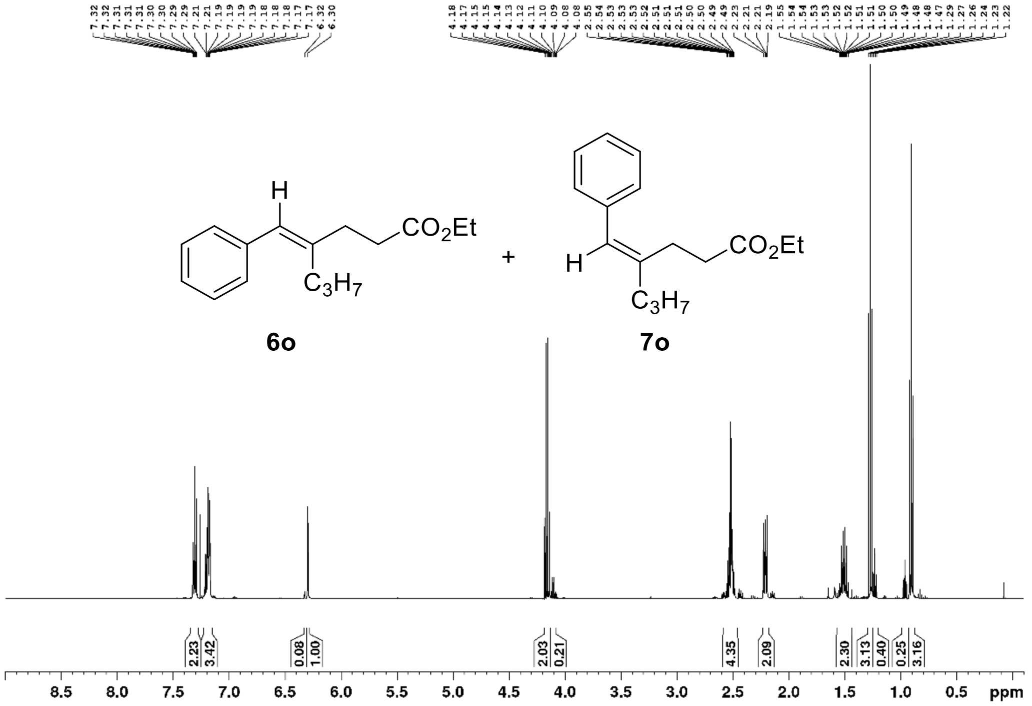
H
CO2Et
C3H7
+
H
CO2Et
C3H7
6o
7o
8.5 8.0 7.5 7.0 6.5 6.0 5.5 5.0 4.5 4.0 3.5 3.0 2.5 2.0 1.5 1.0 0.5 ppm

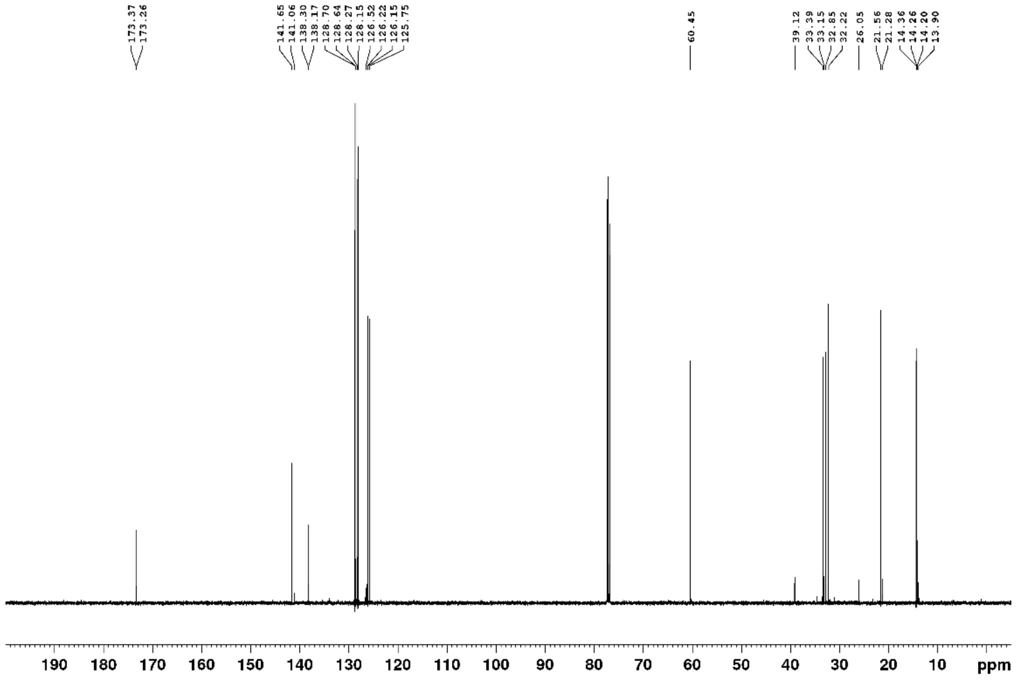
190 180 170 160 150 140 130 120 110 100 90 80 70 60 50 40 30 20 10 ppm

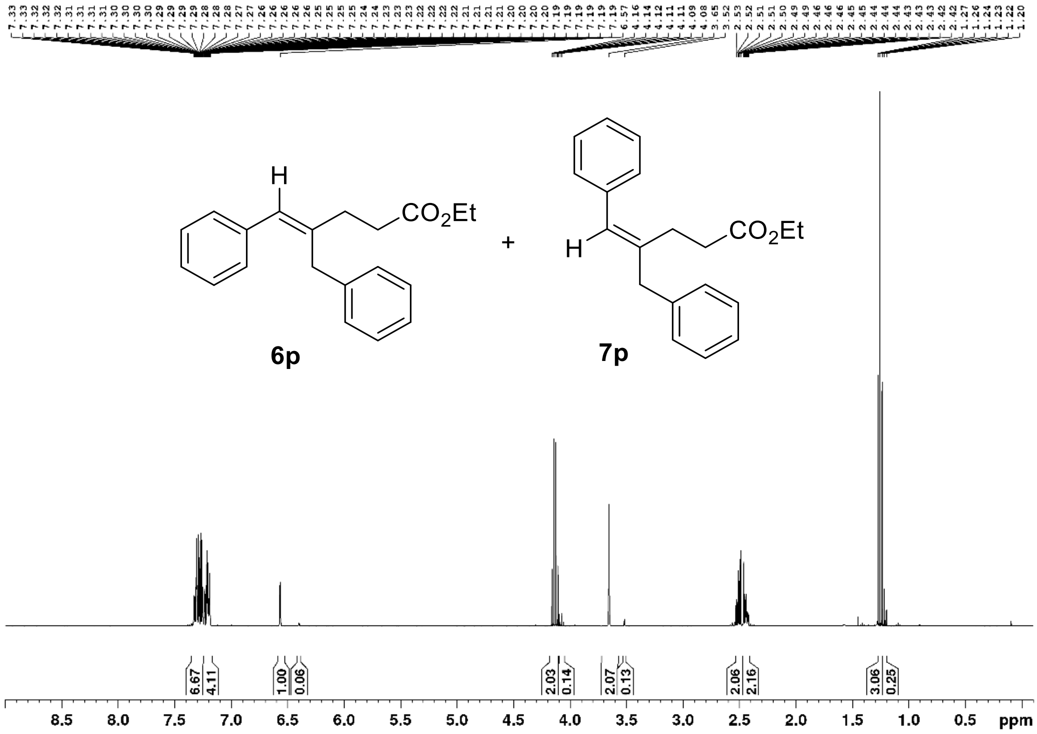
H
CO2Et
+
H
CO2Et
6p
7p
6.67
4.11
1.00
0.06
2.03
0.14
2.07
0.13
2.06
2.16
3.06
0.25
8.5
8.0
7.5
7.0
6.5
6.0
5.5
5.0
4.5
4.0
3.5
3.0
2.5
2.0
1.5
1.0
0.5
ppm

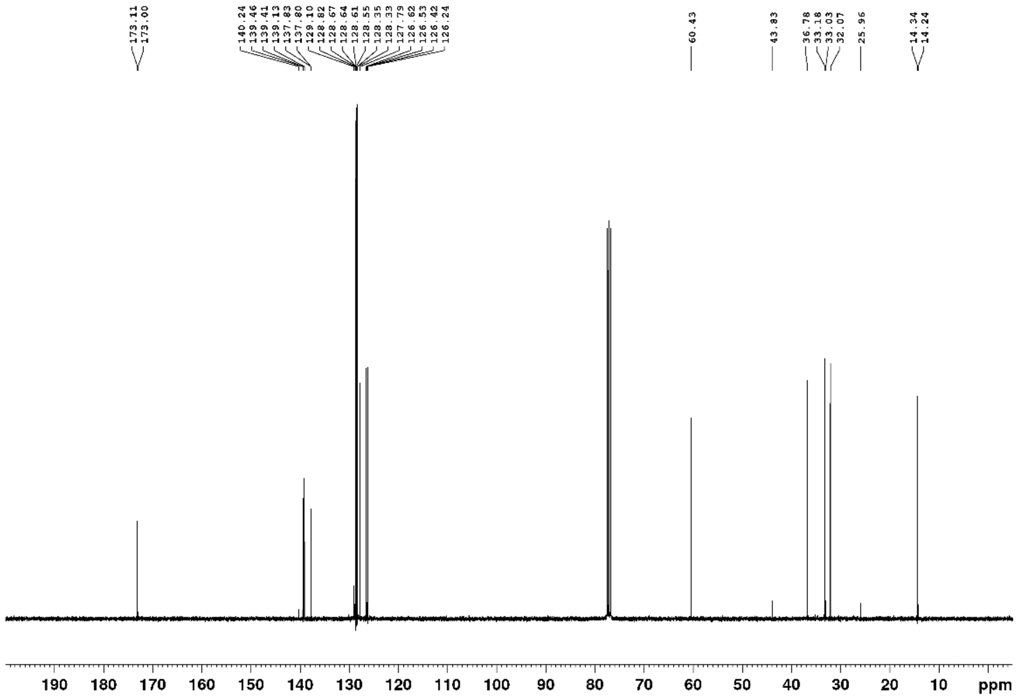
173.11
173.00
140.24
139.46
139.41
139.13
137.83
137.80
129.10
128.82
128.67
128.64
128.61
128.55
128.35
128.33
127.79
126.62
126.53
126.42
126.24
60.43
43.83
36.78
33.18
33.03
32.07
25.96
14.34
14.24
190
180
170
160
150
140
130
120
110
100
90
80
70
60
50
40
30
20
10
ppm

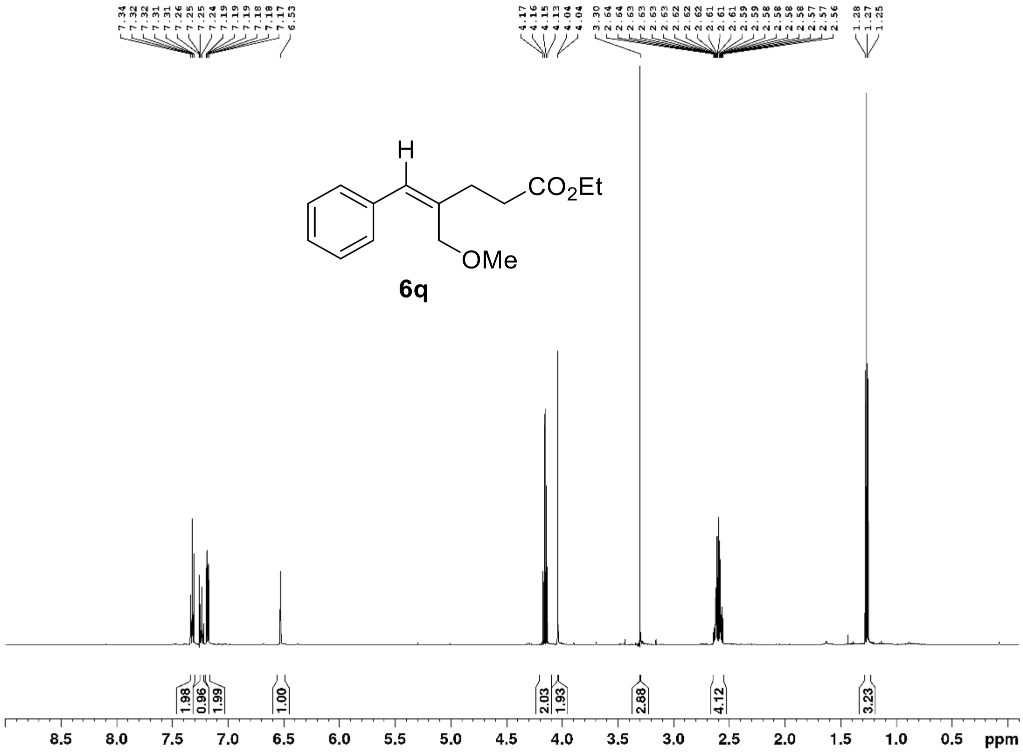
H
CO2Et
OMe
6q
1.98
0.96
1.99
1.00
2.03
1.93
2.88
4.12
3.23
8.5 8.0 7.5 7.0 6.5 6.0 5.5 5.0 4.5 4.0 3.5 3.0 2.5 2.0 1.5 1.0 0.5 ppm

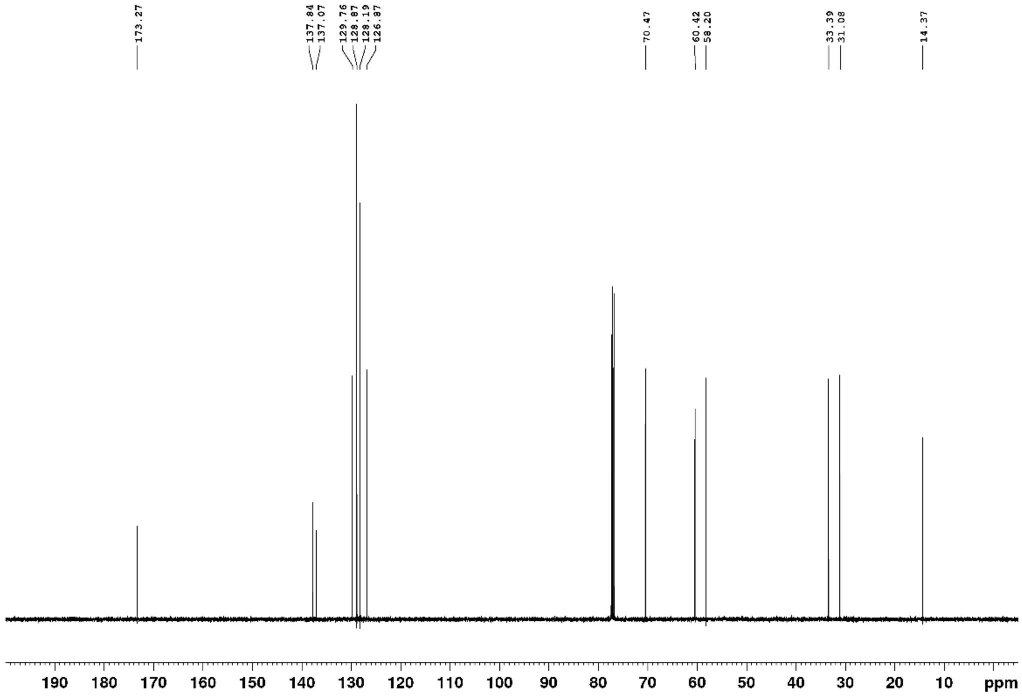
173.27
137.84
137.07
129.76
128.87
128.19
126.87
70.47
60.42
58.20
33.39
31.08
14.37
190 180 170 160 150 140 130 120 110 100 90 80 70 60 50 40 30 20 10 ppm

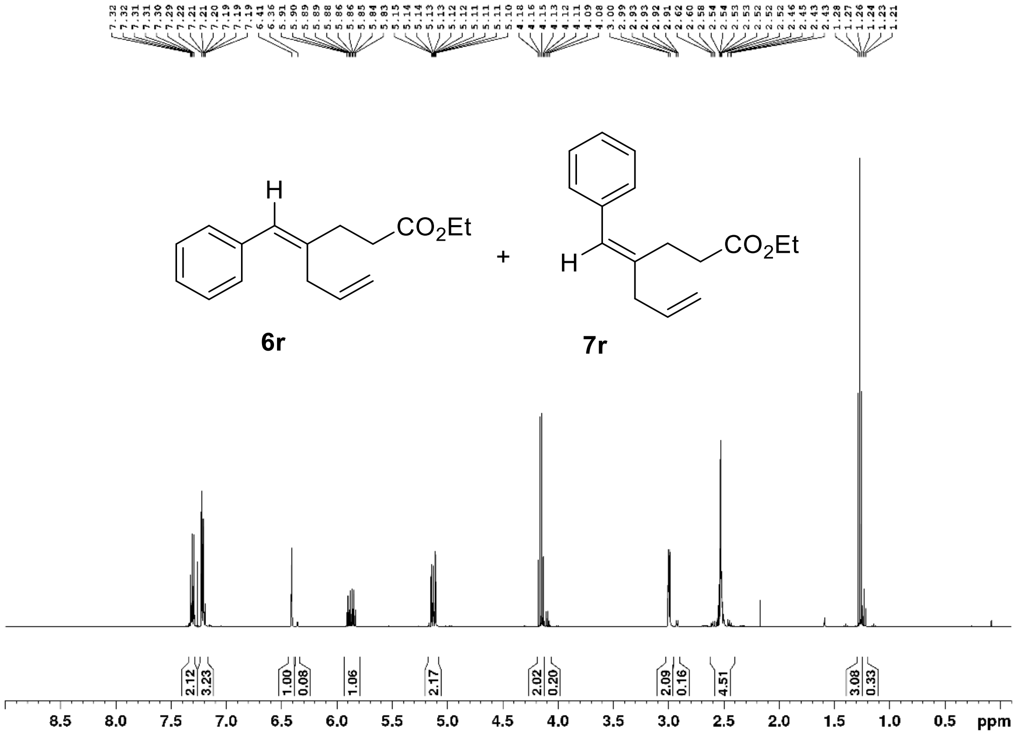
H
CO2Et
+
6r
7r
ppm

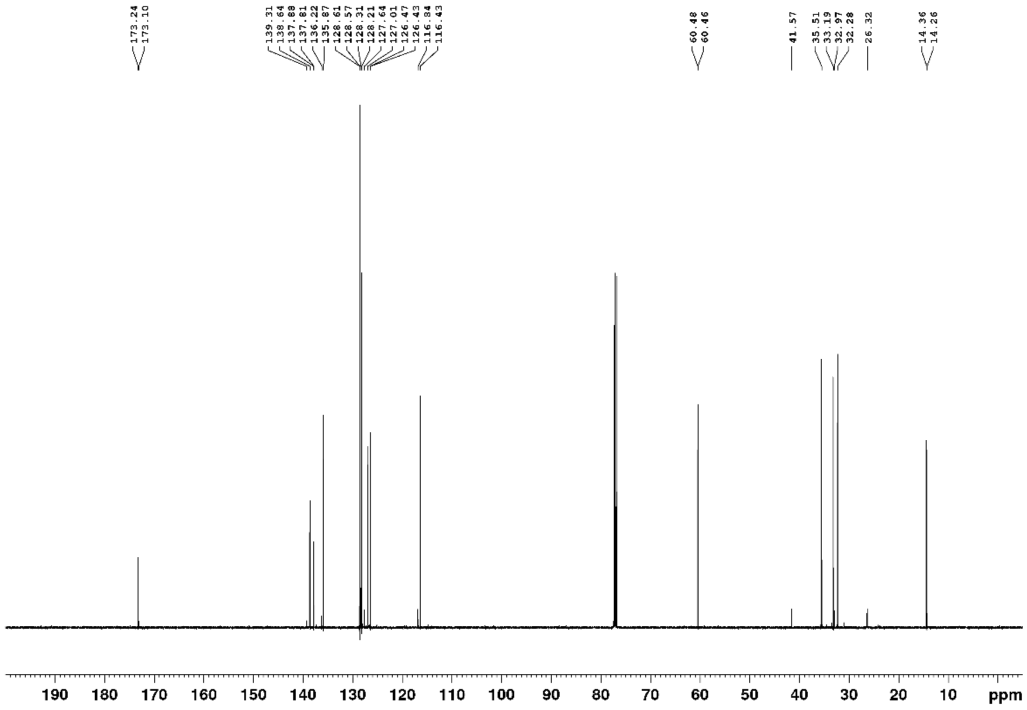
ppm

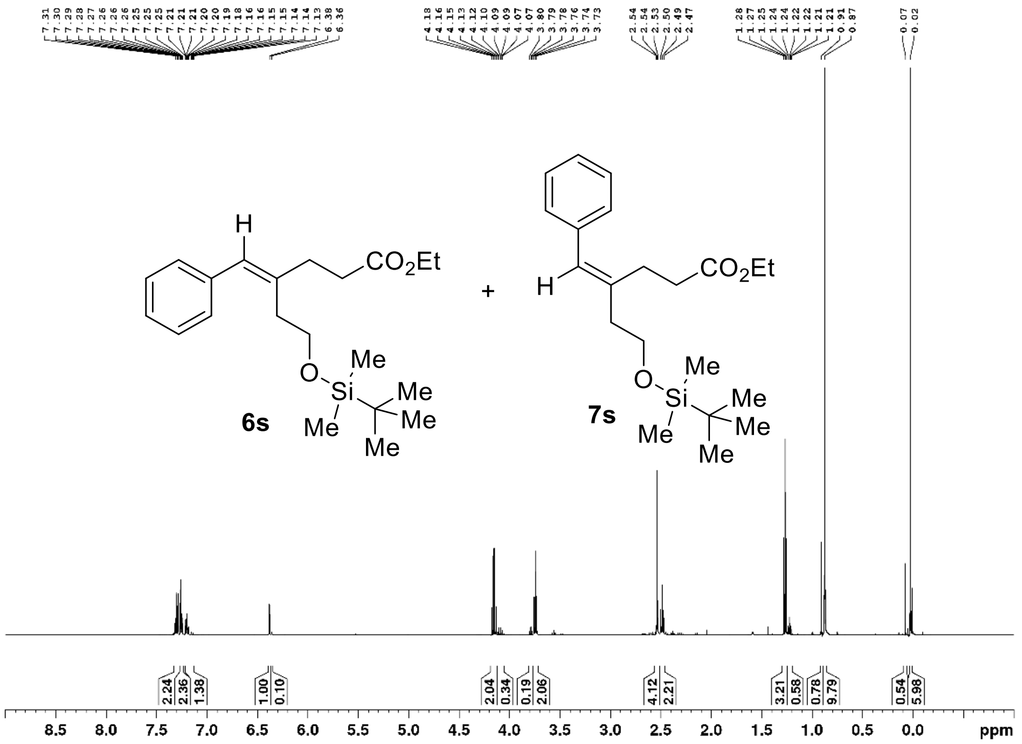

CO2Et
+
H
6s
7s
Me
Si
O
ppm

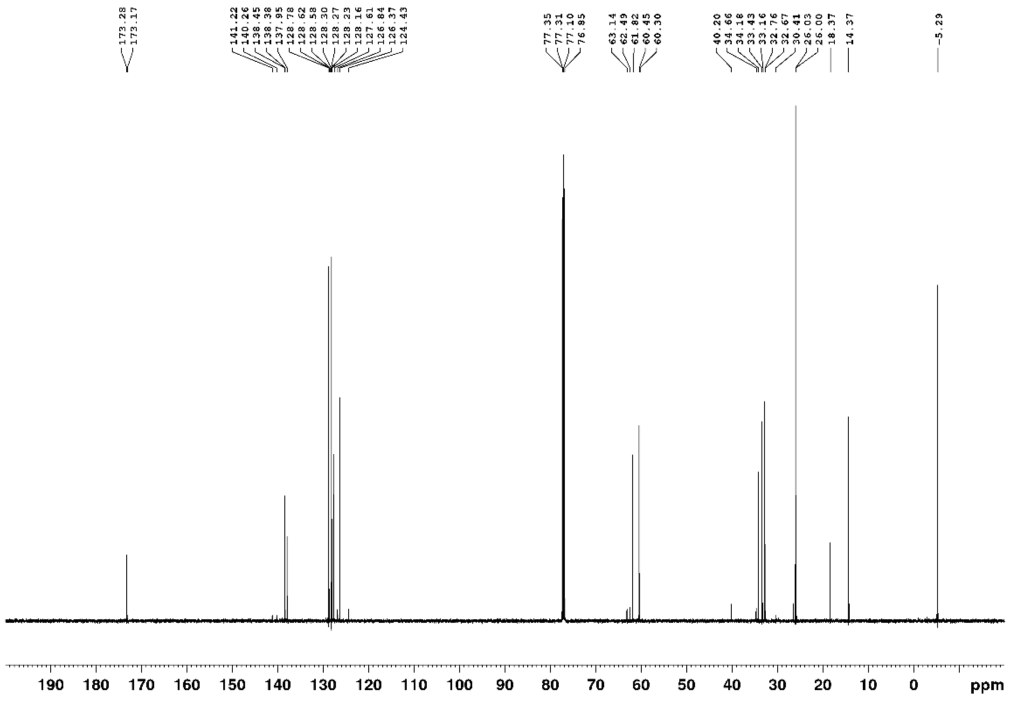

ppm

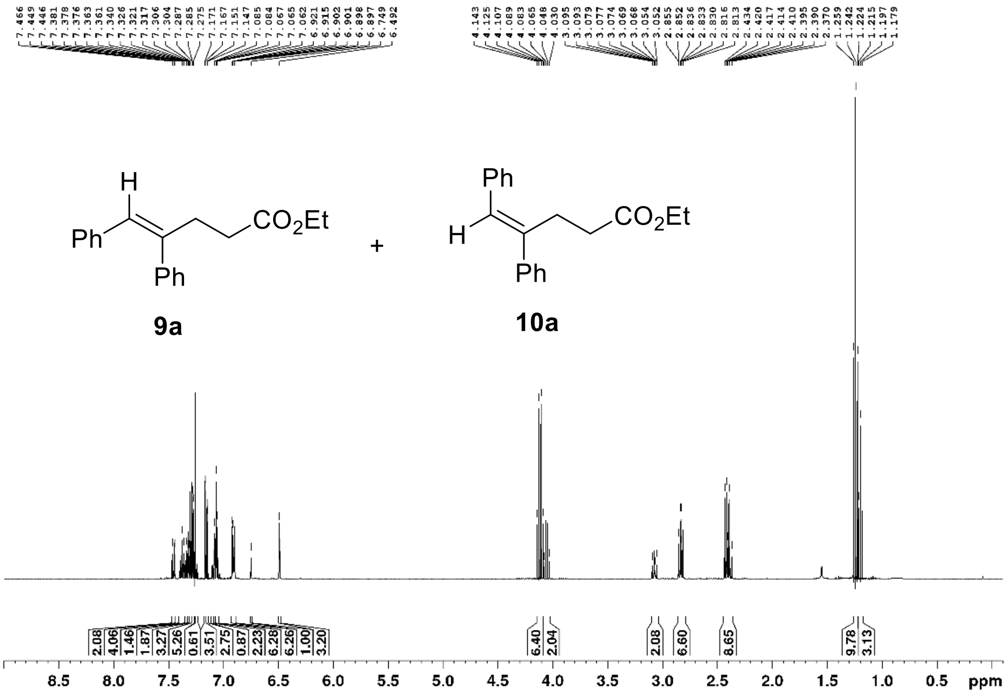
Ph
CO2Et
Ph
H
+
Ph
H
CO2Et
Ph
9a
10a
ppm

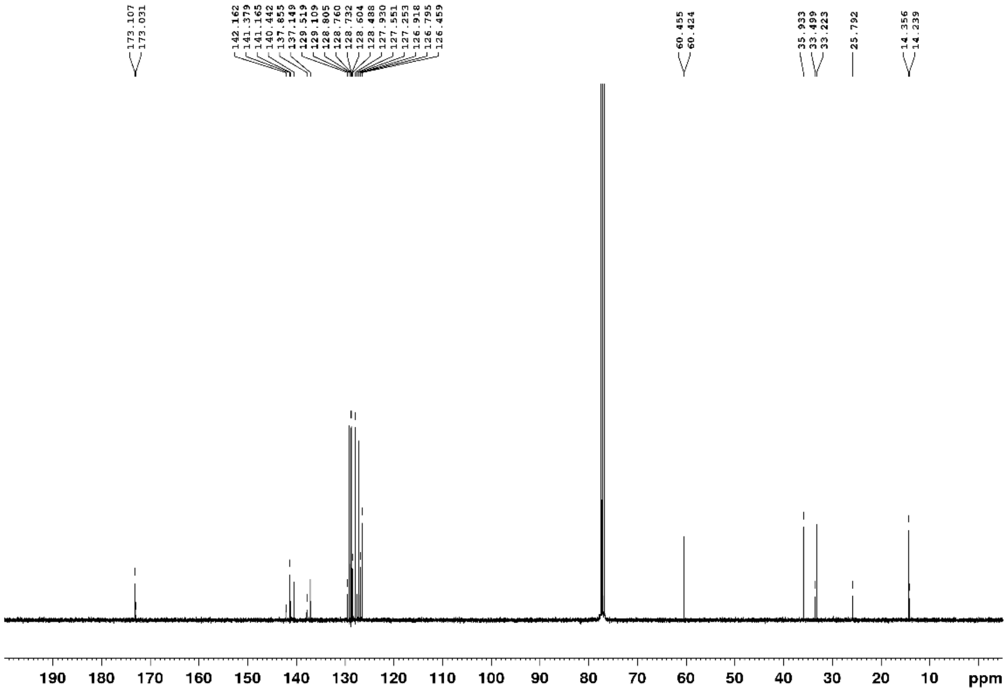
ppm

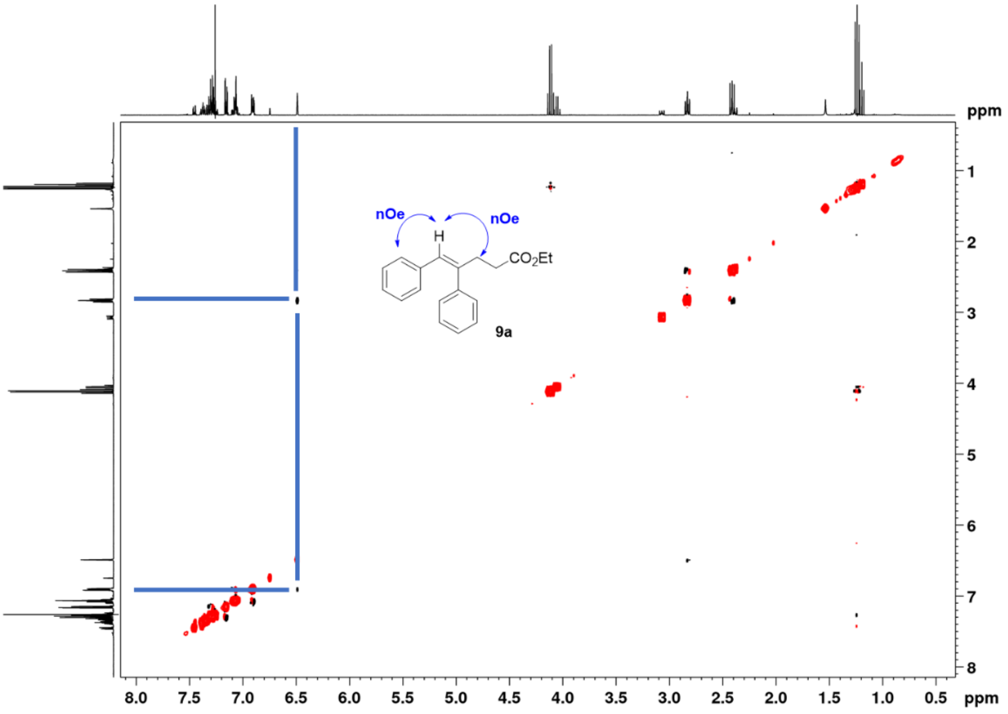
nOe
nOe
H
CO_2Et
9a
ppm
1
2
3
4
5
6
7
8
8.0
7.5
7.0
6.5
6.0
5.5
5.0
4.5
4.0
3.5
3.0
2.5
2.0
1.5
1.0
0.5
ppm

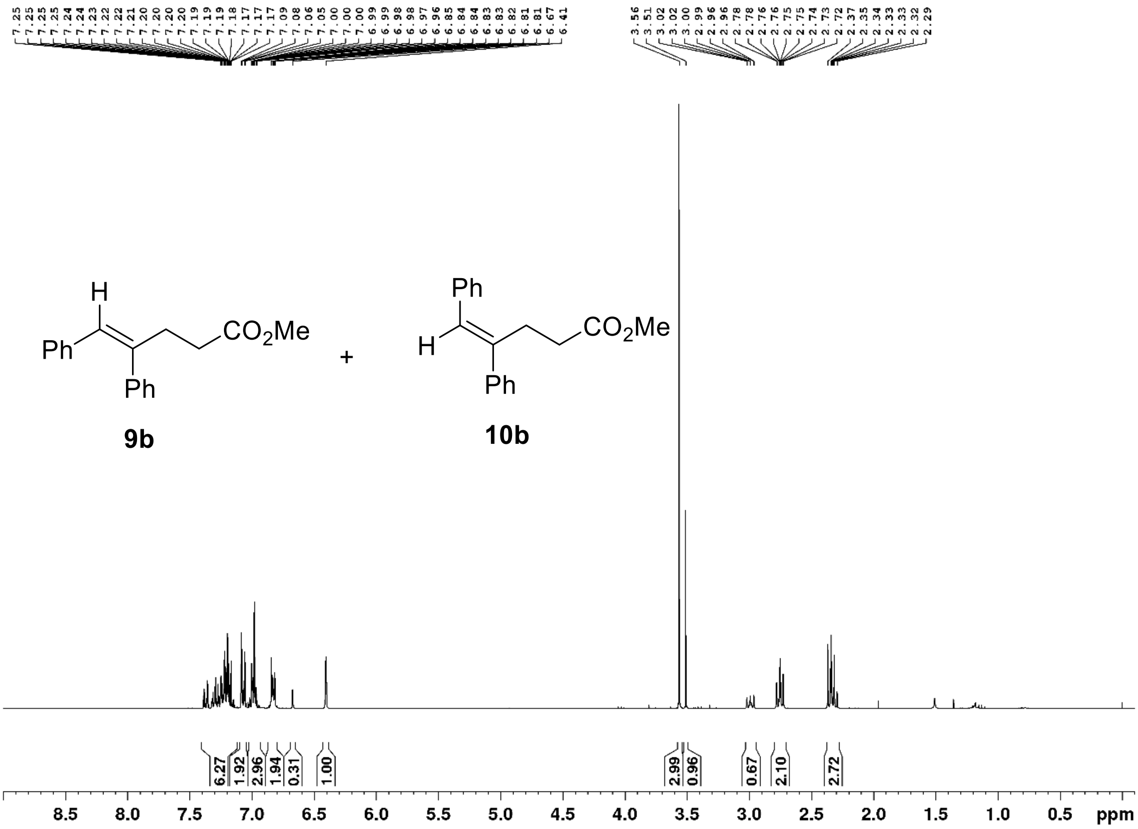
H
Ph
CO2Me
Ph
9b
+
Ph
H
CO2Me
Ph
10b
6.27 1.92 2.96 1.94 0.31 1.00 2.99 0.96 0.67 2.10 2.72
8.5 8.0 7.5 7.0 6.5 6.0 5.5 5.0 4.5 4.0 3.5 3.0 2.5 2.0 1.5 1.0 0.5 ppm

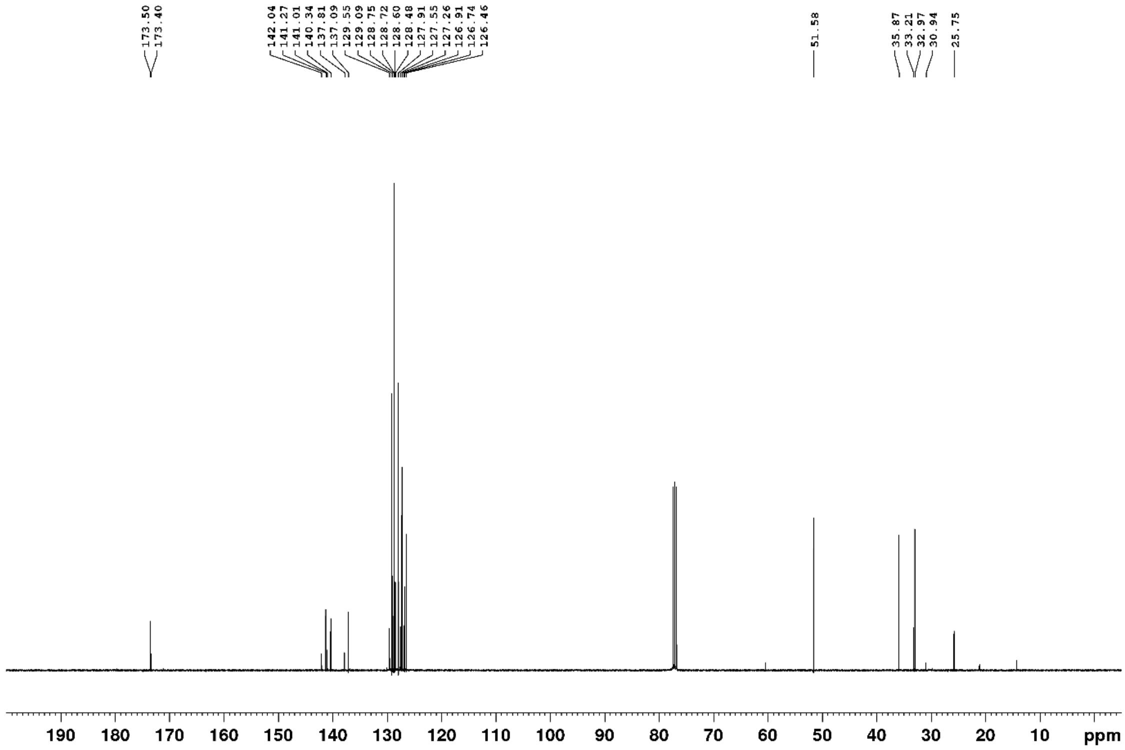
173.50
173.40
142.04
141.27
141.01
140.34
137.81
137.09
129.55
129.09
128.75
128.72
128.60
128.48
127.91
127.55
127.26
126.91
126.74
126.46
51.58
35.87
33.21
32.97
30.94
25.75
190 180 170 160 150 140 130 120 110 100 90 80 70 60 50 40 30 20 10 ppm

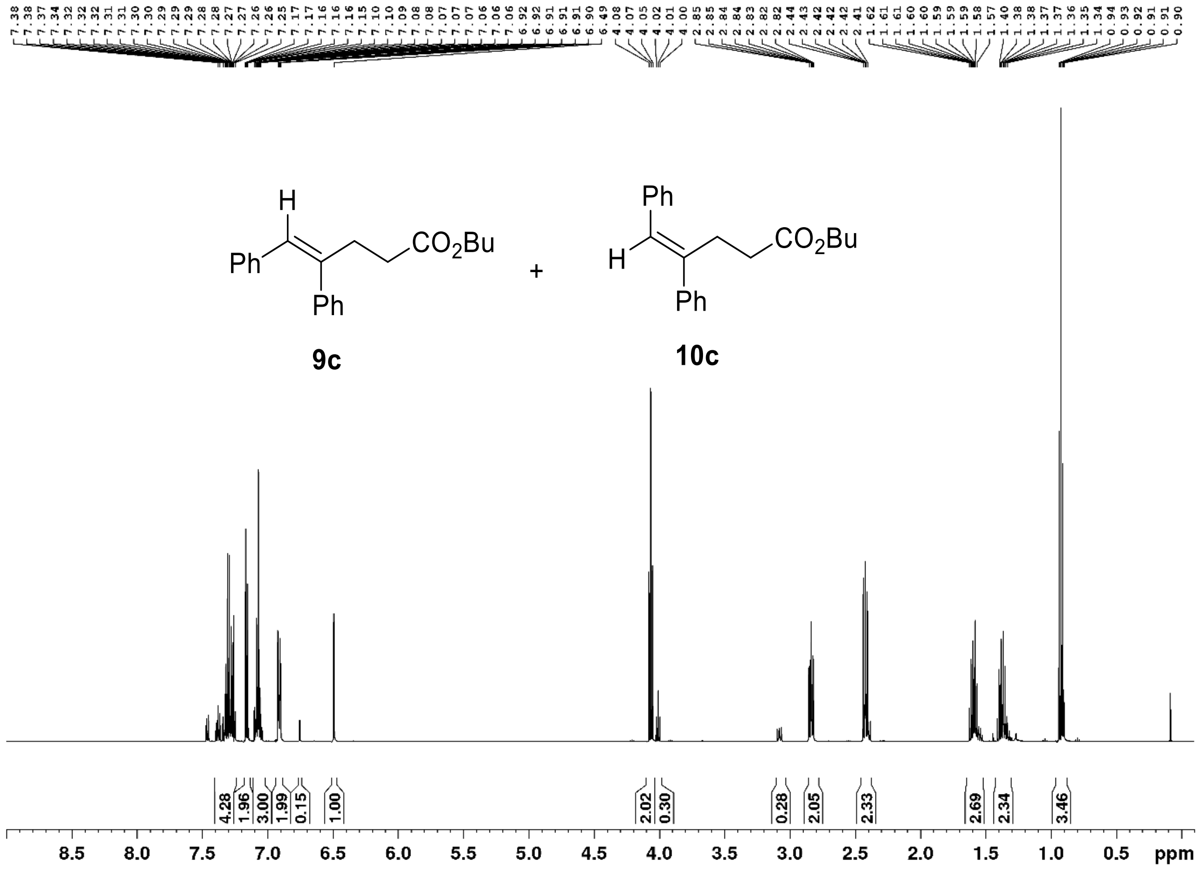
Ph
CO2Bu
H
Ph
9c
+
10c
ppm

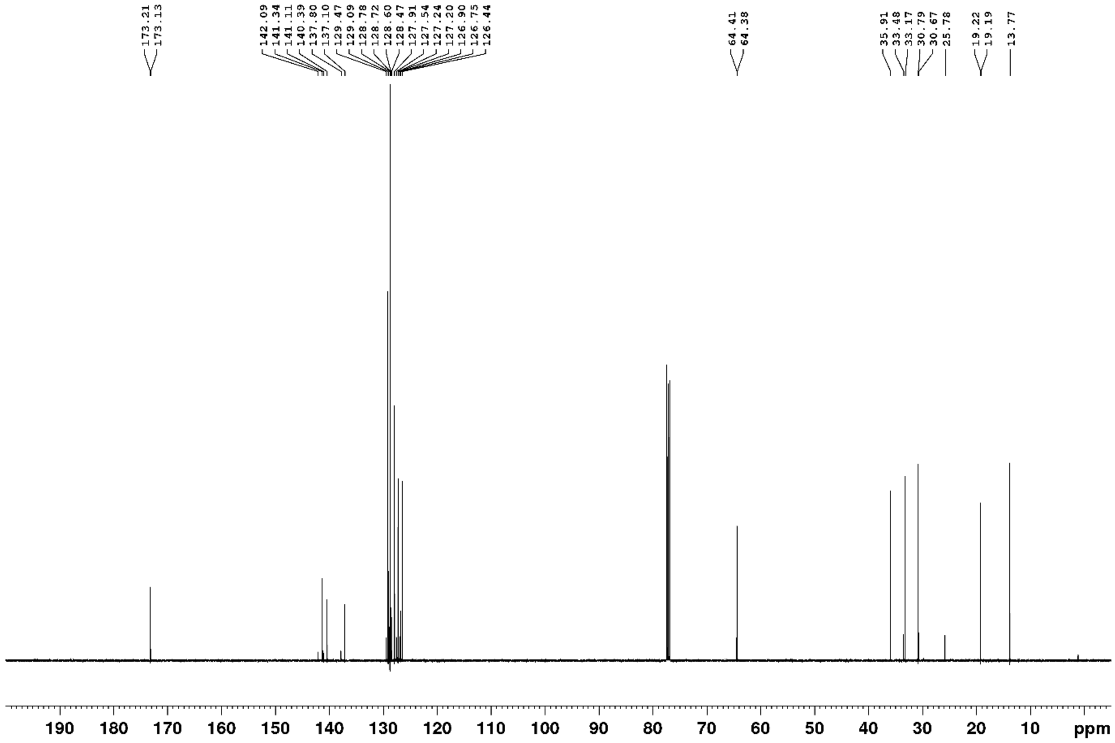
173.21
173.13
64.41
64.38
35.91
33.48
33.17
30.79
30.67
25.78
19.22
19.19
13.77
ppm

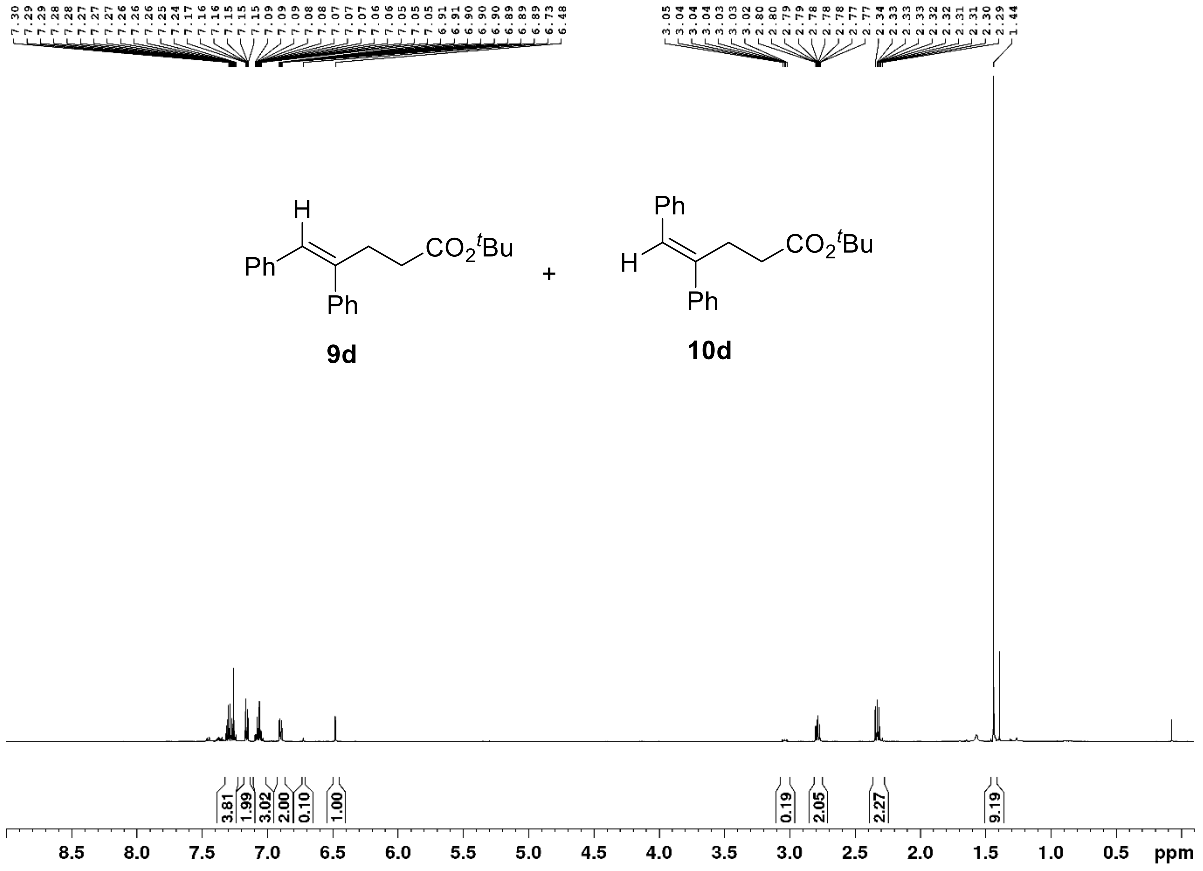
Ph
H
Ph
CO2tBu
+
Ph
H
Ph
CO2tBu
9d
10d
3.81
1.99
3.02
2.00
0.10
1.00
0.19
2.05
2.27
9.19
ppm

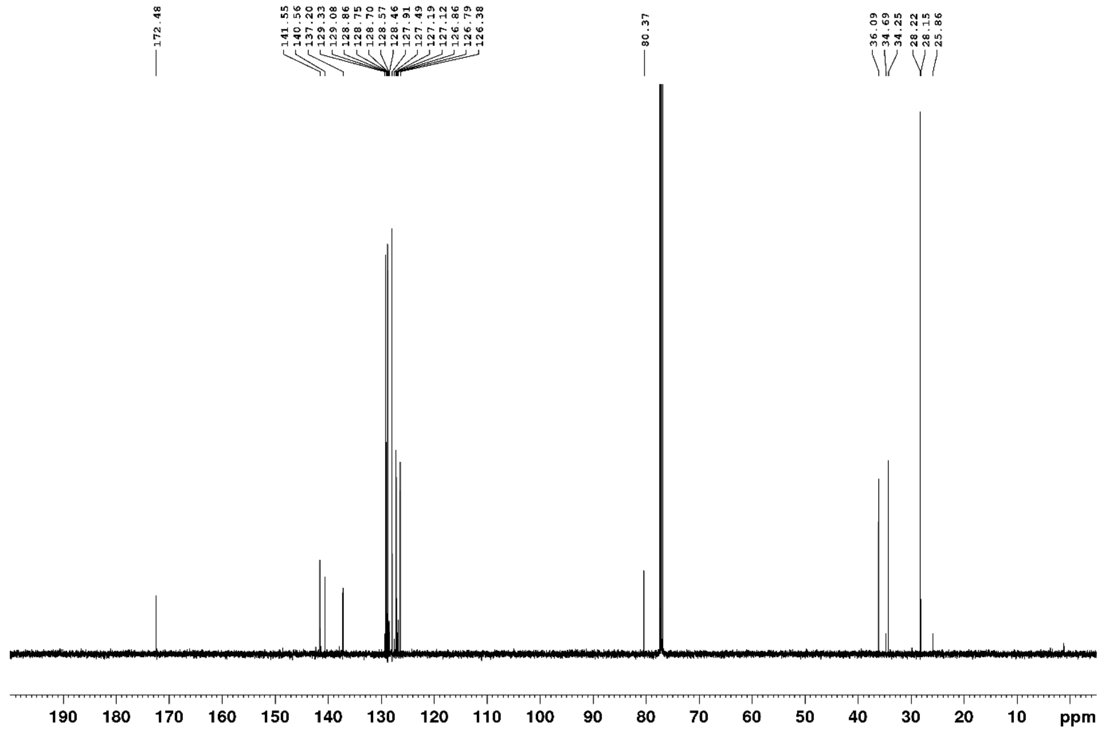
172.48
80.37
ppm

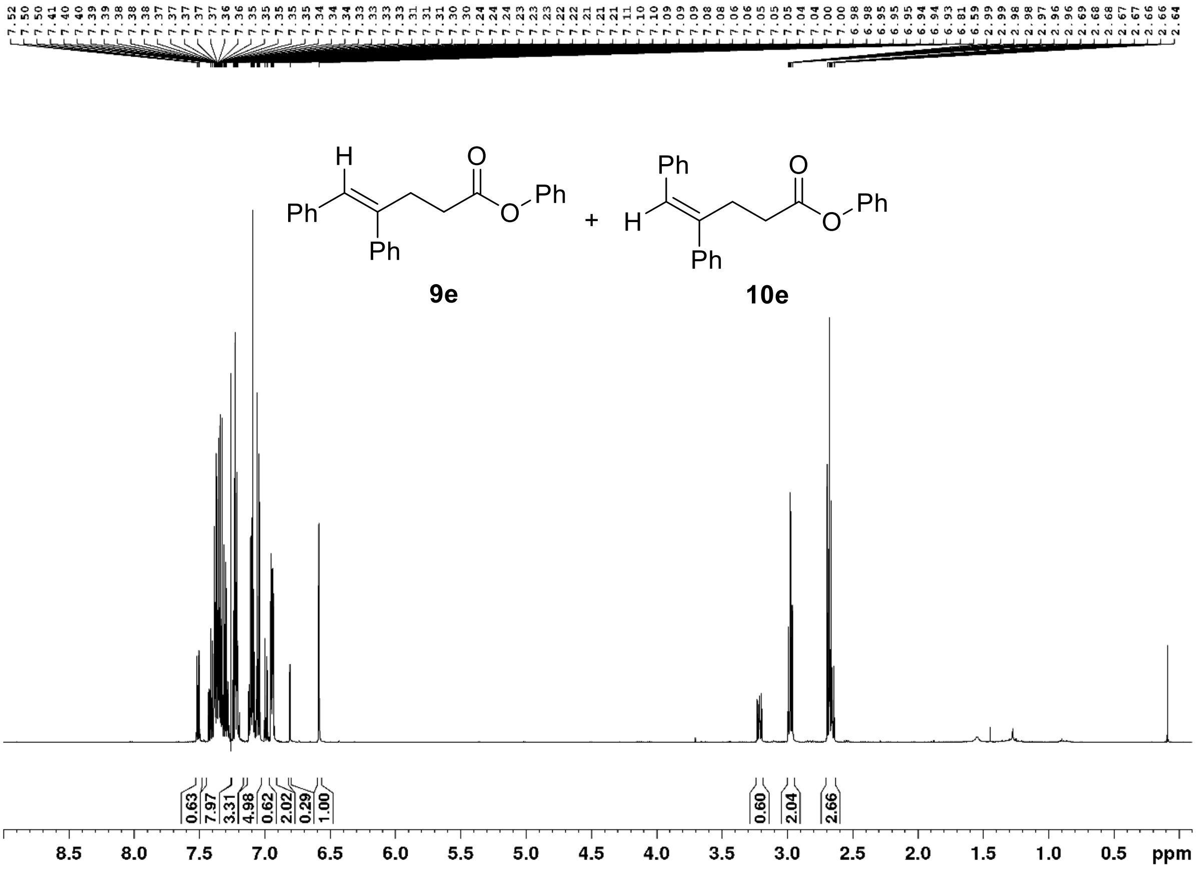
9e
10e
Ph
O
H
+
0.63
7.97
3.31
4.98
0.62
2.02
0.29
1.00
0.60
2.04
2.66
8.5 8.0 7.5 7.0 6.5 6.0 5.5 5.0 4.5 4.0 3.5 3.0 2.5 2.0 1.5 1.0 0.5 ppm

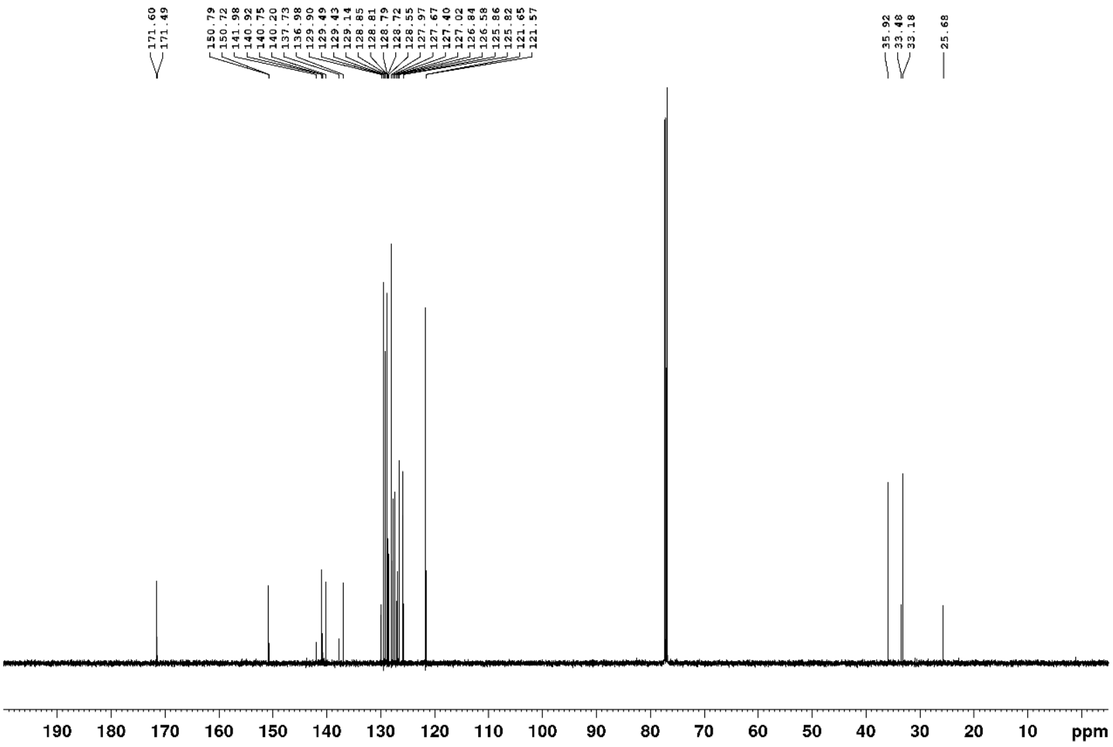
171.60
171.49
150.79
150.72
141.98
140.92
140.75
140.20
137.73
136.98
129.90
129.49
129.43
129.14
128.85
128.81
128.79
128.72
128.55
127.97
127.67
127.40
127.02
126.84
126.58
125.86
125.82
121.65
121.57
35.92
33.48
33.18
25.68
190 180 170 160 150 140 130 120 110 100 90 80 70 60 50 40 30 20 10 ppm

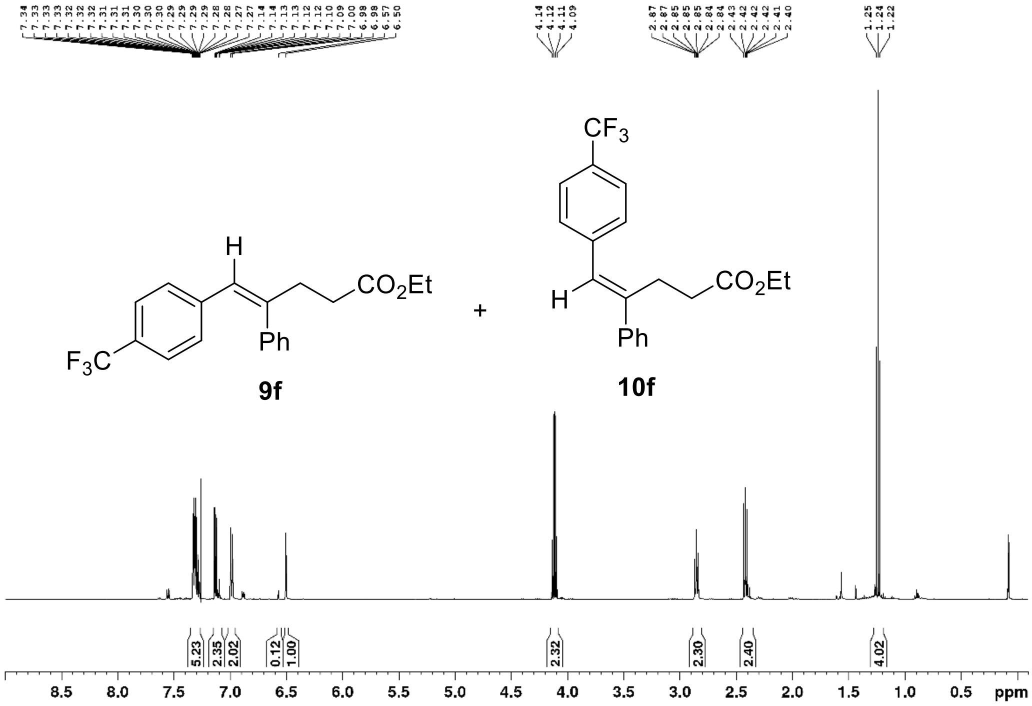
CF3
H
CO2Et
+
H
CO2Et
F3C
Ph
Ph
9f
10f
ppm

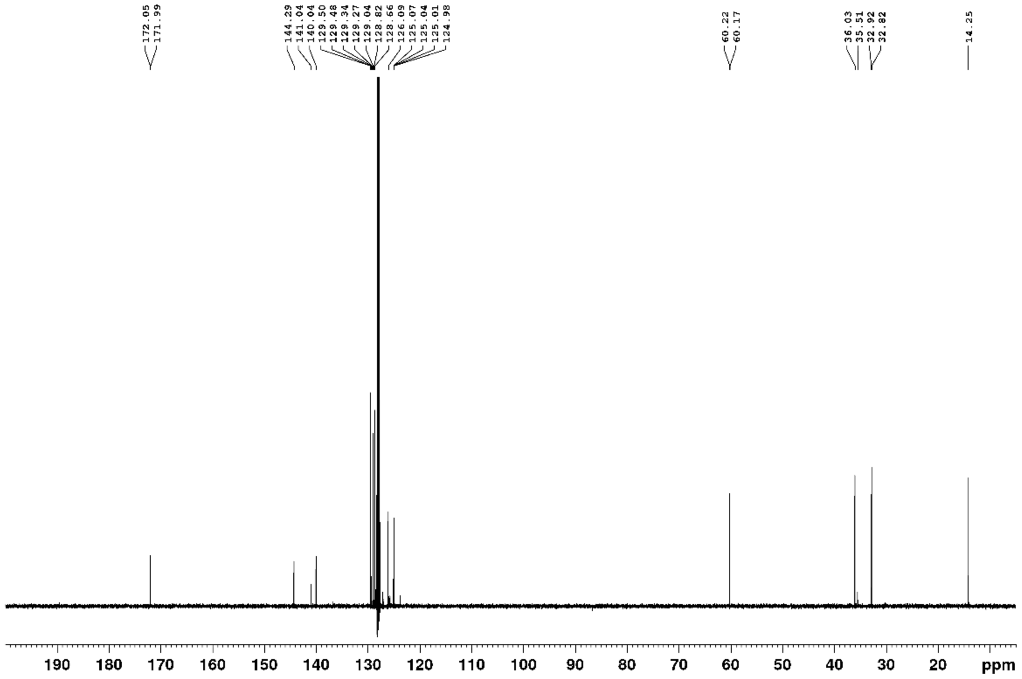
ppm

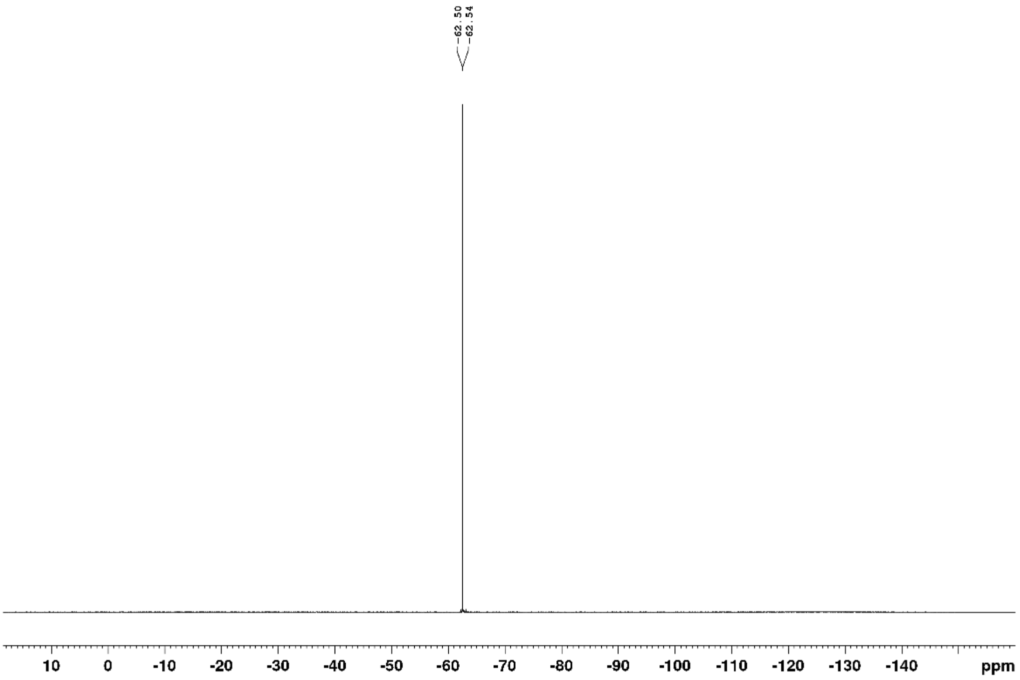
-62.50
-62.54
10
0
-10
-20
-30
-40
-50
-60
-70
-80
-90
-100
-110
-120
-130
-140
ppm

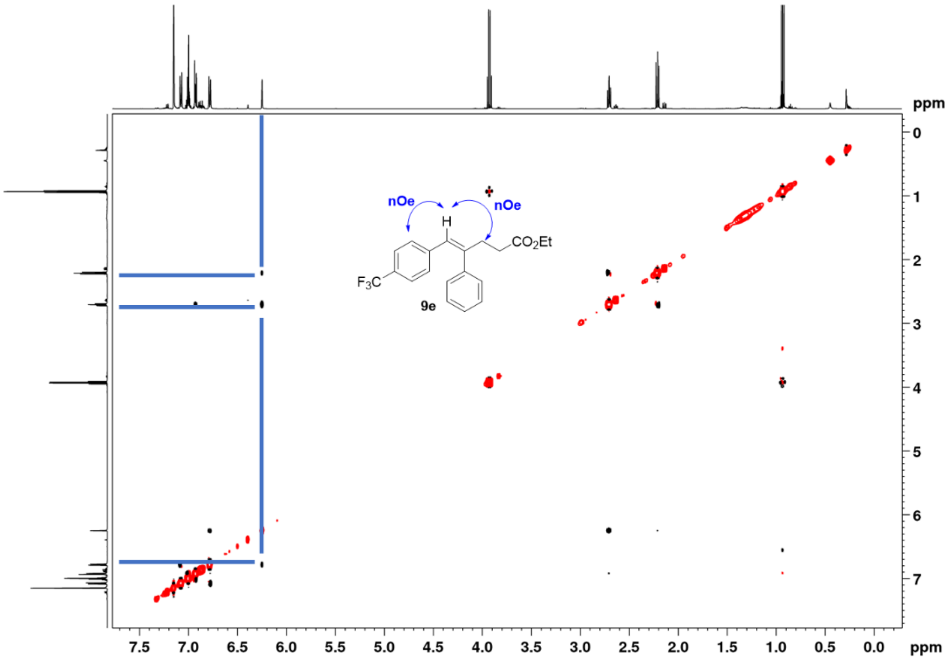
nOe
nOe
H
CO_2Et
F_3C
9e
ppm

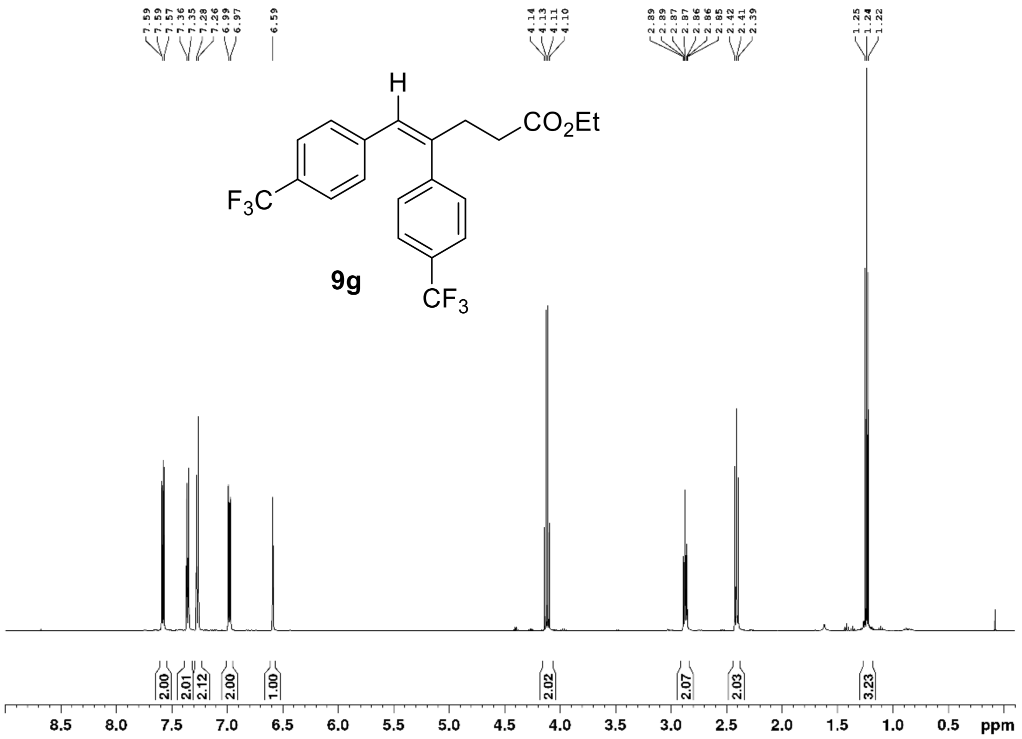
7.59
7.59
7.57
7.36
7.35
7.28
7.26
6.99
6.97
6.59
4.14
4.13
4.11
4.10
2.89
2.89
2.87
2.87
2.86
2.86
2.85
2.42
2.41
2.39
1.25
1.24
1.22
H
CO2Et
F3C
CF3
9g
2.00
2.01
2.12
2.00
1.00
2.02
2.07
2.03
3.23
8.5 8.0 7.5 7.0 6.5 6.0 5.5 5.0 4.5 4.0 3.5 3.0 2.5 2.0 1.5 1.0 0.5 ppm

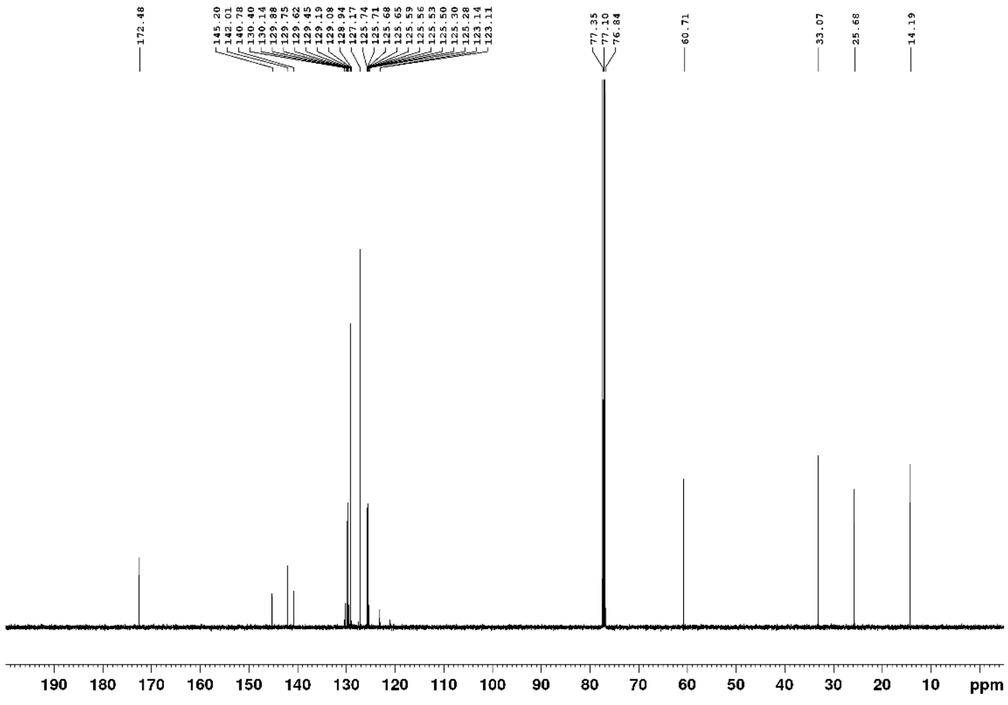
172.48
145.20
142.01
140.78
130.40
130.14
129.88
129.75
129.62
129.45
129.19
129.08
128.94
127.17
125.74
125.71
125.68
125.65
125.59
125.56
125.53
125.50
125.30
123.28
123.14
123.11
77.35
77.10
76.84
60.71
33.07
25.68
14.19
190 180 170 160 150 140 130 120 110 100 90 80 70 60 50 40 30 20 10 ppm

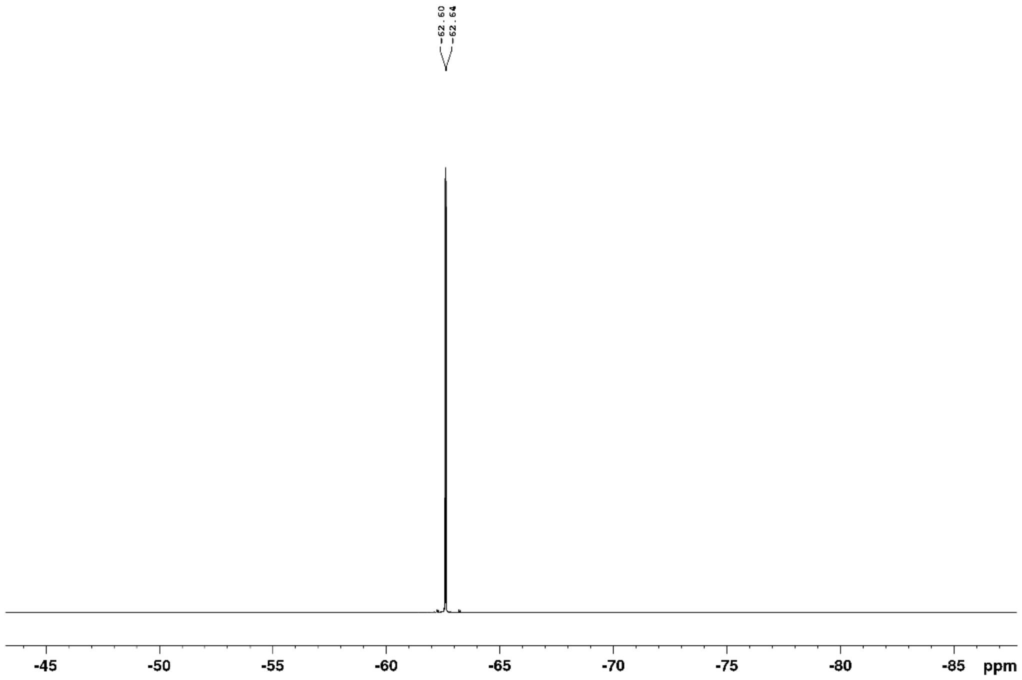
-62.60
-62.64
-45
-50
-55
-60
-65
-70
-75
-80
-85
ppm

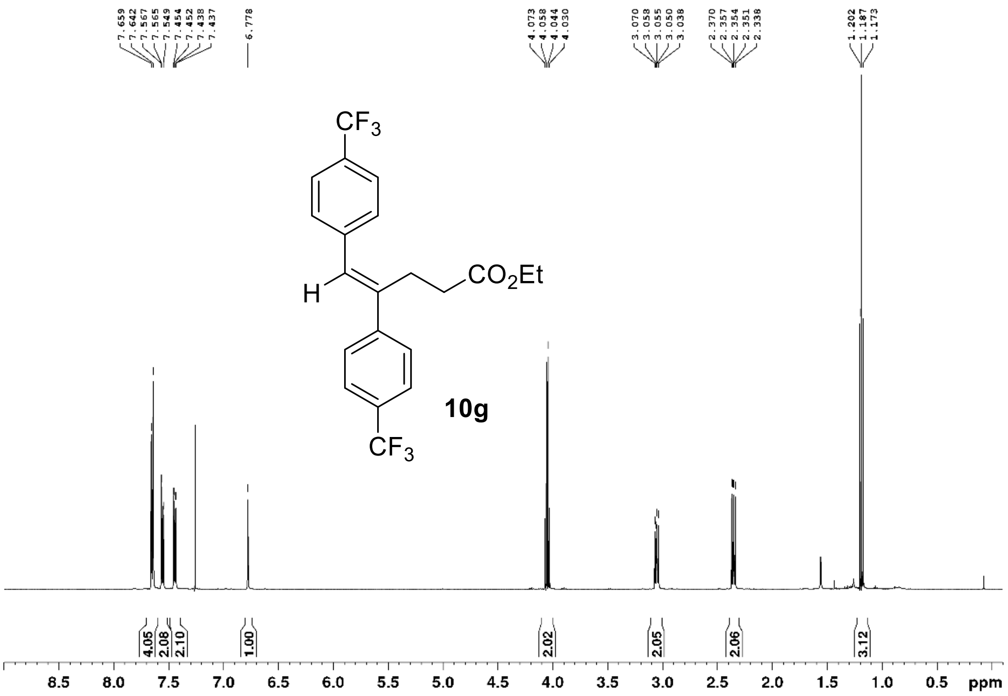
7.659
7.642
7.567
7.565
7.549
7.454
7.452
7.438
7.437
6.778
4.073
4.058
4.044
4.030
3.070
3.058
3.055
3.050
3.038
2.370
2.357
2.354
2.351
2.338
1.202
1.187
1.173
CF3
H
CO2Et
CF3
10g
4.05
2.08
2.10
1.00
2.02
2.05
2.06
3.12
8.5 8.0 7.5 7.0 6.5 6.0 5.5 5.0 4.5 4.0 3.5 3.0 2.5 2.0 1.5 1.0 0.5 ppm

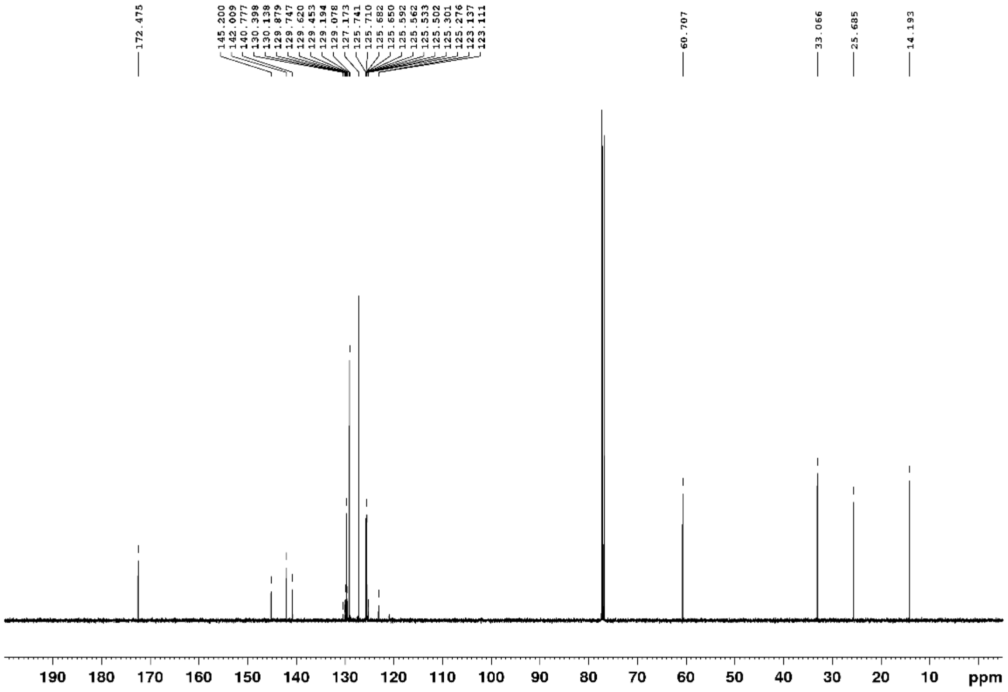
172.475
145.200
142.009
140.777
130.398
130.138
129.879
129.747
129.620
129.453
129.194
129.078
127.173
125.741
125.710
125.682
125.650
125.592
125.562
125.533
125.502
125.301
125.276
123.137
123.111
60.707
33.066
25.685
14.193
190 180 170 160 150 140 130 120 110 100 90 80 70 60 50 40 30 20 10 ppm

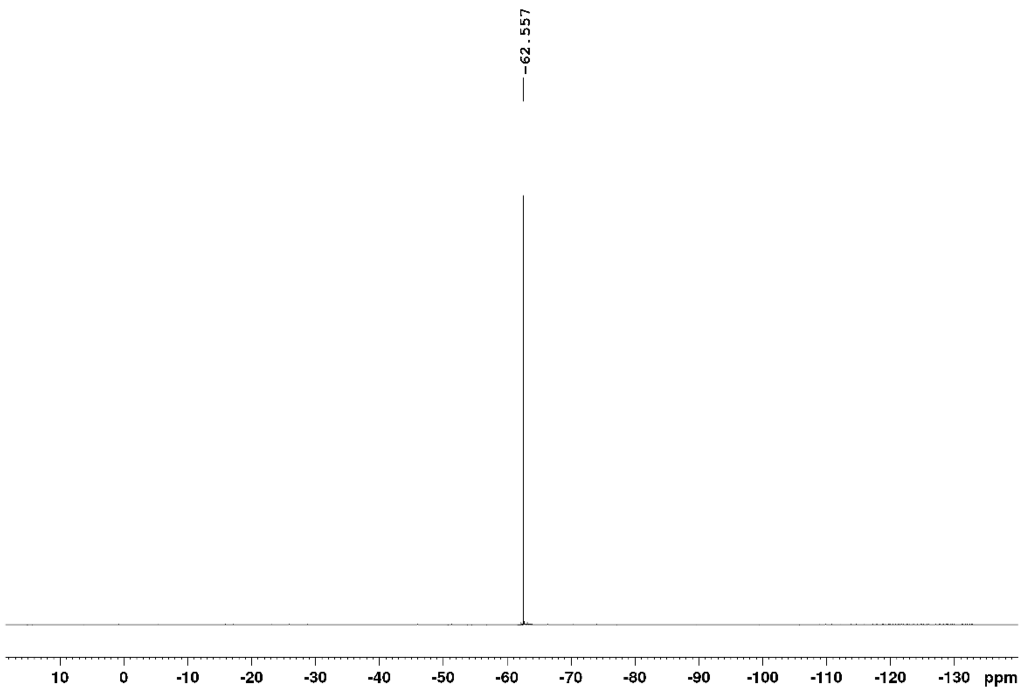
-62.557
10
0
-10
-20
-30
-40
-50
-60
-70
-80
-90
-100
-110
-120
-130
ppm

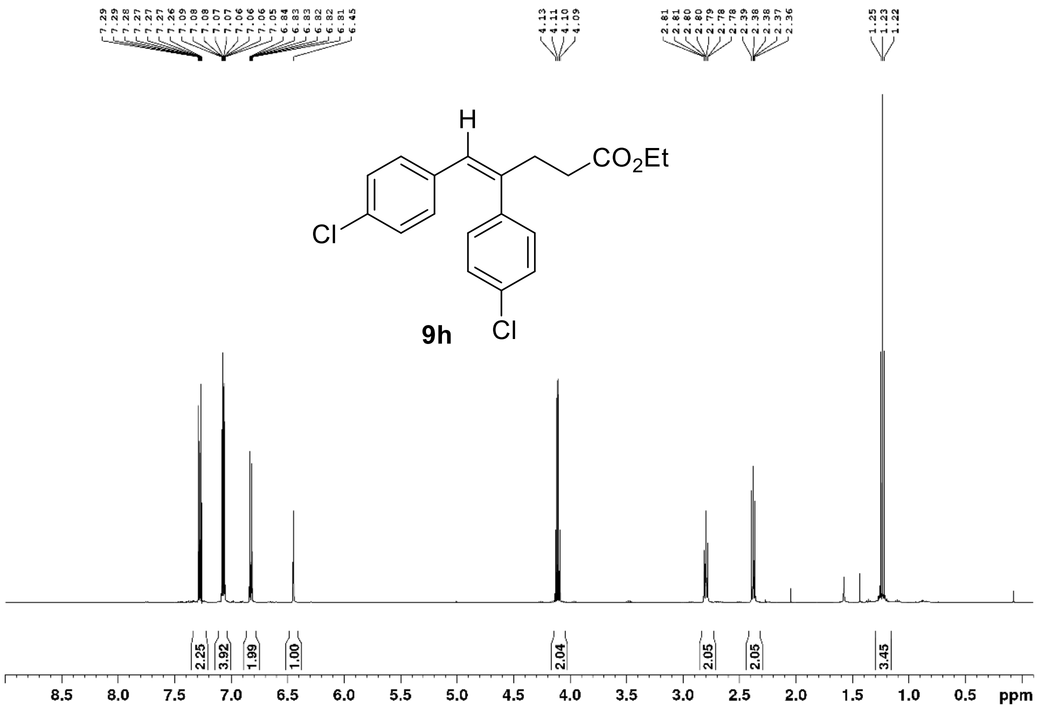
7.29 7.29 7.28 7.27 7.27 7.27 7.26 7.09 7.08 7.07 7.07 7.06 7.06 7.06 7.05 6.84 6.83 6.83 6.82 6.82 6.81 6.45
4.13 4.11 4.10 4.09
2.81 2.81 2.80 2.80 2.79 2.78 2.39 2.38 2.38 2.37 2.36
1.25 1.23 1.22
H
CO2Et
Cl
Cl
9h
2.25 3.92 1.99 1.00 2.04 2.05 2.05 3.45
8.5 8.0 7.5 7.0 6.5 6.0 5.5 5.0 4.5 4.0 3.5 3.0 2.5 2.0 1.5 1.0 0.5 ppm

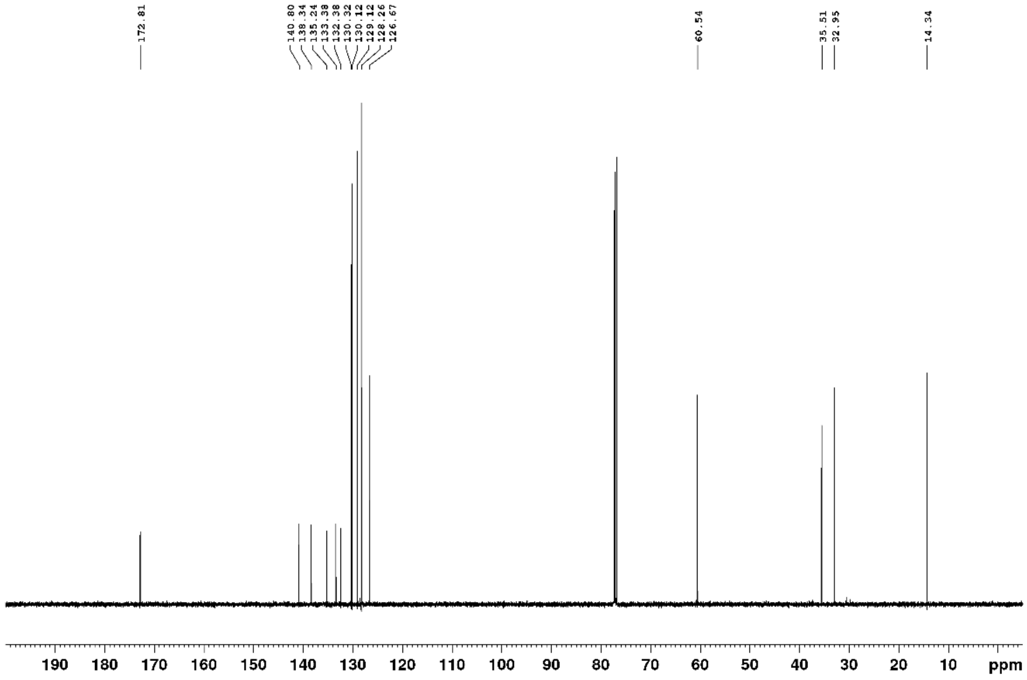
172.81
140.80 138.34 135.24 133.38 132.38 130.32 130.12 129.12 128.26 126.67
60.54
35.51 32.95
14.34
190 180 170 160 150 140 130 120 110 100 90 80 70 60 50 40 30 20 10 ppm

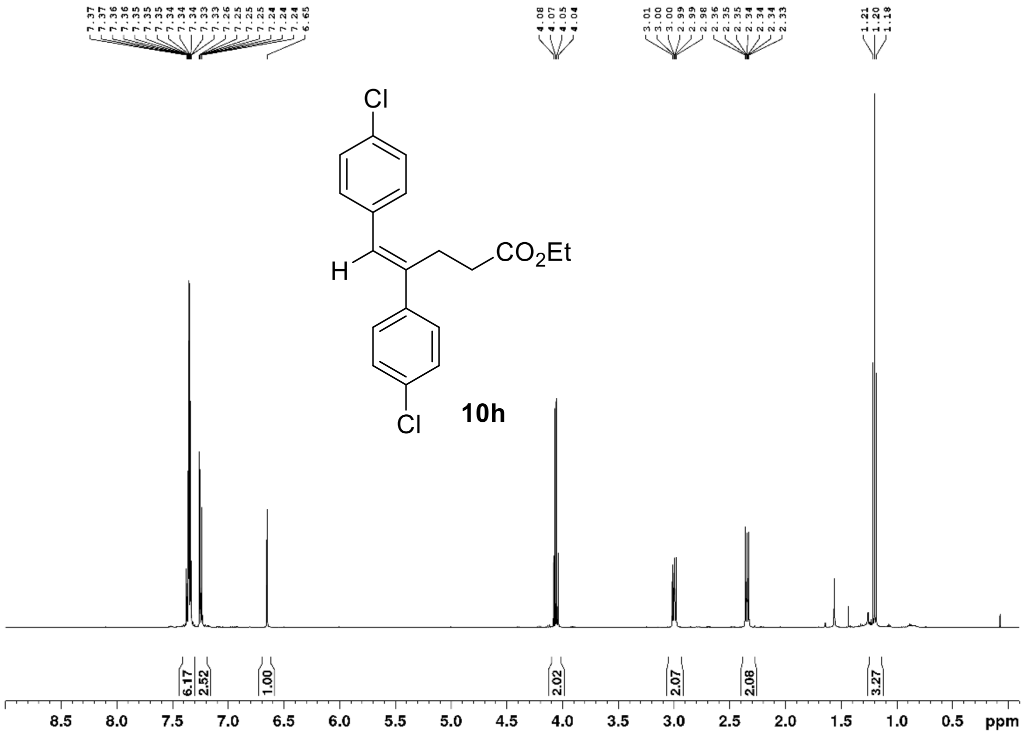
7.37
7.37
7.36
7.36
7.35
7.35
7.34
7.34
7.34
7.33
7.33
7.26
7.25
7.25
7.25
7.24
7.24
7.24
6.65
4.08
4.07
4.05
4.04
3.01
3.00
3.00
2.99
2.99
2.98
2.36
2.35
2.35
2.34
2.34
2.33
1.21
1.20
1.18
Cl
H
CO_2Et
Cl
10h
6.17
2.52
1.00
2.02
2.07
2.08
3.27
8.5 8.0 7.5 7.0 6.5 6.0 5.5 5.0 4.5 4.0 3.5 3.0 2.5 2.0 1.5 1.0 0.5 ppm

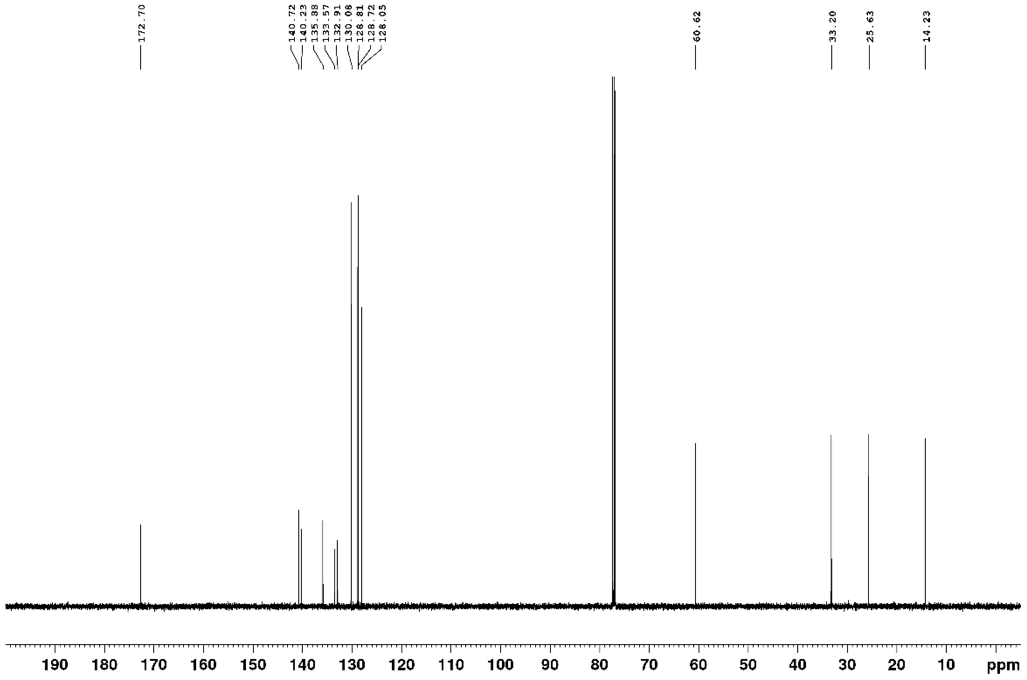
172.70
140.72
140.23
135.88
133.57
132.91
130.08
128.81
128.72
128.05
60.62
33.20
25.63
14.23
190 180 170 160 150 140 130 120 110 100 90 80 70 60 50 40 30 20 10 ppm

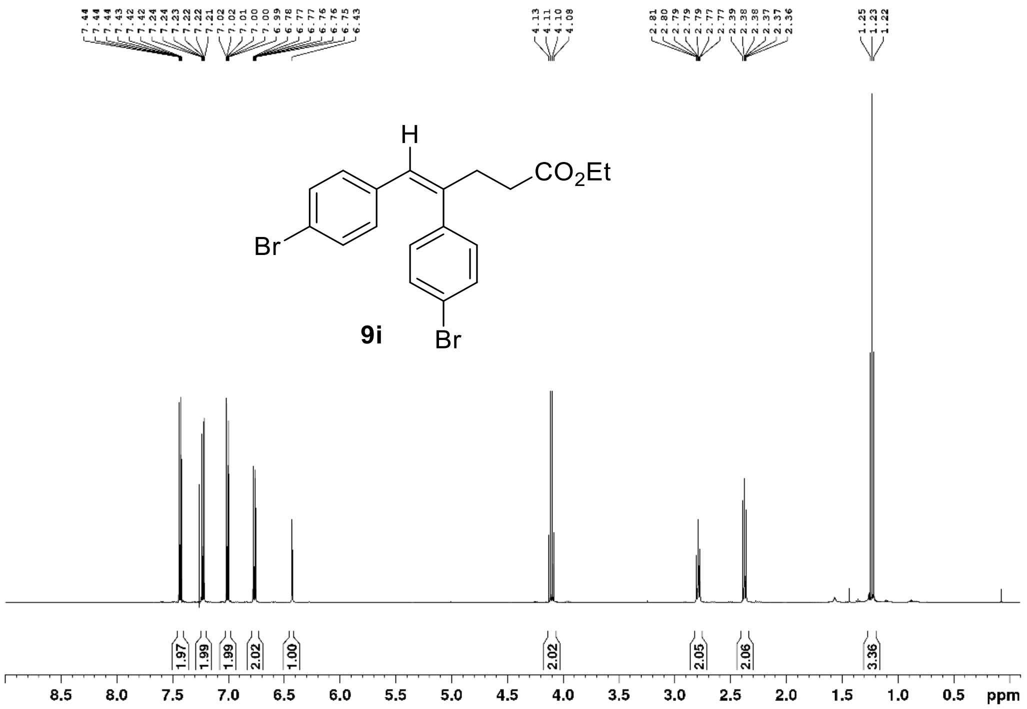
7.44
7.44
7.43
7.42
7.42
7.24
7.24
7.23
7.22
7.22
7.21
7.02
7.02
7.01
7.00
7.00
6.99
6.78
6.77
6.77
6.76
6.76
6.75
6.43
4.13
4.11
4.10
4.08
2.81
2.80
2.79
2.79
2.79
2.77
2.77
2.39
2.38
2.38
2.37
2.37
2.36
1.25
1.23
1.22
H
CO2Et
Br
Br
9i
1.97
1.99
1.99
2.02
1.00
2.02
2.05
2.06
3.36
8.5 8.0 7.5 7.0 6.5 6.0 5.5 5.0 4.5 4.0 3.5 3.0 2.5 2.0 1.5 1.0 0.5 ppm

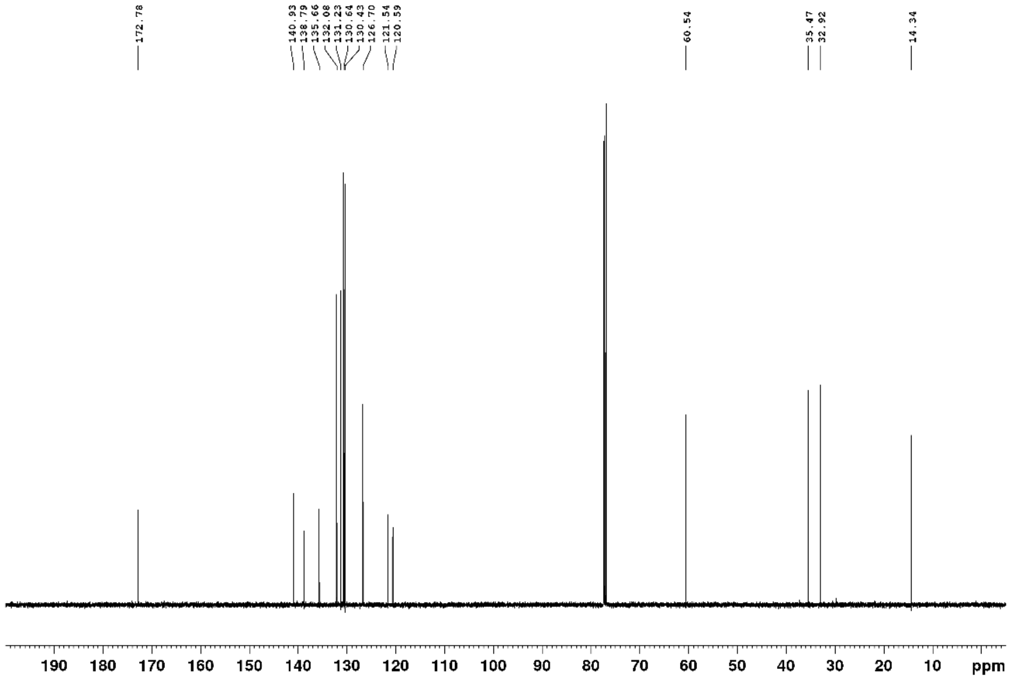
172.78
140.93
138.79
135.66
132.08
131.23
130.64
130.43
126.70
121.54
120.59
60.54
35.47
32.92
14.34
190 180 170 160 150 140 130 120 110 100 90 80 70 60 50 40 30 20 10 ppm

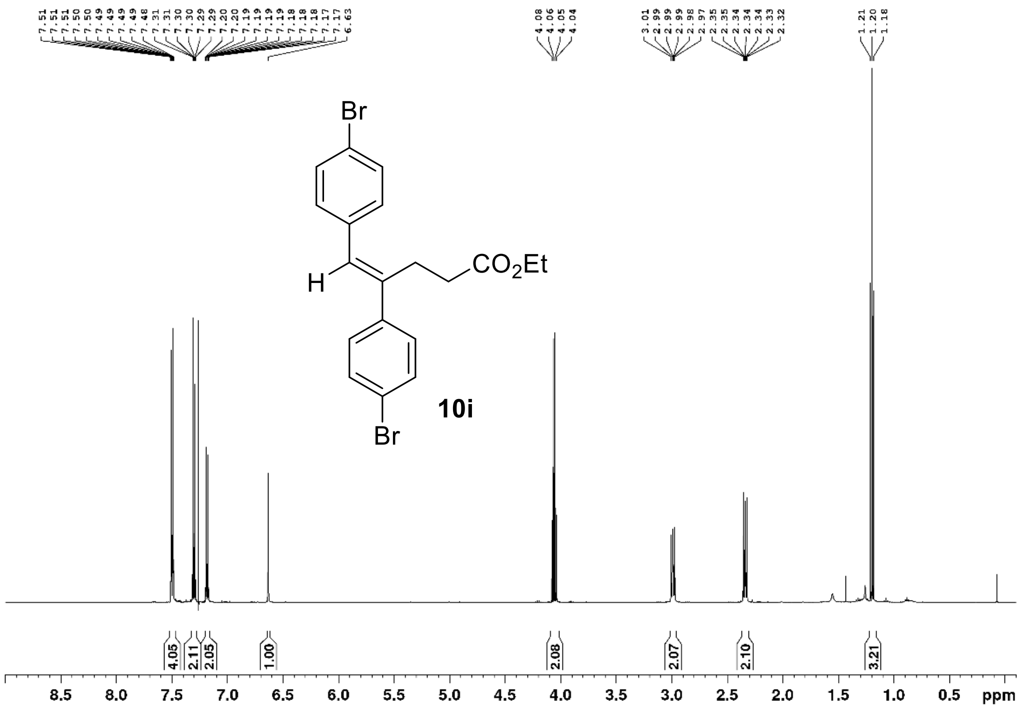
Br
H
CO2Et
Br
10i
4.05
2.11
2.05
1.00
2.08
2.07
2.10
3.21
8.5 8.0 7.5 7.0 6.5 6.0 5.5 5.0 4.5 4.0 3.5 3.0 2.5 2.0 1.5 1.0 0.5 ppm

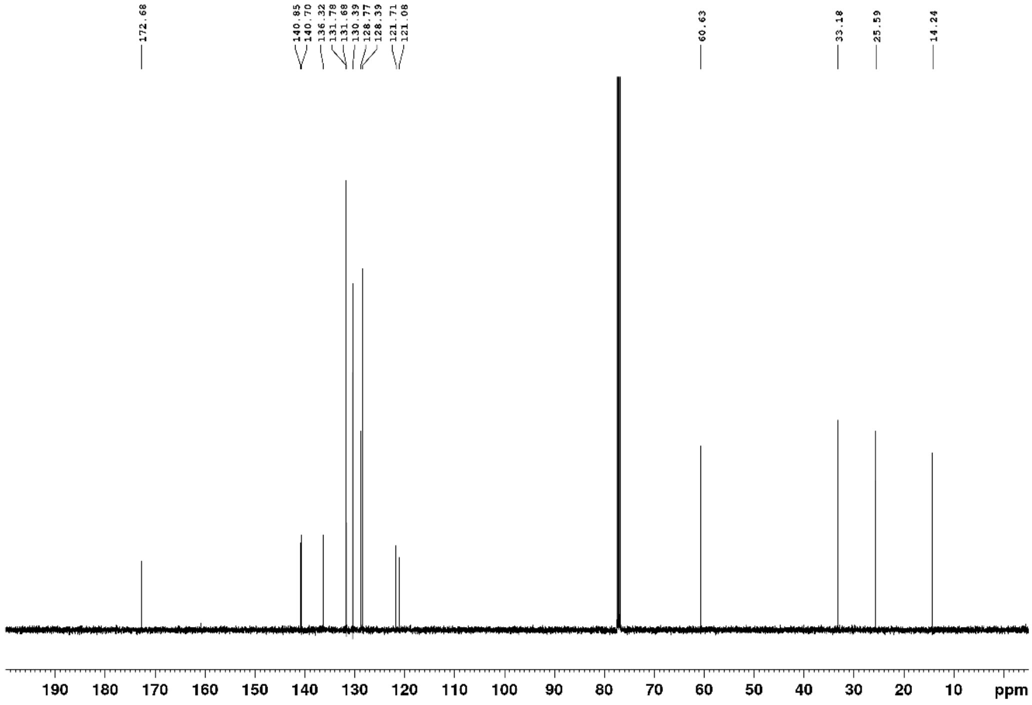
172.68
60.63
33.18
25.59
14.24
190 180 170 160 150 140 130 120 110 100 90 80 70 60 50 40 30 20 10 ppm

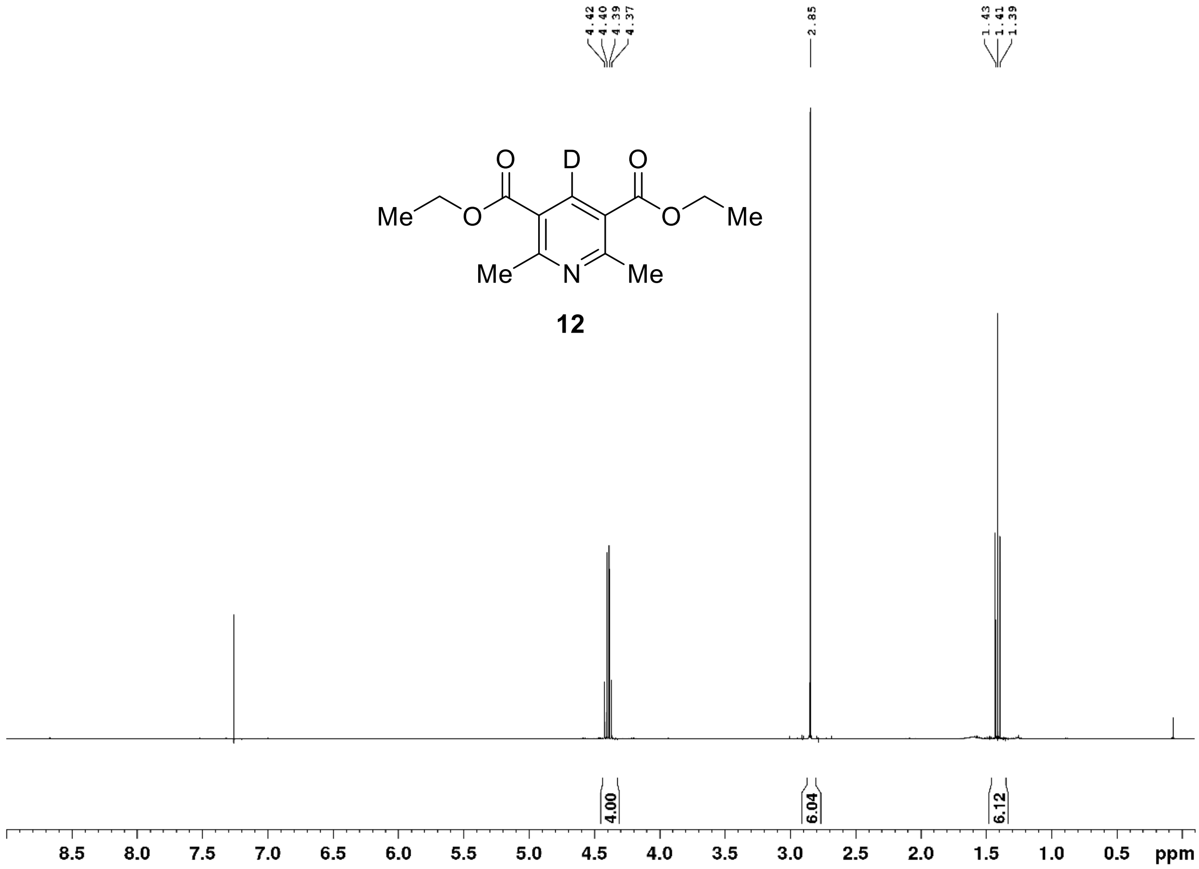
4.42
4.40
4.39
4.37
2.85
1.43
1.41
1.39
O
D
O
Me
O
O
Me
Me
N
Me
12
4.00
6.04
6.12
8.5 8.0 7.5 7.0 6.5 6.0 5.5 5.0 4.5 4.0 3.5 3.0 2.5 2.0 1.5 1.0 0.5 ppm

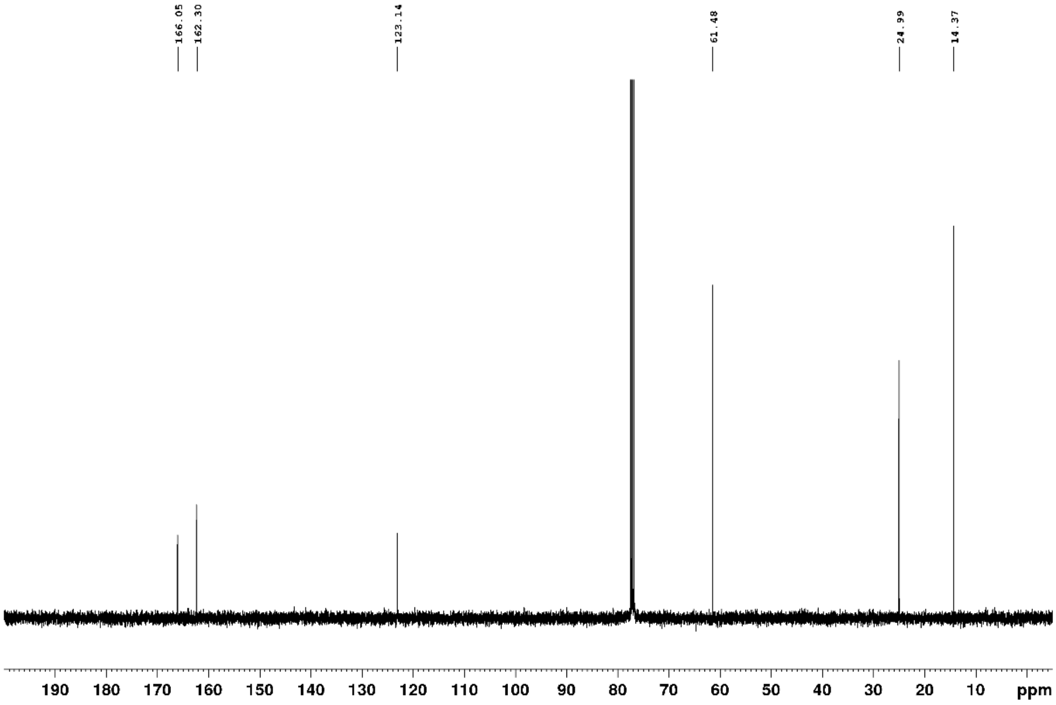
166.05
162.30
123.14
61.48
24.99
14.37
190 180 170 160 150 140 130 120 110 100 90 80 70 60 50 40 30 20 10 ppm

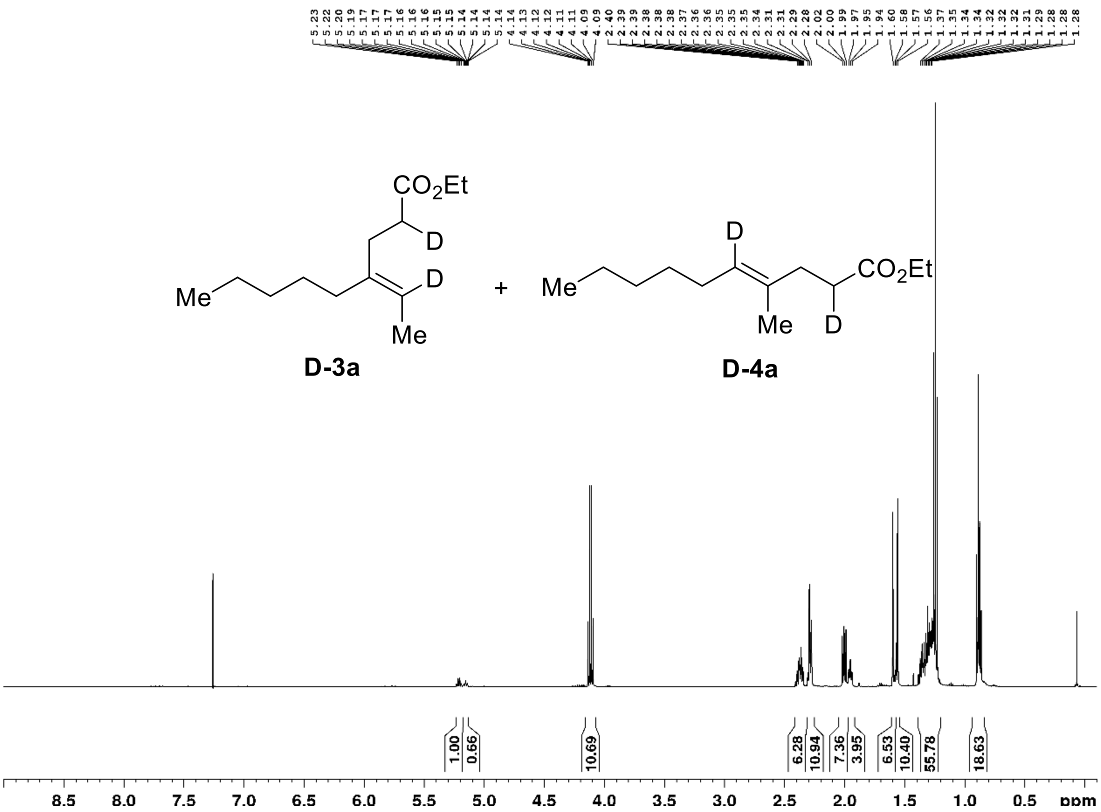
CO2Et
D
D
Me
Me
+
D
CO2Et
Me
Me
D
D-3a
D-4a

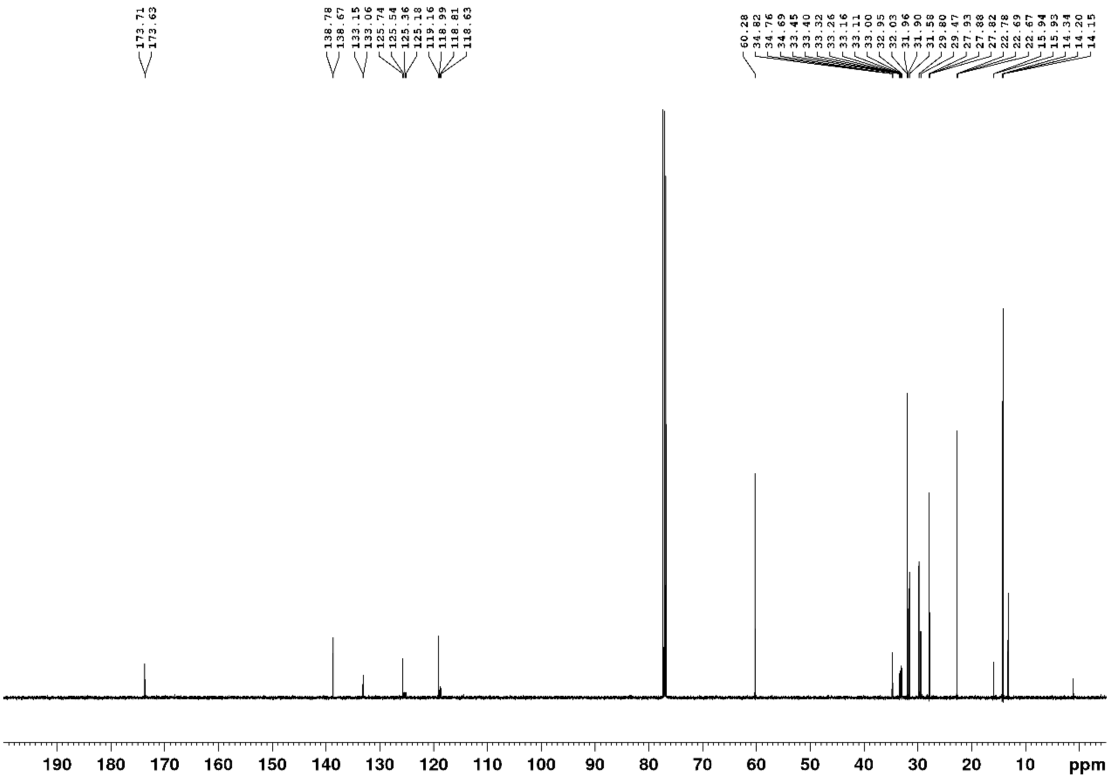

Supplement: Supplementary file 1 — Supplementary [file CHEM-25-15746-s001.pdf]
